# The Lipocalin Apolipoprotein D Functional Portrait: A Systematic Review

**DOI:** 10.3389/fphys.2021.738991

**Published:** 2021-10-07

**Authors:** Diego Sanchez, Maria D. Ganfornina

**Affiliations:** Instituto de Biologia y Genetica Molecular, Unidad de Excelencia, Universidad de Valladolid-Consejo Superior de Investigaciones Cientificas, Valladolid, Spain

**Keywords:** protein physiology, lipid peroxidation, membrane management, oxidative stress, lipoprotein particles, extracellular vesicles, lysosome, ApoD

## Abstract

Apolipoprotein D is a chordate gene early originated in the Lipocalin protein family. Among other features, regulation of its expression in a wide variety of disease conditions in humans, as apparently unrelated as neurodegeneration or breast cancer, have called for attention on this gene. Also, its presence in different tissues, from blood to brain, and different subcellular locations, from HDL lipoparticles to the interior of lysosomes or the surface of extracellular vesicles, poses an interesting challenge in deciphering its physiological function: Is ApoD a moonlighting protein, serving different roles in different cellular compartments, tissues, or organisms? Or does it have a unique biochemical mechanism of action that accounts for such apparently diverse roles in different physiological situations? To answer these questions, we have performed a systematic review of all primary publications where ApoD properties have been investigated in chordates. We conclude that ApoD ligand binding in the Lipocalin pocket, combined with an antioxidant activity performed at the rim of the pocket are properties sufficient to explain ApoD association with different lipid-based structures, where its physiological function is better described as lipid-management than by long-range lipid-transport. Controlling the redox state of these lipid structures in particular subcellular locations or extracellular structures, ApoD is able to modulate an enormous array of apparently diverse processes in the organism, both in health and disease. The new picture emerging from these data should help to put the physiological role of ApoD in new contexts and to inspire well-focused future research.

## Introduction

ApoD, identified and named almost 50 years ago, is a protein belonging to the Lipocalin family. Experimental research on ApoD has been accumulating, encouraged by numerous findings of ApoD relationship to many human diseases, from cancer to cardiovascular, metabolic or neurodegenerative conditions. This affluence of scientific reports has described many aspects of ApoD functional features, but a fundamental question remains to be responded: does ApoD moonlight, performing different biochemical functions in different biological contexts? or does it display a distinctive biochemical role that is being used in several physiological systems?

Along this half-a-century of ApoD research many reviews focused on this protein have been published. All of them are narrative in nature and many concentrate on specific details of ApoD such as its relationship to disease, often underlining partial and not critically assessed views on many aspects of ApoD biology.

Aiming at answering the central questions posed above, we have performed a systematic review of all primary research published until January 2021 where ApoD properties have been investigated in the chordate phylum. Reports were tagged and classified according to their contributions to “molecular properties,” “gene data,” “regulation of expression,” “disease-related,” “cellular trafficking,” “tissue and organ function,” and “protein physiology.” The level and quality of experimental evidence were critically evaluated to try to identify cause-effect relationships. The picture emerging from this approach should help to understand the physiological role of ApoD and to inspire well-focused future research.

## Methods

**Figure 1:**
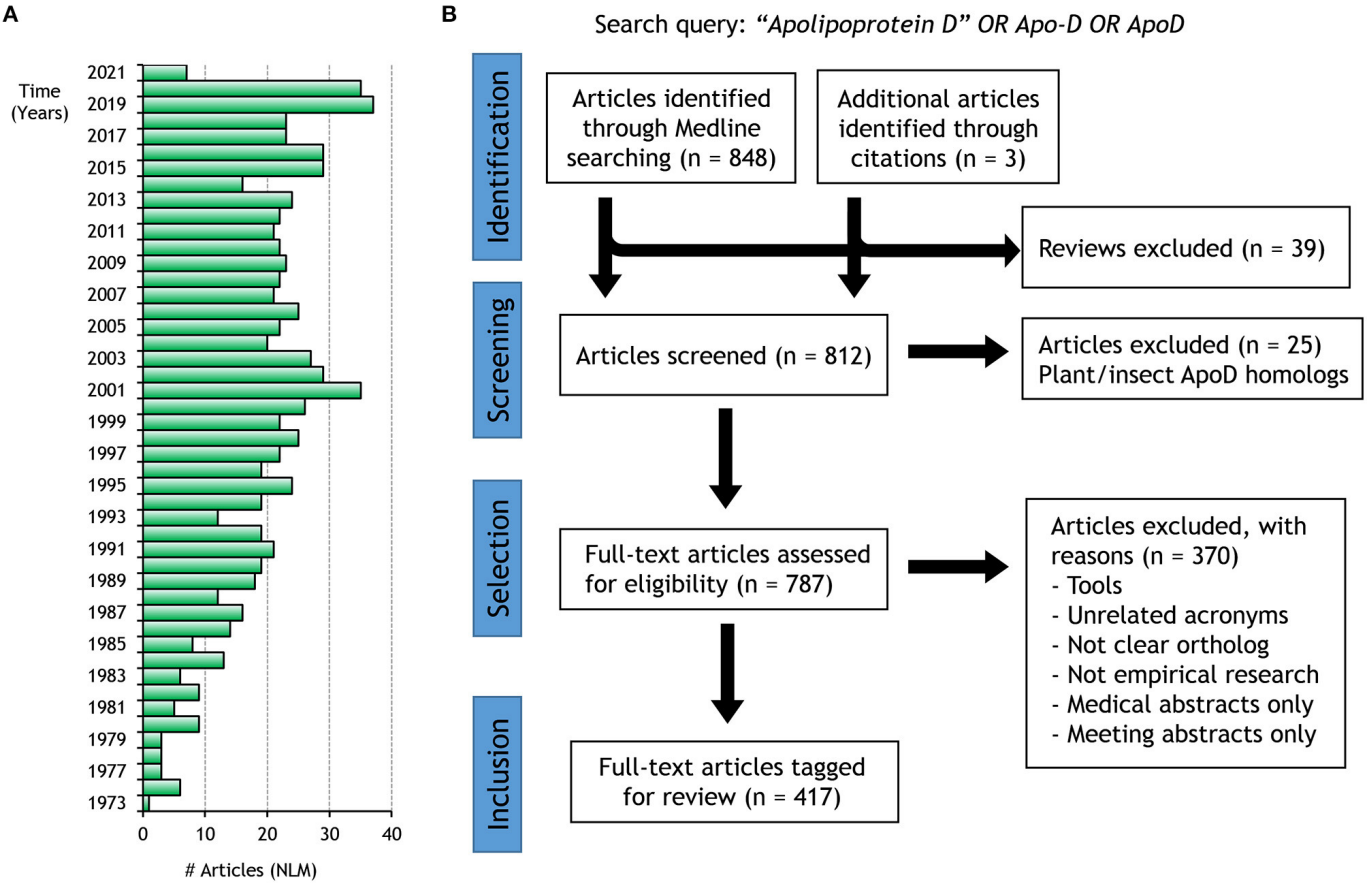
ApoD literature search and inclusion criteria. **(A)** Yearly timeline of articles recovered by PubMed using the search query designed for our review. **(B)** PRISMA flow diagram for record inclusion in our review.

To assess the current knowledge on the physiology of ApoD, we performed a literature review of primary publications in a systematic manner, searching the National Library of Medicine database with the PubMed engine (published until January 31, 2021). Using the search query “Apolipoprotein D” OR Apo-D OR ApoD, we recovered 851 entries from which 39 narrative reviews were excluded. Following a Title/Keywords screen, we selected 787 entries for further assessment. Following exclusion criteria we finally selected 417 articles reporting research on the Lipocalin ApoD in chordates as the final review sources, and stored them in a Zotero (v5.0.88) collection. The complete collection is available in the Supplementary Tables 1–8. According to the abstract information, articles were tagged with the following terms: Disease-related (DR, *n* = 216), Regulation of Expression (RE, *n* = 186), Gene Data (GD, *n* = 37), Molecular Properties (MP, *n* = 59), Cellular Trafficking (CT, *n* = 33), Tissue and Organ Function (TOF, *n* = 36), and Protein Physiology (PP, *n* = 35). Figure 1 summarizes the review process workflow and outcome.

Following full article reading, we classified each tagged report with subheading terms to guide the organization of the review. We then performed an evaluation of the conclusions statements of each report based on technical and argumentative consistency, according to existing state-of-the-art standards and required experimental controls. In cases of uncertainty, experts in each field were contacted and asked for their objective judgement.

The following databases and *in silico* prediction platforms and tools were used in this work: ProtParam (https://web.expasy.org/protparam/); DeepLoc-1.0 (http://www.cbs.dtu.dk/services/DeepLoc/); Gene Ontology database (http://geneontology.org/); Human Protein Atlas (https://www.proteinatlas.org); Mouse gene expression (http://www.informatics.jax.org/expression.shtml); miRNA database (mirdb.org). The ApoD multiple sequence alignment was generated with ClustalX2 (http://www.clustal.org), and the 3D structure of ApoD was visualized with ViewerLite 4.2 (https://chemweb.ir/accelrys-viewerlite/). A model of HApoD with sugars attached was built with GlyProt (http://glycosciences.de/modeling/glyprot/php/main.php).

## Results and Discussion

**Figure 2:**
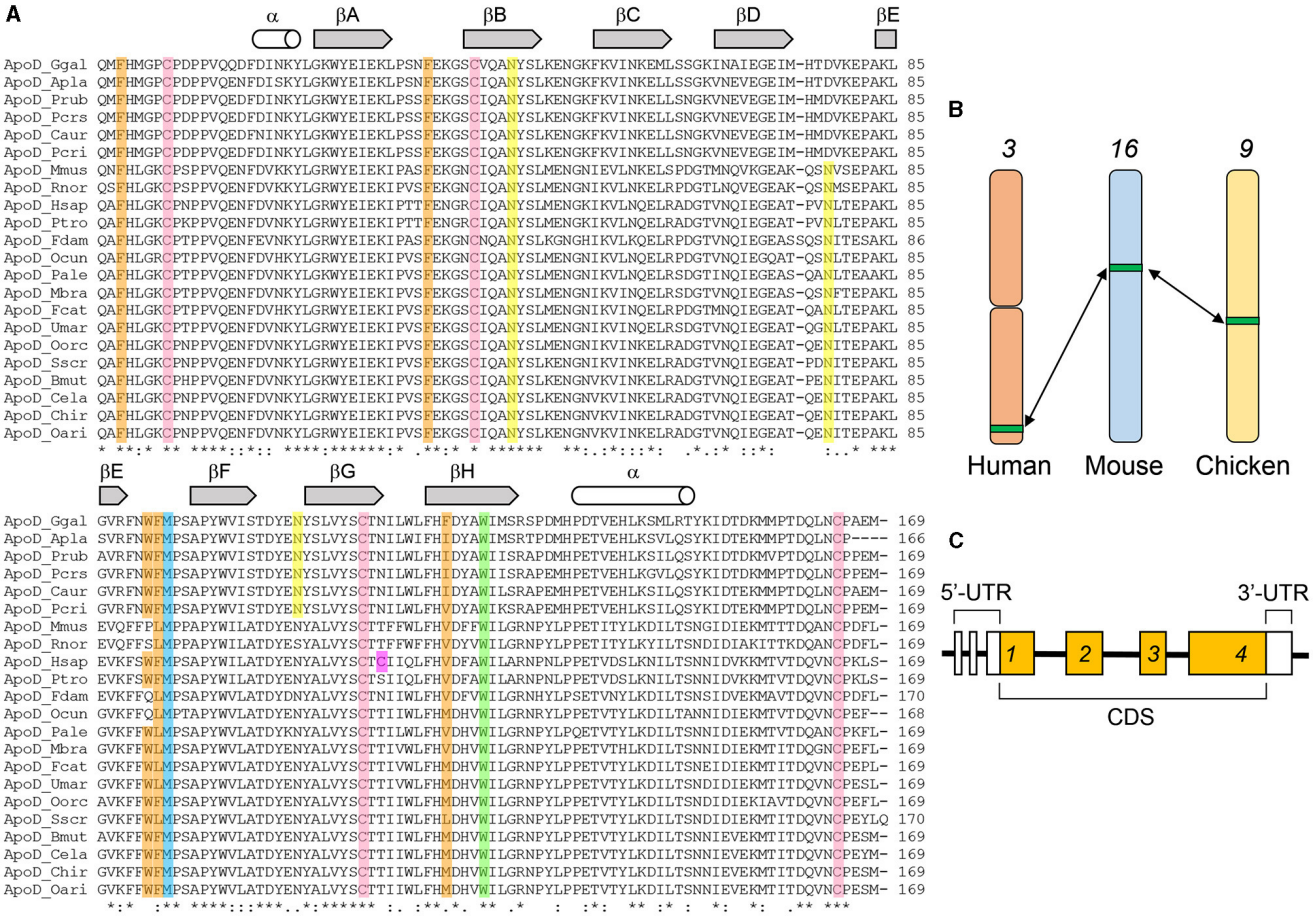
ApoD protein sequence and gene features. **(A)** Multiple sequence analysis (MSA) of the mature amino acid sequence of selected vertebrate species recovered from GenBank (Apla, Anas platyrhynchos_EOB05196.1; Caur, Cathartes aura_KFP53002.1; Ggal, Gallus gallus_NP001011692.1; Pcri, Pelecanus crispus_KFQ60274.1; Pcrs, Podiceps cristatus_KFZ69168.1; Prub, Phoenicopterus ruber_KFQ85568.1; Bmut, Bos mutus_ELR54927.1; Cela, Cervus elaphus_ABB77207.1; Chir, Capra hircus_XP005675150.1; Oari, Ovis aries_XP004003075.1; Sscr, Sus scrofa_XP001926098.2; Fcat, Felis catus_XP006936237.1; Umar, Ursus marinus_XP008706566.1; Oorc, Orcinus orca_XP004278821.1; Mbra, Myotis brandtii_EPQ12038.1; Pale, Pteropus Alecto_XP006906222.1; Ocun, Oryctolagus cuniculus_ NP001075727.1; Hsap, Hoo sapiens_ NP001638.1; Ptro, Pan troglodites_XP516965.1; Fdam, Fukomys damarensis_KFO33128.1; Mmus, Mus musculus_CAA57974.1; Rnor, Rattus norvegicus_NP036909.1). Asterisks represent identical residues in all sequences, and dots/double dots point to similar residues. α-helices and β-strands are shown on top of the MSA, based on the solved tertiary structure of human ApoD. Colored residues are: four conserved cysteines involved in intramolecular disulfide bonds (pink), the human-specific unpaired cysteine (purple), conserved tryptophan residue in the ligand binding pocket (green), two glycosylated Asn residues (yellow), the antioxidant Met residue (blue), and residues in the hydrophobic surface patches at the rim of the binding pocket (orange). **(B)** Schematic representation of the chromosomal location of ApoD gene in human, mouse and chicken genomes. **(C)** Schematic representation of a consensus gene architecture for chordate ApoD, with four coding sequence (CDS)-containing exons and several 5′-UTR exons.

ApoD is an early-diverging member of the Lipocalin family, with its phylogenetic origins traced back to the origin of chordates (Ganfornina et al., 2000; Diez-Hermano et al., 2021). Furthermore, ApoD is the chordate Lipocalin most similar to those in other phyla. ApoD primary structure is well-conserved in chordates, as deduced from a multiple sequence alignment of 22 chordate species (Figure 2A; Table 1), with an average 67% identity (range: 55–90%) in mature protein sequence. An intriguing aspect of this alignment is a favored residue conservation of the region encompassing the first three β-strands of the protein primary structure (Figure 2A).

**Table 1:** ApoD protein parameters.

**Acc. number**	**Class/order**	**Common name**	**Species name**	**# Residues mature protein**	**Theor. Mw**	**Theor. pI**	**# Disulfide bonds**	**# Cys**	**N-linked sugars**	**Met-93**	**Mature protein sequence**
EOB05196.1	Aves/Anseriformes	Mallard	*Anas platyrhynchos*	166	19,412	5.35	2	4	2	Yes	QMFHMGPCPDPPVQENFDISKYLGKWYEIEKLPSNFEKGSCIQANYSLKENGKFKV INKELLSSGKVNEVEGEIMHTDVKEPAKLSVRFNWFMPSAPYWVISTDYENYSLV YSCTNILWIFHIDYAWIMSRTPDMHPETVEHLKSVLQSYKIDTEKMMPTDQLNCP
KFP53002.1	Aves/Cathartiformes	Turkey vulture	*Cathartes aura*	169	19,750	5.23	2	4	2	Yes	QMFHMGPCPDPPVQEDFNINKYLGKWYEIEKLPSSFEKGSCIQANYSLKENGK FKVINKELLSNGKVNEVEGEIMHMDVKEPAKLGVRFNWFMPSAPYWVISTDYENYSLVYSCTNILW LFHIDYAWILSRAPEMHPETVEHLKSILQSYKIDTEKMMPTDQLNCPAEM
NP001011692.1	Aves/Galliformes	Chicken	*Gallus gallus*	169	19,780	5.51	2	4	2	Yes	QMFHMGPCPDPPVQQDFDINKYLGKWYEIEKLPSNFEKGSCVQANYSLKENGK FKVINKEMLSSGKINAIEGEIMHTDVKEPAKLGVRFNWFMPSAPYWVISTDYEN YSLVYSCTNILWLFHFDYAWIMSRSPDMHPDTVEHLKSMLRTYKIDTDKMMPTDQLNCPAEM
KFQ60274.1	Aves/Pelecaniformes	Dalmatian pelican	*Pelecanus crispus*	169	19,778	5.23	2	4	2	Yes	QMFHMGPCPDPPVQEDFDINKYLGKWYEIEKLPSSFEKGSCIQANYSLKENGKFK VINKELLSNGKVNEVEGEIMHMDVKEPAKLGVRFNWFMPSAPYWVISTDYENYSLVYSCTNILWLFH VDYAWIKSRAPEMHPETVEHLKSILQSYKIDTEKMMPTDQLNCPPEM
KFZ69168.1	Aves/Podicipediformes	Great crested grebe	*Podiceps cristatus*	169	19,719	5.09	2	4	2	Yes	QMFHMGPCPDPPVQEDFDINKYLGKWYEIEKLPSSFEKGSCIQANYSLKENGKFKVIN KELLSNGKVNEVEGEIMHMDVKEPAKLGVRFNWFMPSAPYWVISTDYENYSLVYSCTNILWL FHIDYAWIISRAPEMHPETVEHLKGVLQSYKIDTDKMMPTDQLNCPPEM
KFQ85568.1	Aves/Phoenicopteriformes	American flamingo	*Phoenicopterus ruber*	169	19,731	5.08	2	4	2	Yes	QMFHMGPCPDPPVQEDFDINKYLGKWYEIEKLPSSFEKGSCIQANY SLKENGKFKVINKELLSNGKVNEVEGEIMHMDVKEPAKLAVRFNWFMPSAPYWVISTDYENYSLVYSCTNILWLFHIDYAWI ISRAPDMHPETVEHLKSILQSYKIDTDKMVPTDQLNCPPEM
ELR54927.1	Mammalia/Artiodactyla	Wild yak	*Bos mutus*	169	19,466	5.07	2	4	2	Yes	QAFHLGKCPHPPVQENFDVNKYLGKWYEIEKIPVSFEKGSCIQANYSLKENGNVKVINK ELRADGTVNQIEGEATPENITEPAKLAVKFFWFMPSAPYWVLATDYENYALVYSCTT IIWLFHMDHVWILGRNPYLPPETVTYLKDILTSNNIEVEKMTITDQVNCPESM
ABB77207.1	Mammalia/Artiodactyla	Red deer	*Cervus elaphus*	169	19,564	4.96	2	4	2	Yes	QAFHLGKCPNPPVQENFDVNKYLGRWYEIEKIPVSFEKGSCIQANYSLKENGNV KVINKELRADGTVNQIEGEATQENITEPAKLGVKFFWFMPSAPYWVLATDYENYALVYSCTTIIWLF HMDHVWILGRNPYLPPETVTYLKDILTSNNIEVEKMTITDQVNCPEYM
XP005675150.1	Mammalia/Artiodactyla	Goat	*Capra hircus*	169	19,488	4.96	2	4	2	Yes	QAFHLGKCPNPPVQENFDVNKYLGRWYEIEKIPVSFEKGSCIQANYSLKENGNVKV INKELRADGTVNQIEGEATQENITEPAKLGVKFFWFMPSAPYWVLATDYENYALVYSCT TIIWLFHMDHVWILGRNPYLPPETVTYLKDILTSNNIEVEKMTITDQVNCPESM
XP004003075.1	Mammalia/Artiodactyla	Sheep	*Ovis aries*	169	19,488	4.96	2	4	2	Yes	QAFHLGKCPNPPVQENFDVNKYLGRWYEIEKIPVSFEKGSCIQANYSLKENGNVKVINKE LRADGTVNQIEGEATQENITEPAKLGVKFFWFMPSAPYWVLATDYENYALVYSCTTIIWL FHMDHVWILGRNPYLPPETVTYLKDILTSNNIEVEKMTITDQVNCPESM
XP001926098.2	Mammalia/Artiodactyla	Swine	*Sus scrofa*	170	19,592	4.83	2	4	2	Yes	QAFHLGKCPNPPVQENFDVNKYLGRWYEIEKIPVSFEKGSCIQANYSLKENGNIKVIN KELRADGTVNQIEGEATPDNITEPAKLGVKFFWLMPSAPYWVLATDYENYALVYSCTTIIWLFHLDHV WILGRNPYLPPETVTYLKDILTSNDIDIEKMTITDQVNCPEYLQ
XP006936237.1	Mammalia/Carnivora	Domestic cat	*Felis catus*	169	19,474	4.82	2	4	2	Yes	QAFHLGKCPTPPVQENFDVHKYLGRWYEIEKIPVSFEKGSCIQANYSLMENGNIKV INQELRPDGTMNQIEGEATQANLTEPAKLGVKFFWLMPSAPYWVLATDYENYALVYSCTTIV WLFHMDHVWILGRNPYLPPETVTYLKDILTSNEIDIEKMTITDQVNCPEPL
XP008706566.1	Mammalia/Carnivora	Polar bear	*Ursus maritimus*	169	19,371	4.71	2	4	2	Yes	QAFHLGKCPTPPVQENFDVNKYLGRWYEIEKIPVSFEKGSCIQANYSL MENGNIKVINQELRSDGTVNQIEGEATQGNLTEPAKLGVKFFWLMPSAPYWVLAT DYENYALVYSCTTIVWLFHMDHVWILGRNPYLPPETVTYLKDILTSNDIDIEKMTITDQVNCPESL
XP004278821.1	Mammalia/Cetacea	Killer whale	*Orcinus orca*	169	19,500	4.74	2	4	2	Yes	QAFHLGKCPNPPVQENFDVNKYLGRWYEIEKIPVSFEKGSCIQANYSLMENGNIK VINKELRADGTVNQIEGEATQENITEPAKLAVKFFWFMPSAPYWVLATDYENYALV YSCTTIIWLFHMDHVWILGRNPYLPPETVTYLKDILTSNDIDIEKIAVTDQVNCPEFL
EPQ12038.1	Mammalia/Chiroptera	Brandt's bat	*Myotis brandtii*	169	19,380	4.9	2	4	2	Yes	QAFHLGKCPTPPVQENFDVNKYLGRWYEIEKIPVSFEKGSCIQANYSLM ENGNIKVINQELRSDGTVNQIEGEASQSNFTEPAKLGVKFFWLMPSAPY WVLATDYENYALVYSCTTIVWLFHVDHVWILGRNPYLPPETVTHLKDILTSNNIDIEKMTITDQGNCPEFL
XP006906222.1	Mammalia/Chiroptera	Black flying fox	*Pteropus alecto*	169	19,359	5.35	2	4	2	Yes	QAFHLGKCPTPPVQENFDVNKYLGKWYEIEKIPVSFEKGSCIQANYSLMENGNIKVLNQE LRSDGTINQIEGEASQANLTEAAKLGVKFFWLMPSAPYWVLATDYKNYA LVYSCTTILWLFHVDHVWILGRNPYLPQETVTYLKDILTSNNIDIEKMTVTDQANCPKFL
NP001075727.1	Mammalia/Lagomorpha	Rabbit	*Oryctolagus cuniculus*	168	19,433	5.15	2	4	2	Yes	QAFHLGRCPTPPVQENFDVHKYLGRWYEIEKIPVSFEKGNCIQANYS LMENGNIKVLNQELRPDGTVNQIEGQATQSNLTEPAKLGVKFFQLMPTAPYWVL ATDYENYALVYSCTTIIWLFHMDHVWILGRNRYLPPETVTYLKDILTANNIDIEKMTVTDQVNCPEF
NP001638.1	Mammalia/Primates	Human	*Homo sapiens*	169	19,303	5.2	2	5	2	Yes	QAFHLGKCPNPPVQENFDVNKYLGRWYEIEKIPTTFENGRCIQAN YSLMENGKIKVLNQELRADGTVNQIEGEATPVNLTEPAKLEVKFSWFMPSAPYWILATD YENYALVYSCTCIIQLFHVDFAWILARNPNLPPETVDSLKNILTSNNIDVKKMTVTDQVNCPKLS
XP516965.1	Mammalia/Primates	Chimpanzee	*Pan troglodytes*	169	19,301	5.43	2	4	2	Yes	QAFHLGKCPKPPVQENFDVNKYLGRWYEIEKIPTTFENGRCIQANYSLMENGKIKV LNQELRADGTVNQIEGEATPVNLTEPAKLEVKFSWFMPSAPYWILATDYENYALVYS CTSIIQLFHVDFAWILARNPNLPPETVDSLKNILTSNNIDVKKMTVTDQVNCPKLS
KFO33128.1	Mammalia/Rodentia	Damaraland mole-rat	*Fukomys damarensis*	170	19,458	5.16	2	4	2	Yes	QAFHLGKCPTPPVQENFEVNKYLGRWYEIEKIPASFEKGNCNQANYSLKGNGHIKVL KQELRPDGTVNQIEGEASSQSNITESAKLEVKFFQLMPSAPYWVLATDYDNYALVY SCTNIIWLFHVDFVWILGRNHYLPSETVNYLKDILTSNSIDVEKMAVTDQVNCPDFL
CAA57974.1	Mammalia/Rodentia	House mouse	*Mus musculus*	169	19,478	4.71	2	4	2	Yes	QNFHLGKCPSPPVQENFDVKKYLGRWYEIEKIPASFEKGNCIQANYSLMENGNIE VLNKELSPDGTMNQVKGEAKQSNVSEPAKLEVQFFPLMPPAPYWILATDYENYALVYSCTTFFWL FHVDFFWILGRNPYLPPETITYLKDILTSNGIDIEKMTTTDQANCPDFL
NP036909.1	Mammalia/Rodentia	Rat	*Rattus norvegicus*	169	19,584	5.04	2	4	2	Yes	QSFHLGKCPSPPVQENFDVKKYLGRWYEIEKIPVSFEKGNCIQANYSLMENGN IKVLNKELRPDGTLNQVEGEAKQSNMSEPAKLEVQFFSLMPPAPYWILATDYES YALVYSCTTFFWFFHVDYVWILGRNPYLPPETITYLKYILTSNDIDIAKITTKDQANCPDFL
			Min	166	19,301	4.71					
			Max	170	19,780	5.51					
			**Average**	**169**	**19,519**	**5.07**					

*In silico prediction (see section Methods) of molecular weight, pI and N-linked oligosaccharides, or experimentally tested (disulfide bonds and antioxidant Met-93 of human ApoD). ApoD from birds and mammals analyzed*.

### Molecular Properties

ApoD is a monodomain globular glycoprotein with two intramolecular disulfide bonds, which are molecular properties suitable for working in extracellular non-reducing milieus. ApoD shows an N-terminal signal peptide in all chordates that lets the nascent protein to enter the endoplasmic reticulum. The protein can therefore follow a canonical secretion pathway, and is glycosylated along this path.

#### Protein Parameters

**Figure 3:**
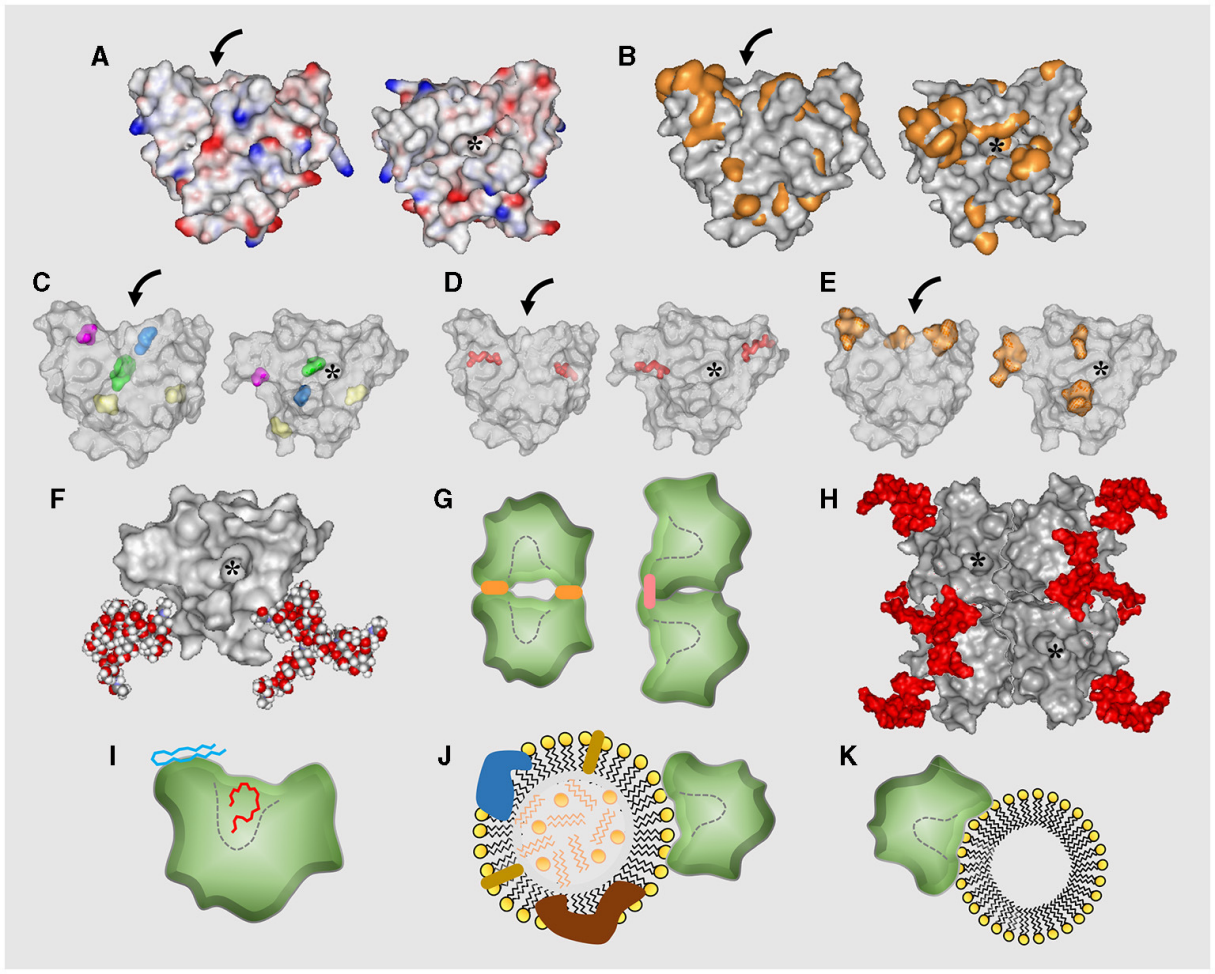
Molecular features of ApoD. **(A,B)** Graycolored space-filled views of the human ApoD tertiary structure (modelled from PDB ID:2HZQ) showing charged residues in A (positive, red; negative, blue) and hydrophobic residues in B (orange). Side view of the β-barrel (left image; curved arrows point to the pocket entrance) and top view (right image) looking into the hydrophobic pocket (asterisk). **(C–E)** Human ApoD (PDB ID:2HZQ) side and top views with highlighted relevant residues. Colored residues in **(C)** are the antioxidant Met93 (blue); the human-specific unpaired Cys116 (purple); the conserved ligand binding pocket Trp127 (green); and the two glycosylated Asn45/Asn78 (yellow). Pink-colored residues in D are the four cysteines forming two intramolecular disulfide bonds. Orange-colored residues in E are those forming three hydrophobic loops around the pocket entrance. **(F)** Space-filled view of human ApoD with reported oligosaccharides linked to Asn45 and Asn78, as modelled by GlyProt (see Methods). **(G)** Cartoon representations of human ApoD dimers formed by hydrophobic patches (orange) or by intermolecular Cys116 disulfide bonds (pink). Variations of the particular configuration shown are possible. Dashed lines delineate the ligand pocket. **(H)** Representation of the best supported tetrameric structure of human ApoD found in BCF. Asterisks mark the ligand pocket accessible in all subunits (two facing back). Oligosaccharides shown in red. **(I)** Cartoon illustration of a side view of human ApoD with AA (red) and HpETE (blue) positioned into the hydrophobic pocket (marked by a dashed line) and interacting with the Met93-containing hydrophobic patch respectively. **(J,K)** Cartoon illustration of human ApoD interacting with higher-order lipid structures via the hydrophobic patches at rim of the pocket; **(J)** HDL particle; **(K)** Unilamellar vesicle (liposome).

Since early characterization studies of ApoD, its apparent electrophoretic mobility, density of ApoD-positive fractions and behavior in size exclusion chromatography, suggested the existence of post-translational modifications (glycosylation), a potential for oligomerization, and an association with lipids. The predicted acidic isoelectric point (Table 1) implicates that ApoD polypeptide would have its lowest solubility in aqueous-salt solutions at the pH of acidic organelles in the cell, while at neutral pH the ApoD polypeptide would show a net negative charge. ApoD displays four conserved cysteine residues, while an additional cysteine (Cys116) is present only in humans (absent even in other primates) and allows for inter-molecular disulfide bond formation (Figures 2A, 3B,E; Table 1).

References contributing to this section are listed in **Reference Collection 1**, Supplementary Table 2.

#### Protein Structure

The ApoD 3D crystal structure has been solved for the human protein after modification of several residues that rendered the protein prone to aggregation. The unique human Cys116 is close to one of the hydrophobic loops, and was also mutated to facilitate crystallization. The structure reveals a typical Lipocalin fold (Skerra, 2000) composed of an eight-stranded β-barrel structure with an adjacent C-terminal α-helix. It has a closed end, and an open end with access to a pocket able to bind mostly hydrophobic ligands. Two intra-molecular disulfide bonds stabilize the structure. Three out of four loops at the barrel open-end are hydrophobic, making these regions candidate for interaction with hydrophobic surfaces, and contain residues relevant for ApoD antioxidant properties (see section Protein Physiology). Two N-glycosylation sites (Figures 2A, 3F) are located on the side and bottom of the calyx, away from the ligand-binding pocket opening. Figures 3A,B show a surface representation of the ApoD monomer structure with charged or hydrophobic surface highlighted in color. Other relevant residues are shown in Figures 3C–E.

The presence of a ligand inside the pocket did not modify the general crystal structure of ApoD. When explored by amide hydrogen-deuterium exchange mass spectrometry (HDX-MS) or small-angle X-ray scattering (SAXS) in solution, interesting conformational changes elicited by ligand binding were detected, resulting in further ordering of the already stable Lipocalin fold. ApoD structure is also stable upon protein oxidation with H_2_O_2_. Dynamic information extrapolated from the crystal structure has allowed further modeling of ApoD binding to small ligands, lipoprotein particles or membranes. These studies help to understand a methionine-dependent lipid antioxidant mechanism (see below) and to study the influence of glycosylation on these functional properties. In addition, the ApoD monomer crystal structure, combined with modeled glycosylation conformations, was used to generate coherent models for the conformations of ApoD oligomers (Figures 3G,H) later confirmed experimentally (see below).

References contributing to this section are listed in **Reference Collection 2**, Supplementary Table 2.

#### Protein Glycosylation

As mentioned above, sugars were soon revealed to be linked to ApoD, with a relevant carbohydrate contribution (~15–22%) to its apparent molecular weight. Two asparagine sites were experimentally demonstrated to be glycosylated, and *in silico* studies of human ApoD revealed no interference of sugars with binding pocket access. Figure 3F depicts a model of the N-linked oligosaccharides. The Asn45 glycosylation site is conserved in birds and mammals, but the second glycosylation site shows variations in position (Figure 2A). In ApoD of human plasma, Asn45 contains primarily trisialo-triantennary oligosaccharides, and Asn78 contains fucosylated disialo-biantennary oligosaccharides. The presence of negatively charged sialic acid in native ApoD sugar moiety contributes to its net negative charge in neutral pH environments.

Interesting variations of ApoD carbohydrate moiety have been reported between species (e.g., humans vs. other primates or rodents), between various tissues in a single species (brain tissue, cerebrospinal fluid, inner ear perilymph or plasma), within a tissue, or between health and disease conditions. Patterns of glycosylation have also been demonstrated to be sex-dependent (e.g., BCF in females, or axillary secretion in males). For example, less glycosylated forms of ApoD are present in mouse/human brain tissue compared to plasma, with differences in both terminal sialic acid and core N-linked oligosaccharides. A clear increase in α2-3 sialoglycosylation of plasma ApoD distinguishes, with high sensitivity, children with Autism Spectrum Disorder from healthy controls. Within a single tissue (cerebrospinal fluid; CSF) there is also variegation in the degree of ApoD sialylation. These variations generate size and charge heterogeneities with potential functional consequences worth exploring.

References contributing to this section are listed in **Reference Collection 3**, Supplementary Table 2.

#### Protein Oligomerization

Covalent and non-covalent homodimers and tetramers of ApoD have been detected in various experimental and biological systems. All studies of ApoD oligomerization have been focused so far on the human protein. Crystallization of bacterial recombinant human ApoD revealed that the protein tends to aggregate due to hydrophobic surface patches. This property could promote self-association or association with lipid-based structures *in vivo* (Figures 3J,K).

Homodimers due to intermolecular disulfide bonds, evidenced by comparing electrophoretic mobility under reducing/non-reducing conditions, have been detected in urine and tear fluids. Experiments with sulfhydryl-trapping reagents during handling indicate that new disulfide bonds were not introduced along the experimental procedure. However, other forms of oligomerization are possible and compatible with these results, resulting from non-covalent stable binding between ApoD monomers. Figures 3G,H summarize the different forms of ApoD self-interaction.

Exposure of human ApoD to oxidized lipids promote dimerization and further oligomerization, in a way dependent on the oxidation state of particular methionine residues (Met93, see below), as demonstrated with recombinant ApoD, mutated at specific Met residues, and produced in a human cell line. Oxidation-dependent ApoD dimerization is resistant to guanidine hydrochloride (GuHCl) but not to urea, indicating that it is based on non-covalent intermolecular bonds. This property has allowed to detect ApoD dimers in specific brain regions of Alzheimer's disease patients (e.g., hippocampus, but not cerebellum) that also correlate with disease progression. In contrast, GuHCl extracts from healthy control brains show only monomeric forms of ApoD.

A tetrameric stable form of native ApoD in BCF, but not in plasma or CSF, has been demonstrated and characterized by multi-angle laser light scattering, analytical ultracentrifugation, HDX-MS and SAXS. Experimental data using progesterone as a ligand and the native ~100 kDa ApoD tetramer from BCF, supports a particular tetramer conformation among those predicted by molecular modeling, where the binding pocket opening is accessible and the sugar moieties do not interfere in the inter-subunit interface (Figure 3H). Monomers interact with each other through the C-terminal α-helix and three β-sheets in close proximity, while glycosylated surfaces and Met93 are exposed in the tetramer. Oligomerization does not preclude ligand binding, and is not significantly altered upon binding of various ligands (biliverdin, palmitic acid, progesterone and sphingomyelin) or by *in vitro* protein oxidation with H_2_O_2_. The putative contribution of intermolecular disulfide bonds in the tetramer (involving human Cys116) has not been explored.

References contributing to this section are listed in **Reference Collection 4**, Supplementary Table 2.

#### Small Ligand Binding

The ability to bind progesterone was a defining feature of the most abundant protein in BCF, therefore named progesterone-binding cyst protein (PBCP) or gross cystic disease fluid protein 24 (GCDFP-24). Later on, this protein was demonstrated to be identical to ApoD purified from plasma HDL particles. Ligand-protein interaction at the ApoD binding pocket induces conformational changes leading to a more ordered structure, but does not result in major structural changes or altered oligomerization. These dynamic changes, though subtle, might have implications for ApoD interactions with other proteins or lipoprotein particles. Ligand binding reports are grouped in **Reference Collections 5, 6**, Supplementary Table 2.

Progesterone accommodation in the pocket involves a tryptophan residue heavily conserved in the Lipocalin family (Trp127 in human ApoD; Figures 2A, 3C), whose fluorescence (Ex. λ = 295 nm) changes upon binding. This element in the pocket makes Trp-fluorescence titration a valid method to test a variety of ligands for ApoD (Table 2). All *in vitro* ligand-binding experiments have been performed with the human protein, using either recombinant ApoD (expressed by bacteria or eukaryotic cells) or native protein purified from BCF or plasma HDL. Arachidonic acid (AA) shows the highest affinity, while various AA derivatives (e.g., prostaglandins, 12-HETE or 5,15-diHETE) show no binding by Trp-fluorescence titration.

**Table 2:** ApoD ligand binding *in vitro* assays.

**Ligand**	**Apparent Kd (μM)**	**Apparent Kd (μM)**	**Apparent Kd (μM)**	**Apparent Kd (μM)**	**Apparent Kd (μM)**
**References**	**Morais Cabral et al., 1995**	**Vogt and Skerra, 2001**	**Breustedt et al., 2006**	**Ruiz et al., 2013**	**García-Mateo et al., 2014**
All-*trans*-retinoic acid			2.8	4.0 ± 2.6	
Retinol			0.08 ± 0.04	0.2 ± 0.1	
Arachidonic acid	0.006 ± 0.004	3.2 ± 0.2			
2-Arachidonyl-glycerol				n.d.	
12-HETE	n.d.				
5,15-diHETE	n.d.				
Prostaglandins (D2, E1, F2a)	n.d.				
Lysophosphatidylcholine					1.13 ± 0.05
Linoleic acid	n.d.				
Oleic acid	n.d.				
Palmitic acid	n.d.			3.3 ± 0.6	
Palmitoyl sphingomyelin				1.3 ± 0.5	
Cholesterol	n.d.			n.d.	
pregnenolone		n.d.			
Progesterone	0.4 ± 0.1	1.7 ± 0.02			
Dihydrotestosterone		n.d.			
β-Estradiol				n.d.	
E-3M2H		n.d.			
Anandamide				1.6 ± 1.3	
Bilirubin	2.6 ± 0.5	n.d.			

*Ligands tested by tryptophan fluorescence-based assays in vitro. Apparent Kd (μM) average ± SD are shown. “n.d.” = no binding detected*.

Cholesterol, a reasonable candidate because of its high presence in plasma lipoprotein particles, has been repeatedly tested, and reported to have no binding, or a very low affinity one (Table 2). A series of works (**Reference Collection 6**, Supplementary Table 2) demonstrate that ApoD has no cholesterol-transfer activity, a hypothesis originated by ApoD co-purification with lecithin-cholesterol acyltransferase (LCAT), whose activity is in fact modulated by ApoD (see section Protein Physiology) by a mechanism discarding ApoD as a cholesterol provider for LCAT.

Only one ligand has been identified bound to ApoD and extracted from the protein after purification from a natural source. E-3-methyl-2-hexenoic acid (E-3M2H), a male axillary precursor of odorants, was identified by gas chromatography-mass spectrometry (GC/MS) after temperature/pH switch and chloroform extraction from purified ApoD.

Interestingly, various ligands (e.g., bilirubin or E-3M2H) whose interaction with ApoD has been demonstrated by a different technique, do not alter Trp-fluorescence, raising the possibility of other sites of interaction. Molecular dynamics simulations infer flexible binding of oxidized derivatives of AA (5s-, 12s-, and 15s-HpETE) around the conserved Met93 at one of the hydrophobic patches at the entrance of the pocket. This particular form of lipoperoxide binding to ApoD is not expected to produce changes in fluorescence of Trp-127, located at the bottom of the binding pocket. A proof of interaction is experimentally supported by site-directed mutagenesis combined with HPLC-detection of reduced lipids (HETEs) after exposure to ApoD. This interaction underlies the antioxidant activity of ApoD (see below). Figure 3I summarizes in cartoon form this new view of small ligand-binding sites of ApoD, not restricted to the Lipocalin pocket.

#### Protein-Protein Interactions

Interactions of ApoD to higher-order lipid structures, like lipoprotein particles or cellular membranes, are particularly relevant since they determine the range of sites and biological contexts where ApoD function can be performed. They might depend on protein-protein or protein-lipid contacts.

As mentioned above, co-purification of ApoD with LCAT might indicate the potential for a protein-protein interaction in nascent HDL particles, but a clear demonstration of ApoD-LCAT complex is not available. In contrast, a clear protein-protein interaction does account for human ApoD presence in HDL particles. An intermolecular disulfide link between ApoD Cys116 and ApoA-II Cys6 has been demonstrated by peptide digestion followed by sequencing and mass spectrometry (MS). This interaction, however, is an exclusive property of human ApoD due to its unique unpaired cysteine.

A putative disulfide-linked ApoD-ApoB100 complex was also proposed, but evidence is based on predictions from electrophoretic mobility in reducing/non-reducing conditions and immunoblot detection with anti-ApoD antibodies only, or with antibodies raised against LDL particles. An almost full characterization of 23 out of 25 cysteine residues in ApoB-100 by MS-analysis and peptide sequencing found no bonds with ApoD (Yang et al., 1990), strongly arguing against a disulfide-mediated interaction. Alternative mechanisms of ApoD interactions with plasma lipoprotein particles are therefore open to consideration.

Other potential interactions of ApoD have been explored with classic two-hybrid systems, where protein-protein contact takes place in the cell nuclei or cytoplasm, both requiring ectopic expression of ApoD in non-native biological compartments unsuitable for disulfide linked proteins (see sections Protein Structure and Cellular Trafficking). Alternatively, co-immunoprecipitation *in vitro* with or without crosslinking agents has been a method of choice. Using these approaches, ApoD has been proposed to interact with the extracellular glycoprotein Osteopontin (OPN), the intracellular domain of the Leptin Receptor (OB-Rb), the transmembrane glycoprotein Basigin (BSG), and the Scavenger receptor class B type 1 (SRB1).

The weak interaction reported between ApoD and the intracellular domain of OB-Rb, combined with its presumed topology within the cell, should discard this finding as a biologically relevant interaction for ApoD unless it is replicated. For membrane proteins such as BSG and SRB1, proposed as putative membrane receptors for ApoD, co-localization by confocal imaging is often used as additional evidence. However, protein-complexes are below the resolution of standard co-labeling techniques, and methods relying on distance-dependent energy transfer, super-resolution or immunoelectron microscopy would be desirable as further evidence in relevant *in vivo* conditions. Other candidate ApoD receptors (LDLR and CXCR-4) are predicted from physiological contexts, where downstream consequences of ApoD exposure are modified by antagonists of these receptors. However, a direct interaction with these receptors has not been explored.

References contributing to this section are listed in **Reference Collection 7**, Supplementary Table 2.

#### Binding to Lipid-Rich Structures

The presence of ApoD in plasma lipoprotein particles lies at the base of its discovery in humans. ApoD was initially visualized as a “thin-line” polypeptide in immune-double diffusion analyses of plasma HDL particles, and was then identified as a low-abundance component of HDL_3_ particles (defined as small-dense HDLs, d = 1.12–1.27 g/ml). Analysis of HDLs separated by electrophoretic mobility in non-denaturing PAGE followed by in-gel trypsinization, identified ApoD within the HDL-α2 type, in a 1:100 ratio with respect to ApoA-I. The presence of ApoD in HDLs has been confirmed also in human CSF and in baboon and mouse plasma. Additionally, plasma ApoB-100 positive LDL particles contain ApoD as well, but at lower concentrations (~8 ng ApoD/μg LDL vs. ~69 ng ApoD/μg HDL_3_). The generalized interaction with different lipoparticles in several species suggests that ApoD-lipoparticle interactions must rely on a mechanism independent of ApoD-ApoA-II disulfide bond, a human HDL rarity. The fact that ApoD-LDL interaction is prevented by detergents, and do not take place with recombinant ApoD where hydrophobic surface residues have been mutated (to favor crystallization), suggests a hydrophobicity-dependent ApoD-lipoparticle binding mechanism (Figure 3J).

Also, direct binding of ApoD to unilamellar phospholipid vesicles (liposomes) further demonstrates its ability to bind to lipidic structures without requiring a protein-protein interaction. These unilamellar vesicles represent a simplified version of the outer phospholipid layer of HDLs, LDLs or a membrane bilayer (Figure 3K). In addition, ApoD has recently been identified in extracellular vesicles, characterized by the presence of CD81, CD63, and flotillin-1, and a density of d = 1.17–1.23 g/ml. The hydrophobic patches of ApoD at the entrance of the binding pocket are the likely site of interaction with liposomes or biological membranes, as indicated by experiments combining ApoD capacity to reduce oxidized liposomes with mutagenesis of Met residues that in fact contribute to the hydrophobicity of those patches.

The knowledge accrued on ApoD protein structure, its glycosylation and oligomerization properties, as well as its interactions with small ligands and other lipidic structures are relevant for its physiological roles in lipid management, and should help to get a global picture of how these molecular properties are put to work in various physiological contexts.

References contributing to this section are listed in **Reference Collection 8**, Supplementary Table 2.

### Gene Data and Genomic Properties

#### Chromosomal Position and Gene Structure

The gene coding for ApoD locates in an autosomic chromosome that shows ample synteny in chordates (Sanchez et al., 2006), reflecting a strong evolutionary conservation of this genomic region (Figure 2B). The ApoD gene shows a standard metazoan exon-intron architecture, with a coding sequence interspersed in four exons that is conserved in chordates (Sánchez et al., 2003). Moreover, the gene upstream and downstream untranslated regions (UTRs) are also composed of several exons, mainly in the 5′-UTR, a property well-preserved in mammals (Mejias et al., 2019) (Figure 2C).

References contributing to this section are listed in **Reference Collection 9**, Supplementary Table 3.

#### Transcriptional Control of Gene Expression

The promoter region and elements controlling the expression of ApoD have being studied in detail for the human gene. The human promoter shows a canonic TATA-box upstream of the transcription start site. Several promoter elements and nuclear factors have been predicted to potentially regulate ApoD transcription in a number of organisms.

Experimental proof of a regulatory potential of human ApoD has been gathered for SRE1, AP-1, APR-3, NFκB, PARP1, HnRNP-U, and APEX-1 in cultured cells subjected to inflammation (LPS) and metabolic stress (serum deprivation). Also, the transactivator TAp73 mediates ApoD expression upon cell differentiation. The mouse ApoD promoter region has been recently assessed experimentally, and an alternative promoter region has been related to OS-induced ApoD expression.

DNA methylation, inferred from the CpG content of the gene promoter region, is also an important regulatory mechanism for ApoD transcription, with an inverse relationship between level of DNA methylation and ApoD gene transcription. This gene regulation mechanism has been shown in different physiological or pathological contexts: in esophageal, colorectal and astrocytic cancers, in the expression profile defining Th17 lymphocytes, and for the androgen receptor-response in male sexual development.

References contributing to this section are listed in **Reference Collection 10**, Supplementary Table 3.

#### Post-transcriptional and Translational Control of Gene Expression

The mRNA 3′-UTR is known to influence its stability and translation efficiency. ApoD 3′-UTRs show a high degree of conservation in mammals, and display shorter lengths and higher G+C content than those observed in average mammalian gene UTRs. These differences have been proposed to underlie a tight regulatory control of ApoD translation. In this context, a number of miRNAs have been predicted to control ApoD translation, possibly by binding to the 3′-UTR. Some of these miRNAs, like miR-229b-3p, miR-423-3p, and miR-490-3p, have been experimentally tested and implicated in the post-transcriptional downregulation of ApoD expression in rat male reproductive system upon metabolic dysfunction.

The 5′-UTR of ApoD also presents relevant properties for the regulation of ApoD expression. It is rich in short-tandem repeats (STR), specifically in primates. Long stretches of STRs are predicted to affect transcription and translation, which might have contributed to the neurodevelopmental changes that underlie primate evolution. Furthermore, mammalian ApoD genes show several alternative 5′-UTRs forms, possibly arising from alternative splicing. The alternative 5′-UTRs of the mouse ApoD gene have been experimentally tested and shown to underlie differential protein expression in several mouse tissues, with a particular 5′-UTR variant being strongly induced upon OS. Moreover, *in silico* analyses of these 5′-UTR variants in mouse and human ApoD show upstream initiation codons, upstream open reading frames, and predicted secondary structures that suggest a tight control on ApoD gene expression.

References contributing to this section are listed in **Reference Collection 11**, Supplementary Table 3.

#### Gene Polymorphisms

In terms of genetic variation for the ApoD gene, over 4,600 variants have been found in the GRCH38.p12 (annotation Release 109) assembly of the human genome, while 187 are reported in the short variants (dbSNP) and structural variants (dbVar) databases. Six variants that involve missense, intron insertions and 3′-UTR insertions, are predicted to involve molecular consequences. Some of these variants have been linked with variable support to human cancer, metabolic or neurological diseases (see Supplementary Table 18, and section ApoD-Disease Relationships), but a final proof of their clinical significance is currently missing.

References contributing to this section are listed in **Reference Collection 12**, Supplementary Table 3.

### Regulation of Expression

**Figure 4:**
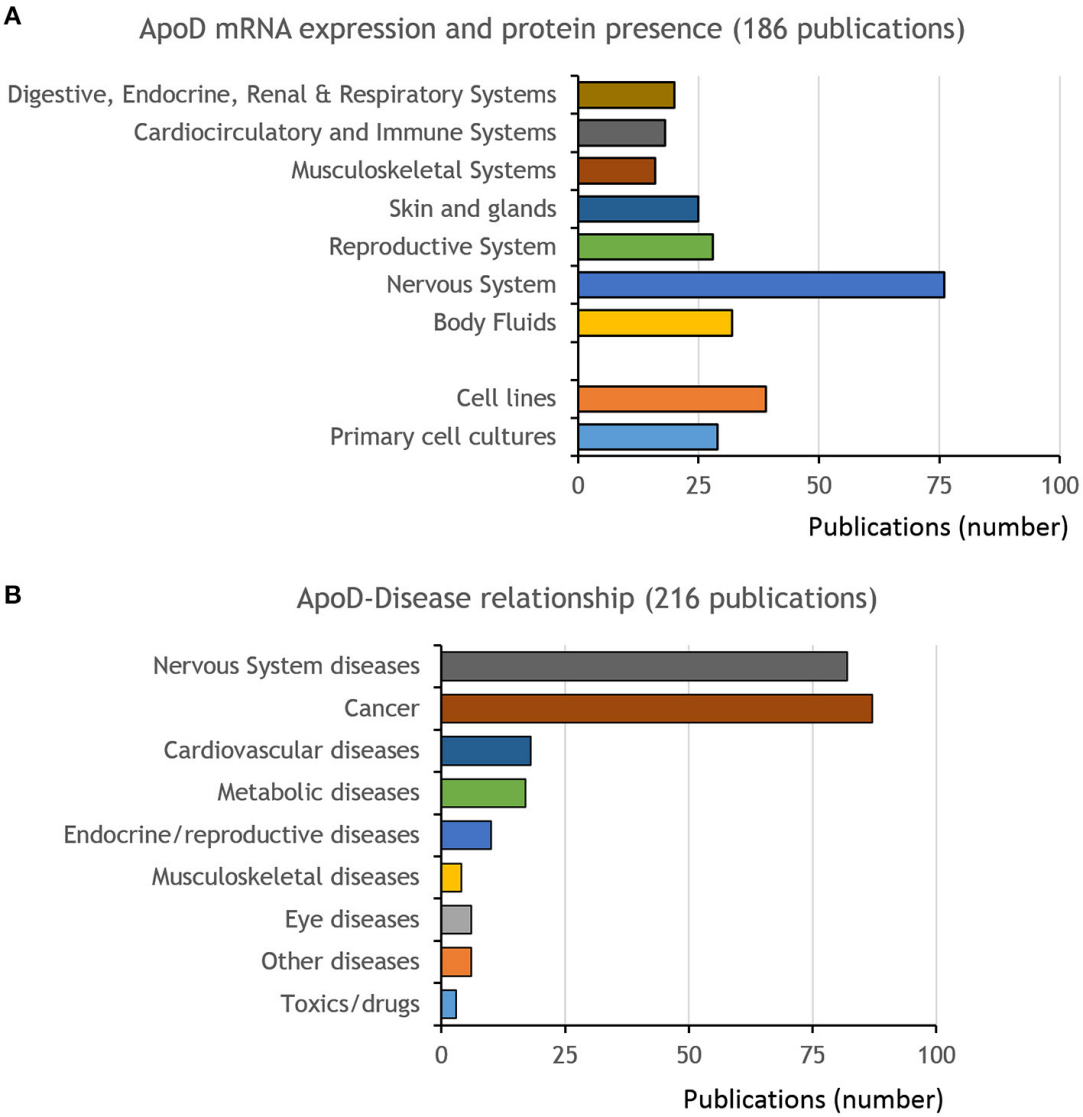
Publications on ApoD expression and disease relationships. **(A)** Distribution of publications describing ApoD mRNA expression or protein presence *in vivo*, distributed by physiological systems and in cell cultures (primary cells or cell lines). **(B)** Publications with information on ApoD relationship to disease (expression changes triggered by disease or treatments, or association of ApoD gene variants with disease).

A total of 186 primary publications (Figure 4A) were labeled with the *regulation of expression* (RE) tag for this systematic review (details recorded in Supplementary Tables 9–17). We combined our analysis with current data compiled in human and mouse expression atlases (see Methods section; Supplementary Figures 1, 2).

#### ApoD in Body Fluids

Since its discovery in plasma HDL particles, ApoD protein and/or mRNA have been found in almost every organ, tissue or fluid. In addition to plasma, ApoD protein is present CSF, perilymph, urine, and secretions from exocrine glands (sweat, tears and mammary secretions) (Supplementary Table 9). The cellular origin of ApoD protein in each of these body fluids is not fully elucidated. With the exception of Th17 lymphocytes, blood cells in general do not express ApoD mRNA, and liver and intestine (major sites of HDL biogenesis) are among the ApoD low-expressing tissues both in humans and mice (Supplementary Figures 1, 2). Plasma ApoD protein (~128 mg/l) is approximately 25 times the concentration of CSF ApoD (~5 mg/l) in healthy adult men, and they are uncorrelated, suggesting that a separate pool of ApoD protein is managed in these barrier-separated compartments.

Avian egg fluids are also rich in ApoD, with the interesting property that egg white ApoD positively correlates with egg freshness.

References contributing to this section are listed in **Reference Collection 13**, Supplementary Table 4.

#### Tissue and Cellular Expression Patterns and Response to Stimuli

The analysis of tissue expression pattern leads to a general conclusion: in spite of its wide distribution, ApoD is never ubiquitously expressed, never in all cell types in a tissue, or at all times in a given cell type. ApoD is expressed in most tissues with a salt-and-pepper spatiotemporal pattern, suggesting a fine control that depends on particular physiological cell states. Furthermore, all tissues bear ApoD-expressing cells and cells able to endocytose ApoD protein from the extracellular milieu (see section Cellular Trafficking). These expression features, along with ApoD being a very stable protein, result in a high protein abundance when measured in high-throughput analyses, and in a lack of exact fit between mRNA and protein expression in a given tissue or cell (Supplementary Figure 1; **Reference Collections 14–16**; Supplementary Table 4). While tissues as the female breast present high levels of ApoD mRNA and protein, organs like the liver show high abundance of ApoD protein, but barely detectable ApoD mRNA both in human and mice. At the other end of the spectrum, blood cells and immune system-related organs are among those with low levels or no expression of ApoD, either mRNA or protein.

Organs and tissues involved in both male and female reproductive physiology express ApoD (Supplementary Table 10). The high expression of ApoD in breast has been located to the glandular epithelium (Supplementary Figure 1), and breast cysts accumulate high amounts of ApoD protein, making BCF a useful experimental source of native ApoD protein. ApoD mRNA is detected at all stages of the spermatogenesis process in testis and in ovarian theca cells. Along the female cycle, stromal and epithelial cells of the endometrium express ApoD mRNA and protein during the secretory phase. ApoD is also expressed during corpus luteum maturation in the ovary. Gestation alters ApoD abundance in plasma as well (Supplementary Table 9), with a decrease during a healthy pregnancy followed by a fast recovery if the mother breastfeeds her baby. ApoD expression in breast secretions and skin is also altered upon establishment of menopause.

**Figure 5:**
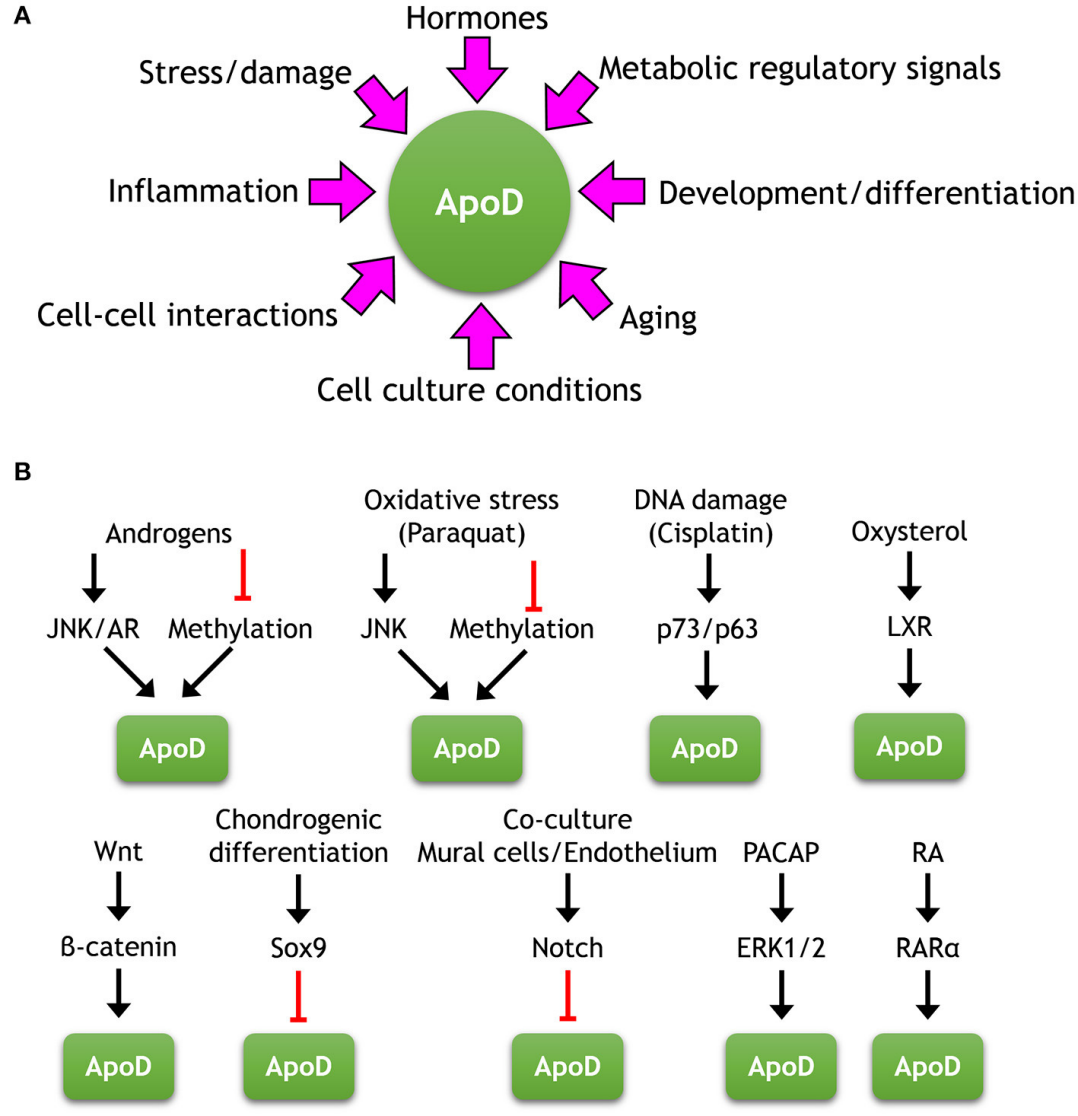
Factors and pathways regulating the expression of ApoD. **(A)** Diverse stimuli regulate ApoD expression in a variety of cells and physiological conditions. **(B)** Summary of upstream regulatory pathways regulating ApoD expression where elements of the signaling cascade have been identified.

These temporal patterns of expression are due to hormone regulation, as demonstrated by both *in vivo* and *in vitro* studies (Supplementary Tables 10, 16, 17; **Reference Collection 17**). Upregulation of ApoD by androgens is well documented in different preparations like breast explants, male genital fibroblasts or primary epithelial cells from male axillary apocrine glands. This regulation is mediated by nuclear androgen receptor (AR), and ApoD is being used as an AR activity assay (Figure 5B). Estrogens and progesterone, alone or in combination, also change ApoD expression in several experimental settings, with more variation in the final outcome depending on cell type (e.g., breast cancer cell lines up-regulate ApoD upon exposure to 17β-estradiol, while prostate cancer cell lines down-regulate it, Supplementary Table 17). Sex hormone-regulation of ApoD is also present in birds, in the context of oviposition cycles or egg fertilization, thus representing relevant biological stimuli for ApoD spatiotemporal regulation throughout evolution (Figure 5, Supplementary Table 10).

Expression in the nervous system (Supplementary Table 11) has been amply explored for ApoD, with primary publications doubling those devoted to other tissues or systems (Figure 4). All evidences support a prominent and consistent ApoD expression in the nervous system, where myelinating glial cells (oligodendrocytes and Schwann cells) constitute the main sites of expression in control conditions, followed by a more disseminated expression in astrocytes. ApoD protein abundance in the nervous system is accounted for by the fact that ApoD associates to myelin itself, a structure representing a large proportion of the vertebrate nervous system volume. As mentioned above, only subsets of cells express ApoD at a given time or location for each cell type (**Reference Collection 16**, Supplementary Table 4).

In addition to glial cells, ApoD has been found in meninges and the vascular system of the nervous system (Supplementary Table 11), particularly in pial and perivascular cells (mural cells or pericytes) associated to the capillary beds. During mouse embryogenesis, ApoD has been detected in both pericytes and endothelial cells, and mRNA expression in the latter is under the control of Wnt/β-catenin signaling during the time interval of blood-brain-barrier formation (Figure 5B). ApoD-positive pericytes and other perivascular cells are also reported in the adult nervous system. RNAseq analyses of acutely isolated cortical cells show endothelial cells as second to myelinating oligodendrocytes in ApoD enrichment.

Although neuronal expression has been subject to debate (Supplementary Table 11), unambiguous detection of ApoD mRNA in neurons has been reported only in the developing brain. In contrast, detection of ApoD protein in some neurons has been reported at various ages in healthy control situations, while this finding is more abundant upon aging or disease. Neuronal uptake of ApoD upon disease has a certain degree of specificity. It is frequently found in the brain of Alzheimer's, but not in Parkinson's disease patients. Animal models of brain traumatic injury, stroke and Niemann-Pick type A disease do show neurons that have internalized ApoD protein, while they are not found in the Niemann-Pick type C mouse model. Transfer of ApoD from astroglial cells to neurons has been demonstrated in cell culture preparations, and shown to be mediated by extracellular vesicles (Supplementary Table 17; see section Cellular Trafficking).

A well-established fact with strong support from different studies is that ApoD expression increases throughout brain aging (**Reference Collection 18**, Supplementary Tables 4, 11), a pattern conserved in several species analyzed with just one exception: a study documenting a decrease in ApoD mRNA in the aging avian hippocampus. A higher ApoD expression in cortex and brainstem in comparison with hippocampus or cerebellum are well-supported regional differences within the brain (Supplementary Table 11). In the highly-expressing prefrontal cortex, the increase of ApoD mRNA and protein throughout life positively correlates with proteins involved in antioxidant defense.

The expression data obtained from healthy individuals is coherent with an ApoD gene response to diverse experimental stress or injury paradigms (**Reference Collections 19–20**, Supplementary Table 4) that include oxidative stress (OS), peripheral nerve or traumatic brain injury, kainate excitotoxicity, damage by middle cerebral artery occlusion or by viral infection and experimental inflammation. All of the above results in increased ApoD expression *in vivo*. This ApoD stress response is mostly, but not exclusively, documented in the nervous system (e.g., OS-triggered upregulation is also observed in the cardiovascular system). These patterns of response can be extended to the many disease situations reviewed in section ApoD-Disease Relationships. In addition to the abundant correlative data from human diseases, experiments in animal models of disease analyzed *in vivo*, primary cell cultures and cell lines support a major conclusion: ApoD is a key player in the endogenous response to a variety of potentially harmful stimuli. The damage and stress responsive p73/p63 and JNK pathways have been demonstrated to up-regulate ApoD (Supplementary Tables 16, 17 and Figure 5), while the particular signaling cascades regulating ApoD upon other stress or inflammation inducers (e.g., H_2_O_2_, UV light or LPS) remains to be elucidated. Not all stressful conditions trigger ApoD expression (Supplementary Table 17), underscoring the specificity of pathways regulating ApoD (Figure 5). Moreover, a fine regulation of ApoD upon OS seems necessary, since it involves various non-exclusive mechanisms like DNA demethylation, the use of alternative promoters or 5′-UTR specific mRNA variants (see section Gene Data and Genomic Properties).

Nutritional and metabolic states also regulate ApoD expression (**Reference Collection 21**, Supplementary Table 4), and ApoD upregulation under caloric restriction or ADCY5 loss-of-function seems to be part of a common signature leading to lifespan extension. Curiously, these results derived from *in vivo* studies agree with ApoD upregulation upon serum starvation in cell culture systems (**Reference Collection 22**, Supplementary Table 4). New studies on how metabolic switches can modulate ApoD in different contexts, and searching for the specific signaling pathways that trigger ApoD expression are therefore valuable. A particular lipid-managing pathway is known to control ApoD expression: ApoD is a target gene for LXR in liver, skeletal muscle, adipocytes and endothelial cells, thus becoming part of the response to oxysterol stimulation.

Pathways involved in development and cell differentiation are also known to regulate ApoD expression (**Reference Collection 23**; Supplementary Tables 4, 16, 17). In addition to its regulation by the Wnt/β-catenin pathway mentioned above, ApoD is downstream of Sox9 during chondrogenic differentiation, and of PACAP/Erk signaling during adipocyte differentiation. Also, particular cell-cell interactions regulate ApoD expression in one of the cellular partners, like endothelial-mural cell interactions relevant during the angiogenesis process. In this scenario, ApoD is downregulated in mural cells by contact-dependent (Notch-3) and contact-independent mechanisms.

Finally, confluency and senescence in cell cultures also trigger ApoD expression (**Reference Collection 22**, Supplementary Table 4). These culture conditions parallel steady-state situations of cells in their physiological tissue environment and the *in vivo* upregulation by aging, respectively. Both conditions concur with a halt in cell division, as it is also the case for serum starvation conditions. The good prognosis of some types of cancers where ApoD increases, also relates its expression to low cell-division rate (see section ApoD-Disease Relationships). Retinoic acid induction of ApoD expression, mediated specifically by RARα in breast cancer cells, correlates with the anti-proliferative action of this signaling pathway. However, the potential role of ApoD in regulating cell division (see section Protein Physiology) must be dependent on the physiological/pathological context. For example, in the model of pericyte-endothelium interactions mentioned above, mural cells decrease ApoD expression upon interaction with endothelial cells, when they would stop dividing to generate mature capillary structures.

Figure 5 summarizes stimuli regulating ApoD expression and the particular upstream signaling pathways known to date.

### ApoD-Disease Relationships

The reports in this section either study the expression of ApoD in response to disease and therapies, or evaluate association of ApoD gene variants with disease. Information was accrued from a total of 216 primary publications (Figure 4B; details in Supplementary Table 18).

That ApoD is part to the endogenous response to a wide range of diseases, with diverse primary causes, is uncontentious. Data support the existence of common factors underlying diverse disease situations that cause ApoD expression changes, and OS is the strongest candidate. The ApoD upregulation upon experimental stress or injury reviewed above is coherent with prominent examples concurring in the nervous system (**Reference Collection 27**, Supplementary Table 5), where 85% of 66 reports on degenerative/psychiatric diseases or naturally occurring injury identify an over-expression of ApoD. Exceptions are the down-regulation observed in neurotransmission-centered diseases, like depression and a DOPA-decarboxylase deficiency.

Cancer is the other major disease where changes in ApoD expression have been analyzed (82 reports). A clear negative correlation between ApoD expression and malignancy has been found in nervous system tumors, fibrosarcomas, breast, colorectal, hepatic, renal and cervical cancers. The general association of a good prognosis with high ApoD expression strongly suggests a protective anti-tumoral function for this Lipocalin. While studies of prostate cancer have not evidenced unambiguously such a pattern, some studies show regional ApoD expression differences (high in juxta-tumoral tissue) that are still compatible with a defensive tissue response to neoplastic transformation. ApoD tumor-suppressing activity has been experimentally tested and an inverse relationship between ApoD promoter methylation, ApoD expression and outcome is supported by various reports (Supplementary Table 18). Whether a common mechanism of ApoD function can promote survival of damaged postmitotic cells in neurodegenerative diseases, and also prevents proliferation of cancerous cells deserves further analysis.

Cardiovascular and metabolic diseases (particularly diabetes) as well as infection or injury, are also accompanied by ApoD upregulation. Again, OS might be a common link to ApoD response to these diseases, for instance in atherosclerotic plaques depending on disease progression, or upon oxidative degradation of glycated proteins in diabetes.

In contrast to the many diseases where ApoD expression changes have been reported, few genetic variations of ApoD have been widely or robustly linked to disease risk or prognosis (see section Gene Polymorphisms and Supplementary Table 18). Among the few cases reported, it is striking that most of them occur in non-coding sequences (introns or UTRs) revealing that pathogenic variations in ApoD protein sequence must be too deleterious to survive in extant populations.

### Cellular Trafficking

**Figure 6:**
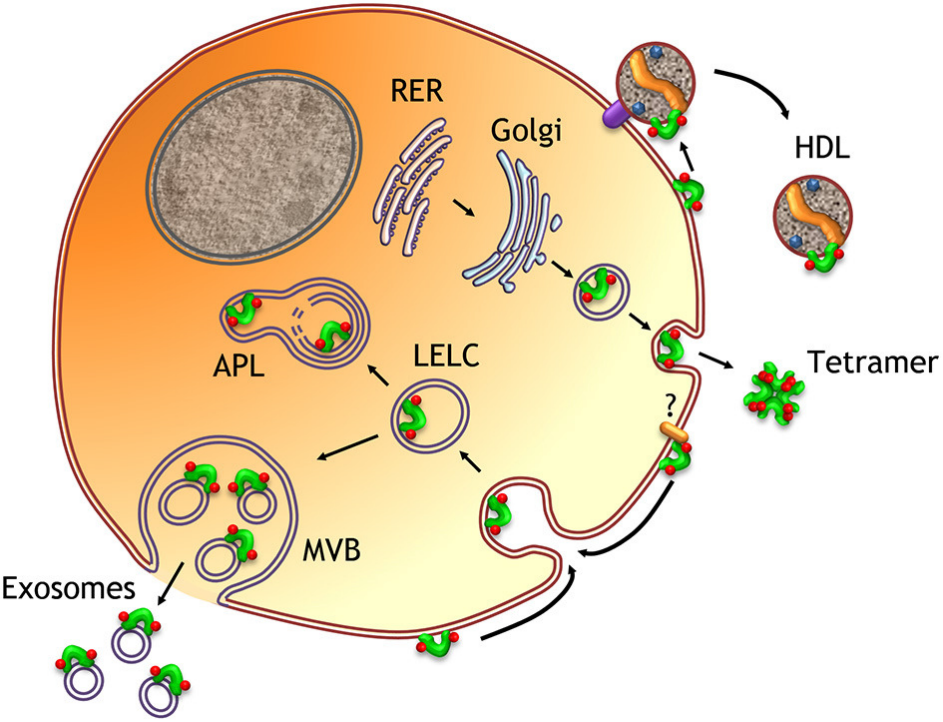
Schematic representation of ApoD subcellular traffic. A model of an ApoD-expressing cell is represented. Canonical exocytosis through the RER-Golgi pathway generates the mature, glycosylated (red dots) protein. The tetrameric form identified in the breast cyst fluid is represented as the format detected in extracellular fluids. Once at the plasma membrane, ApoD can be endocytosed (by non-expressing cells as well) and targeted to lysosomes and autophagolysosomes. When endolysosomes develop into multivesicular bodies, ApoD would be carried on the outer surface of exosomes. Finally, ApoD can be transferred to HDL during their biogenesis, or during their lipid-efflux activity (upon HDL-receptor interaction).

The consistent finding of ApoD in body fluids and the signal sequence present in the translated polypeptide indicate that ApoD is exocytosed from cells expressing the protein. A consistent set of experimental work supports the association of ApoD to the rough endoplasmic reticulum (ER), the signal peptide removal in the protein sorting process, the N-linked oligosaccharide modification carried out in RER-Golgi, and a secretion of the mature glycoprotein to the extracellular environment in several tissues and cultured cells. All these data make ApoD a typical soluble extracellular protein undergoing a canonical secretory pathway, a consensus attained by subcellular localization prediction algorithms and data present in gene ontology databases (see Methods). Additionally, subcellular traffic of ApoD can also lead to its exportation out of the cell in different formats that include ApoD tetramers, HDL-associated ApoD and extracellular vesicle-associated ApoD (Figure 6).

However, several reports have interpreted their findings about ApoD biological roles on the basis of protein partitioning in cytoplasm and/or nuclear compartments. Aside of technical issues questioning those results, some reports use overexpression of fusion-tagged proteins, which are known to undergo unnatural compartmentalization or degradation. Also, strategies based on *in vitro* interaction assays that were designed for proteins naturally occurring in the cytoplasmic or nuclear compartments (like the classic two-hybrid assays) preclude the finding of functionally relevant interactions for ApoD. An alleged cytosolic ApoD would likely be non-glycosylated and improperly folded in the absence of its intramolecular disulfide bonds.

#### Intracellular Traffic

A number of studies have shown the presence of ApoD in RER and vesicular compartments of different eukaryotic cells. Immunoelectron microscopy (EM) of nervous system cell types has unambiguously identified ApoD in the outer nuclear membrane-RER membrane complex and in lysosomes. Lysosomes isolated from placental cells, monitored with biochemical techniques, contain ApoD. Likewise, clathrin-coated vesicles isolated from hen's ovaries also contain ApoD.

Recently, the subcellular location of ApoD has been studied in detail in basal conditions and under experimental stimuli, either by immunogold-EM microscopy, or by fluorescence immunocytochemistry combined with established cell compartment markers and monitored by well-documented standardized confocal microscopy. These studies have detected the presence of ApoD in RER, the Golgi apparatus, endosomes, lysosomes, autophagosomes, multivesicular bodies, as well as in the outer side of plasma membrane, clathrin-coated vesicles and caveolae. The protein does not localize to mitochondria or peroxisomes, and has not been immunolocalized inside cell nuclei. Lysosomal ApoD localization has been demonstrated in astrocytes, oligodendrocytes, Schwann cells, fibroblasts, and neurons.

Although those experiments were performed at a fixed time point, serial-time experiments demonstrated that the presence of ApoD in the endosome-lysosome compartment is stable and dynamically enriched upon OS exposure. Long-lasting lysosomal location of ApoD depends on its glycosylation and hydrophobicity, as mutated bacterial recombinant ApoD is maintained in lysosomes only transiently. Targeting of ApoD to the lysosomal compartment occurs not only in ApoD-expressing cells, but also in non-expressing neurons upon exposure to exogenous ApoD or when co-cultured with astrocytes. The fact that ApoD is a stable component of subsets of lysosomes at a given time, connects many of the apparently diverse physiological roles of ApoD (see section Protein Physiology).

References contributing to this section are listed in **Reference Collection 33**, Supplementary Table 6.

#### Exocytosis

It is undoubtedly established, as discussed above, that ApoD is being secreted to the extracellular environment following a canonical secretory pathway (Figure 6). However, whether this pathway leads to actual secretion of ApoD in monomeric form has not been established. In addition, extracellular vesicles (EVs) constitute an alternative exocytotic path for ApoD (see section Binding to Lipid-Rich Structures). Proteomic analyses have identified ApoD in EVs from human plasma and CSF. Experimental characterization of EVs produced by a human astroglial cell line and by mouse primary astrocytes, identified the ApoD-positive vesicles as exosomes originated from multivesicular bodies, according to their size (~100 nm), density (1.17–1.23 g/ml) and molecular markers. When exported by glial cells in exosomes, ApoD must be located on the external surface of these EVs (Figure 6).

A third mechanism by which ApoD becomes extracellular is by traveling in HDL particles. ApoD-HDL association can take place during HDL biogenesis, or ApoD can associate to HDLs while the lipoparticles bind to cell membranes and perform their lipid efflux activity. However, these mechanistic details and the particular subcellular origin of the HDL-associated ApoD detected in body fluids need to be investigated. The plasma membrane location of both ApoA-I dependent HDL biogenesis (Denis et al., 2008) and ABCA1-dependent cholesterol efflux activity (Phillips, 2018), makes it a likely location for the origin of ApoD-positive HDL particles (Figure 6).

References contributing to this section are listed in **Reference Collection 34**, Supplementary Table 6.

#### Endocytosis

The immunolocalization of ApoD in cells not expressing the gene (see section Tissue and Cellular Expression Patterns and Response to Stimuli), as well as the internalization of ApoD by cells cultured in the presence of its native or recombinant forms, are the experimental basis supporting the endocytosis of this Lipocalin. It takes place both under control conditions and in response to specific biological stimuli. ApoD endocytosis appears as a general property of this protein, as it has been reported in birds and mammals. Particularly, in glia-neuron co-cultures ApoD is found to be exclusively transported in EVs from astrocytes to neurons, where it gets internalized. The current view of several extracellular formats of ApoD (HDL, EVs or tetramers in solution) makes it worth to study whether different membrane interaction mechanisms or endocytosis paths are used for ApoD internalization.

ApoD association to the extracellular side of the plasma membrane is coherent with both, its traffic from RER to plasma membrane by the canonical exocytotic path and with its cell contact before internalization. ApoD-plasma membrane interaction has been experimentally demonstrated and is currently considered an established localization for ApoD in human cells (https://www.proteinatlas.org/ENSG00000189058-APOD/cell). Whether ApoD-membrane association is mediated by protein-protein or protein-lipid interactions requires further research (see sections Protein-Protein Interactions and Binding to Lipid-Rich Structures). Figure 6 summarizes ApoD intra and extracellular traffic as currently known.

References contributing to this section are listed in **Reference Collection 35**, Supplementary Table 6.

### Tissue and Organ Function

The reports tagged in this section were selected because they study the function of ApoD by experimentally altering ApoD natural expression levels, or by subjecting cells or tissues to defined concentrations of the protein in a controlled experimental situation. A critical review of these reports aims at uncovering common and distinct roles for ApoD in different physiological organ and cellular systems.

#### ApoD Functions in Cardiovascular System

The process of angiogenesis has been a focus of interest to study the role of ApoD, given its reported expression by blood vessel mural cells (MCs: smooth muscle cells and pericytes). Both in embryonic development and during the remodeling process of wound healing, ApoD increased expression is causally linked to undifferentiated mural cells migration, though is not consistently related to cell proliferation (as it is often found in cancer cells; see section ApoD-Disease Relationships). A crosstalk between endothelial cells (ECs) and MCs governs the switch of the angiogenic cellular process from a proliferative/migratory state to a differentiation state, characterized by quiescence and adhesion. This switch is essential for vessel morphogenesis. Blood vessel angiogenesis involves reactive oxygen species (ROS), EC-derived PDGF-BB, JAGGED1 and NO, as well as MC-expressed NOTCH3. These secreted and cell contact-mediated signaling downregulates ApoD in MCs, a process linked to blood vessel maturation. Experimental manipulation of ApoD levels demonstrates that ApoD regulates the adhesion of MCs to the extracellular matrix, and low levels of ApoD promote Zyxin- and Vinculin-positive focal adhesion contacts. Concordant effects have been reported in retinal choroid vessels: ApoD-KO mice show neovascularization with increased permeability.

Myocardial cells show slight expression of ApoD under normal circumstances, but the tissue surrounding an injured/infarcted area promptly upregulates ApoD. The study of this process in ApoD-KO mice indicates that ApoD is associated to protection from cell death in the injured tissue. This ApoD protective role on cardiomyocytes is dependent on a proper protein fold and strongly correlates with its antioxidant activity (see sections Binding to Lipid-Rich Structures and Protein Physiology). In this experimental paradigm, the protective activity is attained by increasing ApoD in plasma, though it is unclear whether ApoD levels are also elevated in the infarcted tissue. We thus propose that ApoD function in myocardial tissue protection and remodeling might be based on: (1) A modulation of cell viability in cardiomyocytes and vessel ECs, possibly due to internalization of plasma-derived ApoD, and/or (2) a regulation of cell differentiation related to the angiogenic response described above, organized by ECs and MCs.

References contributing to this section are listed in **Reference Collection 36**, Supplementary Table 7.

#### Roles of ApoD in Metabolism Regulation

The role of ApoD in metabolism has been analyzed *in vivo* by using two different ApoD-KO mouse lines and a transgenic mouse (hApoD-Tg) driving the expression of human ApoD under the control of the human THY1 gene. This hApoD-Tg mouse ectopically expresses hApoD mostly in neurons, but the protein is present in plasma and other organs physiologically relevant to metabolism. Also, adenovirus-driven liver production of mouse ApoD has been used as a paradigm of acute overexpression, leading to elevated protein levels in plasma.

The metabolic consequences of altering ApoD levels have been evaluated mostly in plasma and liver, although also in retina, and measured in a variety of experimental settings: fasting or non-fasting conditions, different feeding diets, and different sex or age of animals. No clear pattern can be extracted for the role of ApoD on carbohydrate metabolism, where reports describe varied outcomes on glucose tolerance or insulin resistance depending on experimental conditions. Some consistency is observed in the effects on triglycerides (TG): Loss of ApoD leads to decreased hepatic TG content and increased plasma TG, while overexpression leads to elevated TG levels in liver and unaltered or decreased triglyceridemia (depending on the strategy used for ApoD overexpression). On the other hand, variations in plasma cholesterol levels are also reported, with various outcomes upon ApoD loss or overexpression. Also, association of ApoD polymorphisms have been found with both increased and decreased HDL-cholesterol species. The finding of ApoD being able to mediate binding of HDL to LDL, and of HDL particles to actively dividing carcinoma cells, suggests that it can regulate lipid traffic indirectly by influencing lipoparticle dynamics. Variations in local physiological contexts of this traffic mechanism might contribute in very different ways to the final systemic outputs measured in the experimental settings studied *in vivo*. However, more work is needed to derive definitive evidence for understanding the role of ApoD in lipid and carbohydrate metabolism. So far, the relevant results indicate that the functional relationship of ApoD with various metabolic parameters is, at most, indirect and dependent on other physiological conditions.

References contributing to this section are listed in **Reference Collection 37**, Supplementary Table 7.

#### ApoD Functions in Skeletal System

Bone cells, from bone marrow stem cells (MSCs) to osteoblasts, are reported to express ApoD in cell culture systems (Supplementary Tables 16, 17), and two reports have focused on testing the effects of experimental manipulations of ApoD levels on bone formation and remodeling. Relevant sex and hormone-related patterns have been found using ApoD-KO or hApoD-Tg mice and cell culture systems. With both approaches ApoD appears as an osteogenic factor. Lack of ApoD in mice reduces bone volume and thickness. These effects are observed in trabecular and cortical bone in females, but only in cortical bone in males. Enhanced bone turnover in female ApoD-KO mice is indicated by increased osteoblast surface and osteoclast numbers. Primary MSCs from ApoD-KO mice have lower survival and proliferation, and increased osteoclastogenesis, but an uptake of exogenous hApoD partially reverts their osteogenic potential. When osteoporosis is modeled by glucocorticoid (dexamethasone) treatment after osteogenic induction of MSCs, overexpression of ApoD reverts the effects of dexamethasone, as measured by PI3K/Akt pathway activity and downstream osteogenic gene expression, thus promoting the osteogenic process. Osteogenesis is accompanied by SOD and catalase upregulation, and oxidative damage is associated with glucocorticoid-induced osteoporosis, thus linking ApoD function in this context to its antioxidant activity.

References contributing to this section are listed in **Reference Collection 38**, Supplementary Table 7.

#### ApoD Functions in the Nervous System

As presented above, the current evidence supports a general view in which non-neuronal cells become the source of ApoD in response to different stimuli, and neurons count on the Lipocalin for its cellular functions by internalizing ApoD. Neurotransmission is one of those functions modulated by ApoD. Analysis of downstream effects in gene expression in the brain of ApoD-KO or hApoD-Tg mice, reveal an enrichment of genes related to synaptic transmission. Particularly, changes in glutamate, somatostatin, dopamine and acetylcholine neurotransmission have been independently documented by receptor binding assays, HPLC determination of neurotransmitters or their catabolites, or receptor immunodetection. These effects might underlie the behavioral phenotypes related to locomotor function, motor and spatial learning, and retinal function observed in mice with altered expression levels of ApoD.

ApoD addition to cultured neurons results in neuritogenesis and synaptogenesis, which are crucial during neuronal development and underlie neuronal plasticity of established circuits. Neuritogenesis is promoted in immature neurons in culture by the combined addition of ApoD and retinoic acid in the absence of serum. Experiments combining ApoD addition with receptor antagonists indicate that ApoD-dependent neurite extension can be mediated by LDLR, and possibly also by CXCR4 activation, pathways known to be involved in neuronal differentiation.

Another general function of ApoD, extensively analyzed in loss-of-function and transgenic mice, is its role in the glial response to dyshomeostatic changes in the nervous system due to oxidative, metabolic or traumatic stresses. Many studies have reported an acute regulation of ApoD expression under these insults, either experimental or triggered by disease (see sections Regulation of Expression and ApoD-Disease Relationships), supporting an overall neuroprotective role now widely accepted as a functional label for this Lipocalin. Both astrocytes and oligodendrocytes express and secrete ApoD in response to stress. The protein exerts an autocrine and paracrine neural tissue protection, which results in functional preservation of OS-challenged dopaminergic systems, of neurons affected by kainate excitotoxicity or suffering from Aβ-related degeneration. Astrocytes, although not an abundant source of ApoD in basal conditions, quickly respond to OS with a JNK-dependent expression of ApoD, which is secreted to the extracellular milieu as cargo on the surface of extracellular vesicles (Figure 6). The protein is internalized by glial and neuronal cells, improving their viability thanks to a control by ApoD of OS-dependent lipid peroxide accumulation. Moreover, a surge of ApoD in a stressed neural tissue behaves as an off-signal limiting the dimension and duration of gliosis and inflammation. The inflammatory response is linked to OS due to increased PLA2 expression and AA production, among other factors. Quenching of AA is proposed as part of this inflammation control by ApoD (see section Protein Physiology).

A long-lasting homeostasis maintenance role for ApoD has been also proposed in the process of physiological aging of the nervous system, where this protein has been shown as the most consistently overexpressed in primates and rodents. Also, life-expanding strategies in model organisms, like caloric restriction, promote ApoD expression not only in the nervous system but also in cardiac and skeletal muscle (Supplementary Table 11). The homeostatic role predicted by the expression pattern is supported by the phenotypes exhibited by aged ApoD-KO mice, which do not display altered lifespan but do present signs of early neurodegeneration at 3 months of age, with oxidative damage and proteostasis defects in cortex and hippocampus. These alterations underlie cognitive defects and a hyperkinetic phenotype evident in old (21 months) ApoD-KO mice.

The predominant expression of ApoD in myelinating cells under control conditions (oligodendrocytes in CNS and Schwann cells in PNS; see section Regulation of Expression) has prompted experimental studies, using cultured primary cells and ApoD-KO and hApoD-Tg mice, that clearly support the implication of ApoD in the myelination process during development, in the lifelong maintenance of the myelin sheath, and in the remyelination that occurs in response to environmental insults. These processes have been analyzed in the mouse brain corpus callosum as well as in the peripheral sciatic nerve. ApoD is required for a proper and timely response to a crush injury in PNS nerves, helping to recover locomotor function. ApoD promotes myelin clearance and regulates angiogenesis and macrophages recruitment to the wound site, processes that are essential for subsequent axonal regeneration and remyelination. ApoD contributes to optimize myelin clearance, carried out by transdifferentiated Schwann cells and infiltrating macrophages, through two complementary actions: control of lipid-mediated inflammatory signaling and optimization of the phagocytosis process itself. Data indicates that ApoD regulates and control the tissue levels of AA and lysophosphatidylcholine (both *in vitro*-demonstrated ApoD ligands, Table 2). They are needed for an adequate cytokine inflammatory response and recruitment of bone marrow-derived macrophages. Although macrophages do not express ApoD, the levels of this Lipocalin in the injured nerve environment influence their phagocytic activity, since myelin-associated ApoD is phagocytosed as well. Flow cytometry experiments with primary macrophages demonstrated that ApoD affects the initiation and efficacy of phagocytosis.

A dynamic spatiotemporal regulation of ApoD expression is apparent in myelinating cells, with a prominent increase at the height of postnatal myelination followed by continuous rise throughout life. The absence of ApoD results in a defective and irreversible compaction, mostly in the extracellular leaflet of both CNS and PNS myelin. This altered myelin structure results in a decreased conduction velocity, reported for the sciatic nerve, and compromises motor learning tasks. As downstream effects, both the mTORC1-dependent lipogenic switch and the ERK-mediated growth pathways are altered in the absence of ApoD. A lack of myelin compaction is due to inadequate removal of myelin glycocalyx, mostly affecting gangliosides GM1–2b, GD1b, and GT1b content and distribution. This role of ApoD on glycocalyx physiology was demonstrated to be linked to the adequate subcellular localization of lysosomal and plasma membrane sialidase (Neu1 and Neu3) and of the regulatory Fyn kinase. This mechanism requires preservation of lysosomal membrane integrity (see section Protein Physiology).

References contributing to this section are listed in **Reference Collection 39**, Supplementary Table 7.

### Protein Physiology

In this final section we aim at discussing the available knowledge, derived from state-of-the-art research critically assessed in this review, to give a plausible answer to the central question posed in the Introduction: In order to achieve its pleiotropic roles, does ApoD moonlight between different biochemical functions when expressed in different contexts, or instead ApoD displays a distinctive biochemical role that works on varied physiological situations?

The presence of ApoD in extracellular formats such as lipoprotein particles and exosomes evidences its ability to associate to higher-order lipid structures. According to our systematic search no unambiguous evidence exists for the secretion of protein monomers in native conditions. Non-denaturing electrophoresis analysis of CSF revealed only high-molecular weight ApoD oligomers, while in plasma it has been repeatedly identified in lipoprotein particles preparations. In the particular case of BCF, where an extremely high concentration of ApoD is produced, the protein assembles in tetramers through protein-protein interactions. Finally, ApoD secreted by cultured astrocytes is internalized by neurons only if the conditioned extracellular media has not been depleted of extracellular vesicles. A protein region encompassing the first three β-strands, highly conserved in chordates (Figure 2A), and several hydrophobic patches located at the protein pocket entrance (Figure 3E) are proposed to underlie ApoD self-association and ApoD-lipid structure interactions, respectively.

These results shed doubts on a view of ApoD widely cited (a functional tag in most databases for this Lipocalin) as a “lipid transporter,” a task that a secreted globular monomer could easily achieve.

Unquestionably though, it is the ability of ApoD to bind small hydrophobic ligands of varied shapes inside its β-barrel pocket. However, when free ligands (e.g., AA) have been mechanistically related to ApoD function, binding data are compatible with a buffering or quenching function, or a very local shuttling of the ligand at the most, rather than to a generalized long-range ligand transport between cells. A curious case is the expression of ApoD in feather follicles of pheasants, only in skin areas with specific plumage colors, suggestive of a pigment-retention function. Similarly, the ligand bound to ApoD in sweat from human axilla could be the source of slowly released volatile odor molecules. These ligand-retention functions compare well with that of crustacean ApoD homologs, also linked to their carapace coloration (Wade et al., 2009).

In a different context, ligand shuttling has been repeatedly proposed for ApoD in the cholesterol transfer to LCAT. However, ApoD has been demonstrated not to bind cholesterol, not to contribute to LCAT-cholesterol transfer and not to show a direct interaction with LCAT. On the contrary, experimental data suggests that ApoD exerts “stabilizing effects” on LCAT activity. After reviewing the relevant information on this issue, we propose a different view that can guide new testable hypotheses: ApoD binds lysophosphatidylcholine (LPC), a LCAT reaction product that exerts a negative feedback on LCAT activity. By quenching LPC, ApoD would maintain LCAT activity over a wide range of LPC product concentration. This specific LPC quenching function is compatible with the small amounts of ApoD recovered from HDLs, since only a transitory presence of ApoD might be needed when LCAT is adding cholesterol to the lipoparticle. As for the HDL-LDL interaction (see section Roles of ApoD in Metabolism Regulation), the putative consequences of ApoD presence in HDLs on cholesterol management in the organism would therefore be of an indirect nature, and could explain the lack of correlation between ApoD and cholesterol content in many physiological or pathological situations.

A fundamental advance in defining ApoD molecular function was its role in organismal protection against OS, achieved by a control of the magnitude of lipid peroxidation, measured at tissue or cellular levels. This role has received strong experimental support from *in vitro* biochemical assays, cellular experimental systems, and *in vivo* experiments with animal models where ApoD expression was manipulated. Moreover, further validation for this role comes from experimental approaches testing the expression of human ApoD in evolutionary distant organisms. Overexpression of human ApoD in Drosophila increases lifespan in both normal and pro-oxidative experimental conditions. Also, replacement in plants of the native chloroplast Lipocalin (LCNP) by human ApoD, targeted to thylakoids, rescues drought and OS sensitivity of the mutant. Lipid peroxidation control is evidenced in both reports as the mechanism mediating the organism response.

An ApoD antioxidant mechanism has been demonstrated using oxidized AA-derivatives in solution or auto-oxidized liposomes. ApoD is able to reduce free radical-generating lipid hydroperoxides to inert lipid hydroxides. In this reaction, the residue Met93 exposed on one of the surface hydrophobic patches of the protein (Figure 3C) is converted to Met93-sulfoxide. This residue is preserved in ApoD chordate orthologs (Figure 2A) and contributes to the functional differentiation of ApoD from its closest Lipocalin relative, RBP4, where that position is occupied by charged (Lys or Arg) residues (Diez-Hermano et al., 2021). To maintain ApoD antioxidant activity, the action of a methionine sulfoxide reductase (MRS) would be required. However, oxidized ApoD tends to self-associate. Interesting data from Alzheimer's disease brain samples reveal that hippocampal (but not cerebellar) MRS levels decrease with disease progression, while ApoD oligomerization increases. This suggests that the ApoD redox cycling might be blocked if Met93 does not return to its native form and the protein self-associates. This effect sets an upper limit to ApoD antioxidant activity, since it would result in the consumption of ApoD-Met93. Whether this depletion triggers a feedback regulatory loop promoting ApoD gene expression under OS situations in different physiological and pathological contexts would be an interesting aspect to explore. In this context, we must keep in mind that ApoD structure is stable under pro-oxidative situations, making it suitable for the biological contexts where ApoD function is beneficial (from neurodegenerative conditions to cancer). Also, ApoD ligand binding ability is preserved at low pH and its glycosylation prevents a rapid degradation, both good assets to perform its ligand binding and antioxidant functions inside the endolysosomal compartment.

The direct antioxidant activity of ApoD and its demonstrated stable location in the lysosomal compartment put forward a new view of ApoD protein physiology that holds high explanatory power in the understanding of a number of apparently varied ApoD roles.

ApoD control of redox state can be performed directly on both, cell membranes and lipoprotein particles. The unilamellar vesicles where ApoD-reducing activity has been demonstrated are a good experimental model for both types of lipid-based structures. Lipid peroxide products are mainly derived from cellular membranes, which are a major target for cell-generated ROS. The ability of ApoD to keep low levels of membrane-originated lipid peroxides, together with the positive correlation of ApoD content in HDLs with their antioxidant capacity, and the promotion of HDL-LDL interaction by exogenously-added native ApoD, support the protective action of ApoD in both types of lipidic structures (membranes and lipoparticles). Additional evidence comes from the existence of ApoD insect homologs stably anchored to cell membranes (Ganfornina et al., 1995; Ruiz García, 2013), which suggests that membrane interaction is part of an ancestral ApoD property.

In addition to the immediate effects on the redox state of membranes and other lipid structures, ApoD can give rise to indirect effects when performing its antioxidant function in the lysosome. The lysosome is considered a “lipid-controlling” cellular hub. ApoD maintenance of lysosomal membrane redox balance and integrity results in the control of plasma membrane composition. This is for example the case for plasma membrane glycolipids, with important consequences for membrane-membrane interactions like those required in the process of myelin compaction. Lysosomal membrane stability can, by extension, influence the lipid export/import balance in cells, another way of ApoD indirectly conditioning the organism lipid metabolism. Altered ApoD expression in response to mutations of the lysosomal cholesterol transporters (as in Niemann-Pick type C disease) supports this notion. Plasma membrane modulation is also coherent with the observed correlation of ApoD content in HDLs and their ABCA1-dependent cholesterol efflux capability in macrophages, or the subtle changes in lipid content in lipoprotein particles of subjects with ApoD polymorphisms. Through its influence on membranes and lipoparticle dynamics, without a need of binding cholesterol, ApoD can modulate its flux within and between cells.

Additionally, the lysosome is a “cell death/survival controller” by its fundamental recycling, detoxifying and proteostatic functions. Lysosomal ApoD would condition whether a failure in the lysosomal compartment takes place upon a wide array of disease/injury situations, thus contributing to the final cell fate. This ApoD-dependent cell fate decision can be extended to developmental processes as well.

Finally, a role of ApoD in innate immunity has been frequently reported, while no mechanistic link to the protein physiology was proposed. We suggest that ApoD, with its lysosomal optimization mechanism, can modulate the efficiency of phagocytic cells, like it has been demonstrated in injury-recruited macrophages, therefore influencing many of the maintenance and immune responses of the organism.

This view makes us to propose that ApoD lipid-binding properties are more related to management of lipid-based structures composition (membranes or lipoparticles) and a control of their redox state, than to lipid transport. Whether similar membrane-stabilizing properties endow ApoD-positive exosomes with resistance properties to be efficient cargo transporters in disease or tissue damage situations, would be worth studying.

A different aspect of ApoD physiology scarcely studied is the role of its demonstrated N-linked glycosylation, which has been proven to be tissue and species specific, and to be essential for both, ApoD interaction with lipoparticles and for its cellular localization in the endolysosomal compartment. This is particularly important because of the association between redox signaling and glycan profiles, which in turn could affect several signaling pathways (Khoder-Agha and Kietzmann, 2021). In relation to this, modulation of signaling pathways by ApoD has been confirmed in endothelial cells and osteoblasts (PI3K-Akt pathway) and nervous tissue (pERK). How ApoD controls signaling cascades is open to discussion. Although several protein candidates have been proposed as ApoD membrane receptor, no clear demonstration is available for a receptor-mediated signaling transduction. Alternatively, ApoD might not require a protein receptor and trigger a unique signaling cascade. Instead, it could be working as a quencher of lipid modulators (e.g., AA), or conditioning the membrane partitioning of signaling complexes that are known to be dependent on membrane lipids distribution.

In summary, the available information supports a parsimonious hypothesis for the biological function of ApoD, with a unique biochemical role related to the management and redox state of lipid cellular and extracellular structures. This proposition is compatible with the wealth of experimental results showing that multiple stimuli in varied cellular contexts trigger ApoD expression with a tight spatiotemporal regulatory control. The protein can then become associated with the challenged membranes or being exported to the extracellular milieu to act in a paracrine fashion. Both direct and indirect downstream effects, depending on the cell type affected, would explain pleiotropy at the organismal level with a single biochemical function.

**Figure 7:**
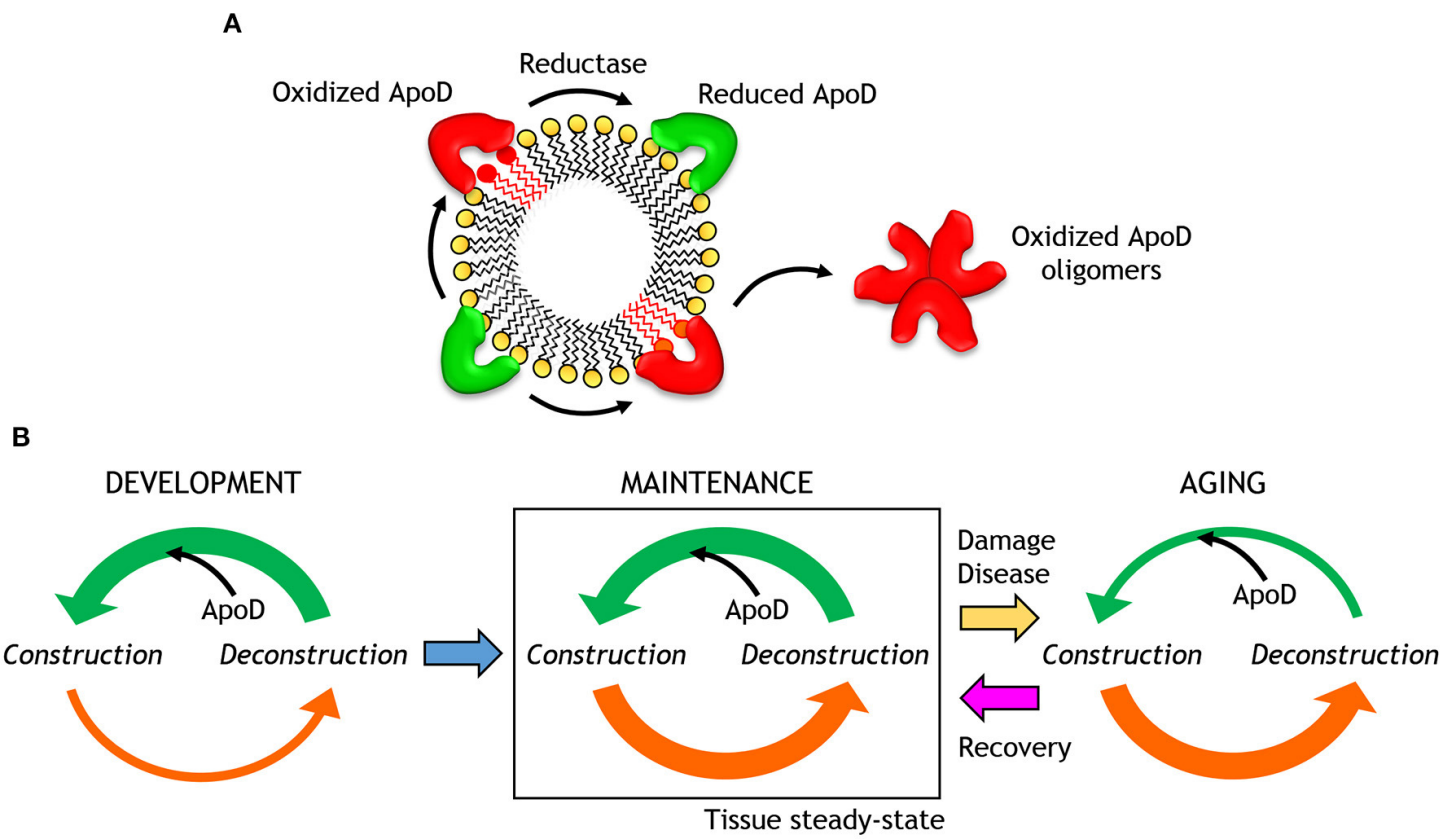
ApoD physiology summary. **(A)** Schematic view of the lipid-managing biochemical function of ApoD. The lipid structure depicted can equally represent the surface of a lipoprotein particle, extracellular vesicle or cellular membrane. ApoD antioxidant activity can be maintained by redox cycling, requiring a reductase activity, or the cycle can terminate by oligomerization of oxidized ApoD. **(B)** Summary of global tissue function of ApoD, where it contributes to the turnover and maintenance of tissues and organs. This equilibrium is reached after developmental processes in which ApoD is also involved, and switches to a different state upon disease, injury or physiological aging.

The proposed unique molecular mechanism also explains ApoD biological role in response to tissue/organ damage and disease, where homeostatic maintenance is disturbed and ApoD will contribute to restore the equilibrium through tissue repair/reconstruction. Under this paradigm, we can also explain ApoD roles in organismal developmental processes implying building-deconstruction cycles. Figure 7 summarizes the new view on ApoD physiology. References contributing to this section are listed in **Reference collection 40**, Supplementary Table 8.

### Future Goals for ApoD Biology

In spite of the explanatory power of our proposed biological role for ApoD, many questions keep been unresolved and many others are likely to arise, which can spur and guide new research programs. A few of them follow:

1. To explore the functional relationship between the protein antioxidant capacity and the pocket ligand binding. In this respect, the hypothesis of ApoD working on oxidized lipid “whiskers” (Greenberg et al., 2008; Del Caño-Espinel, 2014) on cell membrane bilayers or lipoparticles is appealing and worth contrasting.
2. To test whether ApoD downstream effects on signaling pathways rely on a canonical receptor-mediated transduction, or alternatively they depend on the modulation of the lipid context of signaling elements (e.g., PI3K). Findings in Drosophila reveal that loss of an ApoD homolog alters PI3K association to the plasma membrane (Hull-Thompson et al., 2009).
3. To test whether oligomeric *vs*. monomeric forms of ApoD underlie its managing function on cell membranes or lipoparticles.
4. To characterize the extent of ApoD redox cycle, maintaining antioxidant ApoD activity thanks to the intervention of reductases, and the implications of a potential upper limit to this mechanism due to ApoD oligomerization. This aspect can be key to fully understand ApoD function in aging and disease.
5. To analyze the effects of differential glycosylation on ApoD interactions and functions.
6. Recent studies on a Drosophila homolog (Yin et al., 2021) point to lipid droplets as another higher-order lipid structure susceptible to be modulated by ApoD. Searching for lipid droplet-managing functions of vertebrate ApoD is therefore pertinent.
7. At a more general tissue/organ level, several functions are relevant to be studied in more depth, such as the ApoD role on feather and skin physiology, neuronal synaptic function, and metabolism.
8. Finally, it is worth to analyze the potential exchange between the nervous system and systemic pools of extracellular ApoD in its different formats, not only to fully understand its roles in the organism, but also for a potential therapeutic use of ApoD in nervous system diseases.

## ApoD Systematic Review. Reference Collections

**Reference Collection 1** (McConathy and Alaupovic, 1973, 1976, 1986; Camato et al., 1989; Kamboh et al., 1989; Holmquist, 1990; Weinberg, 1994; Yang et al., 1994; Holzfeind et al., 1995; Terrisse et al., 2001; Salvatore et al., 2007).

**Reference Collection 2** (Eichinger et al., 2007; Oakley et al., 2012; Kielkopf et al., 2018, 2019, 2021).

**Reference Collection 3** (McConathy and Alaupovic, 1976, 1986; Bojanovski et al., 1980; Yang et al., 1994; Schindler et al., 1995; Zeng et al., 1996; Sun et al., 1998; Perdomo and Henry Dong, 2009; Li et al., 2016; Qin et al., 2017).

**Reference Collection 4** (Blanco-Vaca and Pownall, 1993; Holzfeind et al., 1995; Patel et al., 1997; Böttcher et al., 2000; Nasreen et al., 2006; Bhatia et al., 2012, 2013; Kielkopf et al., 2018, 2019, 2021).

**Reference Collection 5** (Haagensen et al., 1979; Lea, 1988; Dilley et al., 1990; Peitsch and Boguski, 1990; Morais Cabral et al., 1995; Zeng et al., 1996; Goessling and Zucker, 2000; Vogt and Skerra, 2001; Breustedt et al., 2006; Akiba et al., 2011; Oakley et al., 2012; Ruiz et al., 2013; García-Mateo et al., 2014; Kielkopf et al., 2019, 2021).

**Reference Collection 6** (Fielding and Fielding, 1980; Morton and Zilversmit, 1981; Mahadevan and Soloff, 1983; Steyrer and Kostner, 1988; Camato et al., 1989; Francone et al., 1989; Holmquist, 1989; Sánchez et al., 1992a).

**Reference Collection 7** (Utermann et al., 1980; Camato et al., 1989; Yang et al., 1990, 1994; Blanco-Vaca et al., 1992; Liu et al., 2001; Jin et al., 2006; Najyb et al., 2015; Lai et al., 2017).

**Reference Collection 8** (McConathy and Alaupovic, 1973, 1976, 1986; Bojanovski et al., 1980; Borghini et al., 1995; Böttcher et al., 2000; Goessling and Zucker, 2000; Perdomo and Henry Dong, 2009; Bhatia et al., 2012; Sreckovic et al., 2013; Braesch-Andersen et al., 2014; Singh et al., 2016; Pascua-Maestro et al., 2018).

**Reference Collection 9** (Drayna et al., 1986, 1987; Warden et al., 1992; Provost et al., 1995; Séguin et al., 1995; Cofer and Ross, 1996; Yoshida et al., 1996; Diez-Hermano et al., 2020; Sałkowska et al., 2020).

**Reference Collection 10** (Lambert et al., 1993; Yoshida et al., 1996; Do Carmo et al., 2002, 2007; Yamashita et al., 2002; van den Boom et al., 2006; Sasaki et al., 2009; Levros et al., 2010, 2013; Tapia et al., 2011; Ammerpohl et al., 2013; Bajo-Grañeras et al., 2013; Namdar-Aligoodarzi et al., 2015; Diez-Hermano et al., 2020; Sałkowska et al., 2020; Wu et al., 2021).

**Reference Collection 11** (Provost et al., 1995; Séguin et al., 1995; Namdar-Aligoodarzi et al., 2015; Lim et al., 2016; Diez-Hermano et al., 2020).

**Reference Collection 12** (Drayna et al., 1987; Eichner et al., 1989; Kamboh et al., 1989; Baker et al., 1994; Vijayaraghavan et al., 1994; Desai et al., 2002, 2003; Helisalmi et al., 2004; Hansen et al., 2006; Zhang et al., 2006; Chen et al., 2008; Shibata et al., 2013; Lövkvist et al., 2014; Guven et al., 2019).

**Reference Collection 13** (Holmquist, 1990; Holmquist and Vesterberg, 1991; Vizoso et al., 1992, 1994; Blanco-Vaca et al., 1994; Thalmann et al., 1994; Holzfeind et al., 1995; Vieira et al., 1995; Holmquist et al., 1996; Sun et al., 1998; Suresh et al., 1998; Harding et al., 2000; Koch et al., 2001; Yao and Vieira, 2002; Baechle et al., 2006; Do Carmo et al., 2009a; Perdomo and Henry Dong, 2009; Perdomo et al., 2010; Liu et al., 2013; Sreckovic et al., 2013; Ali et al., 2014; Mattsson et al., 2014; Csosz et al., 2015; Waldner et al., 2018; Xu et al., 2018;Kopylov et al., 2020).

**Reference Collection 14** (Drayna et al., 1986; Provost et al., 1990, 1991b, 1995; Smith et al., 1990; Spreyer et al., 1990; Escribano et al., 1995; Gillen et al., 1995; Holzfeind et al., 1995; Schaeren-Wiemers et al., 1995; Séguin et al., 1995; Cofer and Ross, 1996; Yoshida et al., 1996; Zeng et al., 1996; Hardardóttir et al., 1997; Suresh et al., 1998; Franz et al., 1999; Montpied et al., 1999; Ong et al., 1999; Terrisse et al., 1999; Zhou et al., 2000; Belloir et al., 2001; Liu et al., 2001; Thomas et al., 2001a,c; Kao et al., 2002; Sánchez et al., 2002; del Valle et al., 2003; Tomarev et al., 2003; Ganfornina et al., 2005, 2008; Hildebrand et al., 2005; Yang et al., 2005; Del Signore et al., 2006; Chen et al., 2007, 2011; Molnár et al., 2007; Zhang et al., 2007, 2014; Befort et al., 2008; Do Carmo et al., 2008, 2009a; Loerch et al., 2008; Rickhag et al., 2008; Collins-Racie et al., 2009; Perdomo and Henry Dong, 2009; Schäfer et al., 2009; Bianchi-Frias et al., 2010; Navarro et al., 2010b; Qin et al., 2010; Bajo-Grañeras et al., 2011a, 2013; Walsh et al., 2012; Germeyer et al., 2013; Manousaki et al., 2013; Edlow et al., 2014; García-Mateo et al., 2014, 2018; Hatzirodos et al., 2015; Jeong et al., 2015; Sanchez et al., 2015; Zheng et al., 2015; Zhu et al., 2015; Gao et al., 2016; Lim et al., 2016; Tao et al., 2017; Carnes et al., 2018; Desmarais et al., 2018; Piórkowska et al., 2018; Flores et al., 2019; Jensen et al., 2019; Diez-Hermano et al., 2020).

**Reference Collection 15** (Bouma et al., 1988; Boyles et al., 1990a,b; Escribano et al., 1995; Navarro et al., 1998, 2004, 2008, 2010a,b; Suresh et al., 1998; Franz et al., 1999; Ong et al., 1999, 2002; Kalman et al., 2000; Belloir et al., 2001; Hu et al., 2001; Thomas et al., 2001b; del Valle et al., 2003; Khan et al., 2003; Desai et al., 2005; Hildebrand et al., 2005; Del Signore et al., 2006; Ordoñez et al., 2006; Chen et al., 2007, 2011, 2013; Schröder et al., 2007; Ganfornina et al., 2008; Rickhag et al., 2008; Do Carmo et al., 2009a; Jansen et al., 2009; Qin et al., 2010; Bajo-Grañeras et al., 2011a, 2013; Delattre et al., 2012; Ordóñez et al., 2012; Germeyer et al., 2013; García-Mateo et al., 2014, 2018; Labrie et al., 2015; Martínez-Pinilla et al., 2015; Sanchez et al., 2015; Zheng et al., 2015; Li et al., 2016; Lim et al., 2016; Soria et al., 2017; Desmarais et al., 2018; Flores et al., 2019; Jensen et al., 2019; El-Darzi et al., 2020; Pascua-Maestro et al., 2020).

**Reference Collection 16** (Bouma et al., 1988; Boyles et al., 1990a; Schaeren-Wiemers et al., 1995; Vieira et al., 1995; Zeng et al., 1996; Ong et al., 1997; Franz et al., 1999; Kalman et al., 2000; Belloir et al., 2001; Hu et al., 2001; Navarro et al., 2004; Desai et al., 2005; Ganfornina et al., 2005; Hildebrand et al., 2005; Ishii et al., 2005; Loerch et al., 2008; Rickhag et al., 2008; Song et al., 2009; Bajo-Grañeras et al., 2013; Germeyer et al., 2013; García-Mateo et al., 2014; Martínez-Pinilla et al., 2015; Zheng et al., 2015; Li et al., 2016; Flores et al., 2019; Kendal et al., 2020; Pascua-Maestro et al., 2020; Sałkowska et al., 2020;Schlotter et al., 2020).

**Reference Collection 17** (Simard et al., 1990, 1991, 1992; Sugimoto et al., 1994; Patel et al., 1995; Harding et al., 2000; Zhou et al., 2000; Yao and Vieira, 2002; Appari et al., 2009; Do Carmo et al., 2009a; Wang et al., 2010; Chen et al., 2011; Tapia et al., 2011; Delattre et al., 2012; Eigeliene et al., 2012; Pérez et al., 2012; Germeyer et al., 2013; Jeong et al., 2015; Tao et al., 2017; Kfir et al., 2018; Hornig et al., 2019; Igarashi et al., 2020).

**Reference Collection 18** (Loerch et al., 2008; de Magalhães et al., 2009; Kim et al., 2009; Bianchi-Frias et al., 2010; Navarro et al., 2010b, 2013; Martínez et al., 2012, 2013; Ordóñez et al., 2012; Yan et al., 2012; Sanchez et al., 2015; Martineau et al., 2016; Kosarussavadi et al., 2017; García-Mateo et al., 2018; Waldner et al., 2018; Yu et al., 2020).

**Reference Collection 19** (Do Carmo et al., 2007; Ganfornina et al., 2008; Nowicki et al., 2009; Sasaki et al., 2009; Bajo-Grañeras et al., 2011a,b, 2013; Martínez et al., 2013; Dassati et al., 2020; Diez-Hermano et al., 2020; Martínez-Pinilla et al., 2021).

**Reference Collection 20** (Blais et al., 1994, 1995; Gillen et al., 1995; Hardardóttir et al., 1997; Ong et al., 1997; Montpied et al., 1999; Trieu and Uckun, 2000; Kim et al., 2001; Leung et al., 2004; Del Signore et al., 2006; Do Carmo et al., 2007, 2008; Rickhag et al., 2008; Qin et al., 2010; Chia et al., 2011; Puntambekar et al., 2011; García-Mateo et al., 2014; Najyb et al., 2017; Scalf et al., 2019; Schlotter et al., 2020).

**Reference Collection 21** (Suresh et al., 1998; Hargreaves et al., 1999; Hummasti et al., 2004; Bujalska et al., 2006; Cha et al., 2006; Zhang et al., 2007; Perdomo et al., 2010; Kosacka et al., 2011; Ali et al., 2014; Edlow et al., 2014; Zheng et al., 2015; Lim et al., 2016; Lai et al., 2017; Lambert et al., 2017; Desmarais et al., 2018; Piórkowska et al., 2018; Xu et al., 2018;Saadane et al., 2019).

**Reference Collection 22** (Provost et al., 1991b; López-Boado et al., 1994, 1996; Do Carmo et al., 2002, 2007; Kang et al., 2003a; Sarjeant et al., 2003; Bianchi-Frias et al., 2010; Levros et al., 2010; Bajo-Grañeras et al., 2011a; Braesch-Andersen et al., 2014; Martineau et al., 2016).

**Reference Collection 23** (Tew et al., 2007; Kosacka et al., 2009, 2011; Pajaniappan et al., 2011; Jensen et al., 2019).

**Reference Collection 24** (López-Boado et al., 1997; Thomas et al., 2001a, 2003b; O'Donnell et al., 2003; Befort et al., 2008; Dassati et al., 2020).

**Reference Collection 25** (Provost et al., 1991a; Patel et al., 1995; Suresh et al., 1998; Do Carmo et al., 2002; Kao et al., 2002; Kang et al., 2003a; Sarjeant et al., 2003; Leung et al., 2004; Ishii et al., 2005; Bujalska et al., 2006; Fujita et al., 2007; Tew et al., 2007; Kosacka et al., 2009; Pajaniappan et al., 2011; Germeyer et al., 2013; García-Mateo et al., 2014; Martineau et al., 2016; Flores et al., 2019; Saadane et al., 2019; Igarashi et al., 2020; Pascua-Maestro et al., 2020).

**Reference Collection 26** (Simard et al., 1990, 1991, 1992; Blais et al., 1994, 1995; López-Boado et al., 1994, 1996, 1997; Sugimoto et al., 1994; Do Carmo et al., 2002, 2007; Sarjeant et al., 2003; Hummasti et al., 2004; Leung et al., 2004; Cha et al., 2006; Tew et al., 2007; Kosacka et al., 2009, 2011; Sasaki et al., 2009; Bianchi-Frias et al., 2010; Levros et al., 2010; Martínez et al., 2012, 2013; Bajo-Grañeras et al., 2013; Braesch-Andersen et al., 2014; Martínez-Pinilla et al., 2015, 2021; Martineau et al., 2016; Lai et al., 2017; Pascua-Maestro et al., 2018; Flores et al., 2019; Dassati et al., 2020).

**Reference Collection 27** (Boyles et al., 1990a; Spreyer et al., 1990; Gillen et al., 1995; Yoshida et al., 1996; Dandoy-Dron et al., 1998; Suresh et al., 1998; Terrisse et al., 1998, 1999; Franz et al., 1999; Kalman et al., 2000; Trieu and Uckun, 2000; Belloir et al., 2001; Kim et al., 2001; Lieuallen et al., 2001; Reindl et al., 2001; Thomas et al., 2001b,c, 2003a,d; Mahadik et al., 2002; Ong et al., 2002; Desai et al., 2003, 2005; Glöckner and Ohm, 2003; Kang et al., 2003b; Navarro et al., 2003, 2018; Saha and Rangarajan, 2003; Helisalmi et al., 2004; Li et al., 2005, 2006, 2015; Yao et al., 2005; Hansen et al., 2006; Ordoñez et al., 2006; Rickhag et al., 2006, 2008; Zhang et al., 2006; Almgren et al., 2008; Chen et al., 2008; Dean et al., 2008; Do Carmo et al., 2008; Song et al., 2009; Ruscher et al., 2010; Kroksveen et al., 2012, 2013; Ordóñez et al., 2012; Martínez et al., 2013; Shibata et al., 2013; García-Mateo et al., 2014; Lövkvist et al., 2014; Mattsson et al., 2014; Martínez-Pinilla et al., 2015; Oláh et al., 2015; Del Valle et al., 2016; Lee et al., 2016; Przybycien-Szymanska et al., 2016; Qin et al., 2017; Waldner et al., 2018; Bhatia et al., 2019; Guven et al., 2019; Jiang et al., 2019; Khoonsari et al., 2019; Kuiperij et al., 2019; Perrotte et al., 2019; Zou et al., 2019; Pascua-Maestro et al., 2020; Watanabe et al., 2020; Zhou et al., 2020; Tristán-Noguero et al., 2021; Vardi et al., 2021).

**Reference Collection 28** (Balbín et al., 1990; Mazoujian and Haagensen, 1990; Simard et al., 1990, 1991, 1992; Søreide et al., 1991a,b, 1994; Sánchez et al., 1992a,b; Díez-Itza et al., 1994; Aspinall et al., 1995; Chen et al., 1995; Lane et al., 1995; Hall et al., 1996, 2004; López-Boado et al., 1996, 1997; Osundeko et al., 1997; Zhang et al., 1998; Clements et al., 1999; Serra Díaz et al., 1999; Lamelas et al., 2000; Rodríguez et al., 2000; Satoh et al., 2000; Serra et al., 2000; Vázquez et al., 2000; Rojo et al., 2001; Selim et al., 2001; Gutmann et al., 2002; Hunter et al., 2002, 2005a,b; Yamashita et al., 2002; Alvarez et al., 2003, 2004; Linn et al., 2003; Miranda et al., 2003; Alexander et al., 2004; Ashida et al., 2004; Baris et al., 2004; West et al., 2004; Kristensen et al., 2005; Lynch et al., 2005; Ogawa et al., 2005; Ricci et al., 2005; Utsunomiya et al., 2005; Colin et al., 2006; Doane et al., 2006; Jin et al., 2006; van den Boom et al., 2006; Bianchi-Frias et al., 2007, 2010; Carreño et al., 2007; Gonzalez et al., 2007; Vizoso et al., 2007; Yokoo et al., 2007; Galamb et al., 2008; Lisovsky et al., 2008; Quaresima et al., 2008; Song et al., 2008; Fontaine et al., 2009; Søiland et al., 2009a,b; Jinawath et al., 2010; Bidinotto et al., 2012; Palmerini et al., 2012; Bajo-Grañeras et al., 2013; Wang et al., 2013; Jeong et al., 2015; Allina et al., 2016; Sandim et al., 2016; Klebaner et al., 2017; Hu et al., 2018; Cury et al., 2019; Li et al., 2019; Mohammed et al., 2019; Jankovic-Karasoulos et al., 2020; Jiang et al., 2020; Mirza and Abdel-Dayem, 2020; Lopez-Nunez et al., 2021; Wu et al., 2021).

**Reference Collection 29** (Wiklund et al., 1980; James et al., 1986; Lin et al., 2001, 2019; Sarjeant et al., 2003; Wei et al., 2008; Perdomo and Henry Dong, 2009; Tsukamoto et al., 2013; Pleva et al., 2015; Cheow et al., 2016; Pamir et al., 2016; Sulkava et al., 2017; Contreras-Duarte et al., 2018; Nazarenko et al., 2018; Caparosa et al., 2019; Ng et al., 2019; Sun et al., 2019; Schlotter et al., 2020).

**Reference Collection 30** (Curry et al., 1977; Albers et al., 1981; Cheung et al., 1993; Baker et al., 1994; Vijayaraghavan et al., 1994; Hansen et al., 2004; Iqbal et al., 2008; Navarro et al., 2010a; Edlow et al., 2014; Manjunatha et al., 2016; Ravnsborg et al., 2016; Brahimaj et al., 2017; Herzig et al., 2017; Desmarais et al., 2018; Saadane et al., 2019; Kopylov et al., 2020; Santana et al., 2020).

**Reference Collection 31** (Bujalska et al., 2006; Appari et al., 2009; Chen et al., 2011; Allegra et al., 2012; Ammerpohl et al., 2013; Zhu et al., 2015; Salami et al., 2019; Tanase-Nakao et al., 2019; Geng et al., 2020).

**Reference Collection 32** (Albers et al., 1984; Haffner et al., 1985; Holmquist et al., 1993; Gottsch et al., 2003; Zenkel et al., 2005; Flatscher-Bader and Wilce, 2006; Tew et al., 2007; Collins-Racie et al., 2009; Schäfer et al., 2009; Sun et al., 2013; Aregger et al., 2014; Hueging et al., 2015; Kliuchnikova et al., 2016; Starodubtseva et al., 2016; Soria et al., 2017; Deng et al., 2018; Forster et al., 2019; Ponnikorn et al., 2019;Liu et al., 2020).

**Reference Collection 33** (Bouma et al., 1988; Patel et al., 1995; Navarro et al., 1998, 2004; Suresh et al., 1998; Ong et al., 1999; Hu et al., 2001; Leung et al., 2004; Schröder et al., 2007; Søiland et al., 2009a,b; Ordóñez et al., 2012; Martínez-Pinilla et al., 2015; Klebaner et al., 2017; Pascua-Maestro et al., 2017, 2020; Schlotter et al., 2020).

**Reference Collection 34** (Simard et al., 1990, 1991, 1992; Blais et al., 1994; Patel et al., 1995; Suresh et al., 1998; Do Carmo et al., 2007; Sreckovic et al., 2013; Cheow et al., 2016; Pamir et al., 2016; Przybycien-Szymanska et al., 2016; Pascua-Maestro et al., 2017, 2018).

**Reference Collection 35** (Vieira et al., 1995; Sarjeant et al., 2003; Thomas et al., 2003c; Hildebrand et al., 2005; Najyb et al., 2015, 2017; Pascua-Maestro et al., 2017, 2018, 2020; Martínez-Pinilla et al., 2021).

**Reference Collection 36** (Sarjeant et al., 2003; Pajaniappan et al., 2011; Tsukamoto et al., 2013; Lai et al., 2017).

**Reference Collection 37** (Desai et al., 2002; Do Carmo et al., 2009b; Perdomo et al., 2010; Jiménez-Palomares et al., 2011; Ali et al., 2014; Braesch-Andersen et al., 2014; Labrie et al., 2015; Desmarais et al., 2019; El-Darzi et al., 2020).

**Reference Collection 38** (Martineau et al., 2016; Yu et al., 2020).

**Reference Collection 39** (Hildebrand et al., 2005; Thomas and Yao, 2007; Do Carmo et al., 2008; Ganfornina et al., 2008, 2010; He et al., 2009; Kosacka et al., 2009; Rajput et al., 2009; Boer et al., 2010; Bajo-Grañeras et al., 2011a,b; Kumar, 2012; Martínez et al., 2012; Ruiz et al., 2013; García-Mateo et al., 2014, 2018; Li et al., 2015; Sanchez et al., 2015; Najyb et al., 2017; Bhatia et al., 2019; El-Darzi et al., 2020; Pascua-Maestro et al., 2020).

**Reference Collection 40** (Steyrer and Kostner, 1988; Yamashita et al., 2002; Sarjeant et al., 2003; Thomas et al., 2003c; Jin et al., 2006; Eichinger et al., 2007; Thomas and Yao, 2007; Muffat et al., 2008; He et al., 2009; Kosacka et al., 2009; Sasaki et al., 2009; Ganfornina et al., 2010; Bajo-Grañeras et al., 2011a; Pajaniappan et al., 2011; Bhatia et al., 2012, 2013; Martínez et al., 2012; Oakley et al., 2012; Sreckovic et al., 2013; Braesch-Andersen et al., 2014; García-Mateo et al., 2014, 2018; Najyb et al., 2015; Gao et al., 2016; Martineau et al., 2016; Pamir et al., 2016; Lai et al., 2017; Pascua-Maestro et al., 2017, 2018, 2020; Kielkopf et al., 2018, 2019, 2021; El-Darzi et al., 2020;Henri and Rumeau, 2021).

## Data Availability Statement

The original contributions presented in the study are included in the article/Supplementary Materials, further inquiries can be directed to the corresponding author.

## Author Contributions

MG and DS: conceptualization, writing, review, and editing. Both authors contributed to the article and approved the submitted version.

## Funding

This work was supported by a Ministerio de Ciencia e Innovacion grant PID2019-110911RB-I00 to MG and DS.

## Conflict of Interest

The authors declare that the research was conducted in the absence of any commercial or financial relationships that could be construed as a potential conflict of interest.

## Publisher's Note

All claims expressed in this article are solely those of the authors and do not necessarily represent those of their affiliated organizations, or those of the publisher, the editors and the reviewers. Any product that may be evaluated in this article, or claim that may be made by its manufacturer, is not guaranteed or endorsed by the publisher.

## Supplementary Material

Supplementary Figure 1 ApoD mRNA and protein expression summary of the Human Protein Atlas.

Supplementary Figure 2 ApoD mRNA and protein expression summary of the Mouse Gene Expression Database.

Supplementary Table 1 Complete ApoD Reference Library.

Supplementary Tables 2–8 References organized by subheadings within each tag (MP, GD, RE, DR, CT, TOF, and PP).

Supplementary Tables 9–15 Analysis of primary papers describing ApoD mRNA expression or protein detection *in vivo* in different physiological systems: body fluids, reproductive system, nervous system, skin and glands, musculoskeletal system, cardiovascular and immune systems, and other functional systems (digestive system, liver, pancreas, adipose tissue, kidney).

Supplementary Tables 16, 17 Analysis of primary papers describing ApoD mRNA expression or protein detection in primary cultures and cell lines.

Supplementary Table 18 Analysis of primary papers describing ApoD relationships to disease (expression regulation by disease or treatment, genetic variations associated to disease).

## Abbreviations

AA: arachidonic acid
BCF: breast cyst fluid
CSF: cerebrospinal fluid
E-3M2H: E-3-methyl-2-hexenoic acid
ECs: endothelial cells
EM: electron microscopy
EVs: extracellular vesicles
GuHCl: guanidine hydrochloride
HDX-MS: amide hydrogen-deuterium exchange mass spectrometry
LPC: lysophosphatidylcholine
LPS: bacterial lipopolysaccharide
MCs: blood vessel mural cells
MSCs: bone marrow stem cells
OS: oxidative stress
RER: rough endoplasmic reticulum
ROS: reactive oxygen species
SAXS: small-angle X-ray scattering
STR: short-tandem repeats
TG: triglycerides
UTRs: gene untranslated regions.

## References

[B1] Akiba S. Arai N. Kusuoku H. Takagi Y. Hagura T. Takeuchi K. . (2011). The N-terminal amino acid of apolipoprotein D is putatively covalently bound to 3-hydroxy-3-methyl hexanoic acid, a key odour compound in axillary sweat. Int. J. Cosmet. Sci. 33, 283–286. 10.1111/j.1468-2494.2010.00636.x 21303379

[B2] Albers J. J. Cheung M. C. Ewens S. L. Tollefson J. H. (1981). Characterization and immunoassay of apolipoprotein D. Atherosclerosis 39, 395–409. 10.1016/0021-9150(81)90025-3 6789837

[B3] Albers J. J. Taggart H. M. Applebaum-Bowden D. Haffner S. Chesnut C. H. Hazzard W. R. (1984). Reduction of lecithin-cholesterol acyltransferase, apolipoprotein D and the Lp(a) lipoprotein with the anabolic steroid stanozolol. Biochim. Biophys. Acta 795, 293–296. 10.1016/0005-2760(84)90078-X 6236850

[B4] Alexander H. Stegner A. L. Wagner-Mann C. Du Bois G. C. Alexander S. Sauter E. R. (2004). Proteomic analysis to identify breast cancer biomarkers in nipple aspirate fluid. Clin. Cancer Res. 10, 7500–7510. 10.1158/1078-0432.CCR-04-1002 15569980

[B5] Ali K. Abo-Ali E. M. Kabir M. D. Riggins B. Nguy S. Li L. . (2014). A Western-fed diet increases plasma HDL and LDL-cholesterol levels in apoD-/- mice. PLoS ONE 9:e115744. 10.1371/journal.pone.0115744 25548917 PMC4280175

[B6] Allegra A. Marino A. Peregrin P. C. Lama A. García-Segovia A. Forte G. I. . (2012). Endometrial expression of selected genes in patients achieving pregnancy spontaneously or after ICSI and patients failing at least two ICSI cycles. Reprod. Biomed. Online 25, 481–491. 10.1016/j.rbmo.2012.07.019 22999554

[B7] Allina D. O. Andreeva Y. Y. Zavalishina L. E. Moskvina L. V. Frank G. A. (2016). Estimation of the diagnostic potential of APOD, PTOV1, and EPHA4 for prostatic neoplasms. Arkh. Patol. 78, 9–14. 10.17116/patol20167859-14 27804940

[B8] Almgren M. Nyengaard J. R. Persson B. Lavebratt C. (2008). Carbamazepine protects against neuronal hyperplasia and abnormal gene expression in the megencephaly mouse. Neurobiol. Dis. 32, 364–376. 10.1016/j.nbd.2008.07.025 18773962

[B9] Alvarez M. L. Barbón J. J. González L. O. Abelairas J. Boto A. Vizoso F. J. (2003). Apolipoprotein D expression in retinoblastoma. Ophthalmic Res. 35, 111–116. 10.1159/000069130 12646752

[B10] Alvarez M. L. Barbón J. J. González L. O. Lamelas M. L. Vázquez J. Vizoso F. J. (2004). Expression of two androgen-induced proteins (pepsinogen C and apolipoprotein d) in epithelial skin cancers of the eyelids. Ophthalmologica 218, 115–119. 10.1159/000076147 15004501

[B11] Ammerpohl O. Bens S. Appari M. Werner R. Korn B. Drop S. L. S. . (2013). Androgen receptor function links human sexual dimorphism to DNA methylation. PLoS ONE 8:e73288. 10.1371/journal.pone.0073288 24023855 PMC3762730

[B12] Appari M. Werner R. Wünsch L. Cario G. Demeter J. Hiort O. . (2009). Apolipoprotein D (APOD) is a putative biomarker of androgen receptor function in androgen insensitivity syndrome. J. Mol. Med. 87, 623–632. 10.1007/s00109-009-0462-3 19330472 PMC5518750

[B13] Aregger F. Uehlinger D. E. Witowski J. Brunisholz R. A. Hunziker P. Frey F. J. . (2014). Identification of IGFBP-7 by urinary proteomics as a novel prognostic marker in early acute kidney injury. Kidney Int. 85, 909–919. 10.1038/ki.2013.363 24067438

[B14] Ashida S. Nakagawa H. Katagiri T. Furihata M. Iiizumi M. Anazawa Y. . (2004). Molecular features of the transition from prostatic intraepithelial neoplasia (PIN) to prostate cancer: genome-wide gene-expression profiles of prostate cancers and PINs. Cancer Res. 64, 5963–5972. 10.1158/0008-5472.CAN-04-0020 15342375

[B15] Aspinall J. O. Bentel J. M. Horsfall D. J. Haagensen D. E. Marshall V. R. Tilley W. D. (1995). Differential expression of apolipoprotein-D and prostate specific antigen in benign and malignant prostate tissues. J. Urol. 154, 622–628. 10.1016/S0022-5347(01)67123-4 7541868

[B16] Baechle D. Flad T. Cansier A. Steffen H. Schittek B. Tolson J. . (2006). Cathepsin D is present in human eccrine sweat and involved in the postsecretory processing of the antimicrobial peptide DCD-1L. J. Biol. Chem. 281, 5406–5415. 10.1074/jbc.M504670200 16354654

[B17] Bajo-Grañeras R. Crespo-Sanjuan J. García-Centeno R. M. Garrote-Adrados J. A. Gutierrez G. García-Tejeiro M. . (2013). Expression and potential role of apolipoprotein D on the death-survival balance of human colorectal cancer cells under oxidative stress conditions. Int. J. Colorectal Dis. 28, 751–766. 10.1007/s00384-012-1616-2 23296401

[B18] Bajo-Grañeras R. Ganfornina M. D. Martín-Tejedor E. Sanchez D. (2011a). Apolipoprotein D mediates autocrine protection of astrocytes and controls their reactivity level, contributing to the functional maintenance of paraquat-challenged dopaminergic systems. Glia 59, 1551–1566. 10.1002/glia.21200 21688324

[B19] Bajo-Grañeras R. Sanchez D. Gutierrez G. González C. Do Carmo S. Rassart E. . (2011b). Apolipoprotein D alters the early transcriptional response to oxidative stress in the adult cerebellum. J. Neurochem. 117, 949–960. 10.1111/j.1471-4159.2011.07266.x 21463325

[B20] Baker W. A. Hitman G. A. Hawrami K. McCarthy M. I. Riikonen A. Tuomilehto-Wolf E. . (1994). Apolipoprotein D gene polymorphism: a new genetic marker for type 2 diabetic subjects in Nauru and south India. Diabet. Med. 11, 947–952. 10.1111/j.1464-5491.1994.tb00252.x 7895459

[B21] Balbín M. Freije J. M. Fueyo A. Sánchez L. M. López-Otín C. (1990). Apolipoprotein D is the major protein component in cyst fluid from women with human breast gross cystic disease. Biochem. J. 271, 803–807. 10.1042/bj2710803 2244881 PMC1149635

[B22] Baris O. Savagner F. Nasser V. Loriod B. Granjeaud S. Guyetant S. . (2004). Transcriptional profiling reveals coordinated up-regulation of oxidative metabolism genes in thyroid oncocytic tumors. J. Clin. Endocrinol. Metab. 89, 994–1005. 10.1210/jc.2003-031238 14764826

[B23] Befort K. Filliol D. Darcq E. Ghate A. Matifas A. Lardenois A. . (2008). Gene expression is altered in the lateral hypothalamus upon activation of the mu opioid receptor. Ann. N. Y. Acad. Sci. 1129, 175–184. 10.1196/annals.1417.028 18591478

[B24] Belloir B. Kövari E. Surini-Demiri M. Savioz A. (2001). Altered apolipoprotein D expression in the brain of patients with Alzheimer disease. J. Neurosci. Res. 64, 61–69. 10.1002/jnr.1054 11276052

[B25] Bhatia S. Jenner A. M. Li H. Ruberu K. Spiro A. S. Shepherd C. E. . (2013). Increased apolipoprotein D dimer formation in Alzheimer's disease hippocampus is associated with lipid conjugated diene levels. J. Alzheimers Dis. 35, 475–486. 10.3233/JAD-122278 23455990

[B26] Bhatia S. Kim W. S. Shepherd C. E. Halliday G. M. (2019). Apolipoprotein D upregulation in Alzheimer's disease but not frontotemporal dementia. J. Mol. Neurosci. 67, 125–132. 10.1007/s12031-018-1217-9 30467822 PMC6344390

[B27] Bhatia S. Knoch B. Wong J. Kim W. S. Else P. L. Oakley A. J. . (2012). Selective reduction of hydroperoxyeicosatetraenoic acids to their hydroxy derivatives by apolipoprotein D: implications for lipid antioxidant activity and Alzheimer's disease. Biochem. J. 442, 713–721. 10.1042/BJ20111166 22150111

[B28] Bianchi-Frias D. Pritchard C. Mecham B. H. Coleman I. M. Nelson P. S. (2007). Genetic background influences murine prostate gene expression: implications for cancer phenotypes. Genome Biol. 8:R117. 10.1186/gb-2007-8-6-r117 17577413 PMC2394769

[B29] Bianchi-Frias D. Vakar-Lopez F. Coleman I. M. Plymate S. R. Reed M. J. Nelson P. S. (2010). The effects of aging on the molecular and cellular composition of the prostate microenvironment. PLoS ONE 5:e12501. 10.1371/journal.pone.0012501 20824135 PMC2931699

[B30] Bidinotto L. T. de Cicco R. L. Vanegas J. E. Santucci-Pereira J. Vanden Heuvel J. P. Washington S. . (2012). Fish oil alters tamoxifen-modulated expression of mRNAs that encode genes related to differentiation, proliferation, metastasis, and immune response in rat mammary tumors. Nutr. Cancer 64, 991–999. 10.1080/01635581.2012.712736 23061905 PMC3595161

[B31] Blais Y. Sugimoto K. Carriere M. C. Haagensen D. E. Labrie F. Simard J. (1994). Potent stimulatory effect of interleukin-1 alpha on apolipoprotein D and gross cystic disease fluid protein-15 expression in human breast-cancer cells. Int. J. Cancer 59, 400–407. 10.1002/ijc.2910590319 7927949

[B32] Blais Y. Sugimoto K. Carrière M. C. Haagensen D. E. Labrie F. Simard J. (1995). Interleukin-6 inhibits the potent stimulatory action of androgens, glucocorticoids and interleukin-1 alpha on apolipoprotein D and GCDFP-15 expression in human breast cancer cells. Int. J. Cancer 62, 732–737. 10.1002/ijc.2910620614 7558422

[B33] Blanco-Vaca F. Gaubatz J. W. Bren N. Kottke B. A. Morrisett J. D. Guevara J. (1994). Identification and quantification of apolipoproteins in addition to apo[a] and apo B-100 in human lipoprotein[a]. Chem. Phys. Lipids 67–68, 35–42. 10.1016/0009-3084(94)90122-8 8187234

[B34] Blanco-Vaca F. Pownall H. J. (1993). Disulfide linked dimers of apolipoprotein D in urine. Electrophoresis 14, 1086–1087. 10.1002/elps.11501401175 8125062

[B35] Blanco-Vaca F. Via D. P. Yang C. Y. Massey J. B. Pownall H. J. (1992). Characterization of disulfide-linked heterodimers containing apolipoprotein D in human plasma lipoproteins. J. Lipid Res. 33, 1785–1796. 10.1016/S0022-2275(20)41336-7 1479288

[B36] Boer S. Sanchez D. Reinieren I. van den Boom T. Udawela M. Scarr E. . (2010). Decreased kainate receptors in the hippocampus of apolipoprotein D knockout mice. Prog. Neuropsychopharmacol. Biol. Psychiatry 34, 271–278. 10.1016/j.pnpbp.2009.11.016 19963028

[B37] Bojanovski D. Alaupovic P. McConathy W. J. Kelly J. L. (1980). Isolation and partial characterization of apolipoprotein D and lipoprotein D from baboon plasma. FEBS Lett. 112, 251–254. 10.1016/0014-5793(80)80191-8 6768594

[B38] Borghini I. Barja F. Pometta D. James R. W. (1995). Characterization of subpopulations of lipoprotein particles isolated from human cerebrospinal fluid. Biochim. Biophys. Acta 1255, 192–200. 10.1016/0005-2760(94)00232-N 7696334

[B39] Böttcher A. Schlosser J. Kronenberg F. Dieplinger H. Knipping G. Lackner K. J. . (2000). Preparative free-solution isotachophoresis for separation of human plasma lipoproteins: apolipoprotein and lipid composition of HDL subfractions. J. Lipid Res. 41, 905–915. 10.1016/S0022-2275(20)32032-0 10828082

[B40] Bouma M. E. de Bandt J. P. Ayrault-Jarrier M. Burdin J. Verthier N. Raisonnier A. (1988). Immunoperoxidase localization of apolipoprotein D in human enterocytes and hepatocytes. Scand. J. Gastroenterol. 23, 477–483. 10.3109/00365528809093897 3289112

[B41] Boyles J. K. Notterpek L. M. Anderson L. J. (1990a). Accumulation of apolipoproteins in the regenerating and remyelinating mammalian peripheral nerve. Identification of apolipoprotein D, apolipoprotein A-IV, apolipoprotein E, and apolipoprotein A-I. J. Biol. Chem. 265, 17805–17815. 2120218

[B42] Boyles J. K. Notterpek L. M. Wardell M. R. Rall S. C. (1990b). Identification, characterization, and tissue distribution of apolipoprotein D in the rat. J. Lipid Res. 31, 2243–2256. 2090718

[B43] Braesch-Andersen S. Beckman L. Paulie S. Kumagai-Braesch M. (2014). ApoD mediates binding of HDL to LDL and to growing T24 carcinoma. PLoS ONE 9:e115180. 10.1371/journal.pone.0115180 25513803 PMC4267786

[B44] Brahimaj A. Ligthart S. Ikram M. A. Hofman A. Franco O. H. Sijbrands E. J. G. . (2017). Serum levels of apolipoproteins and incident type 2 diabetes: a prospective cohort study. Diabetes Care 40, 346–351. 10.2337/dc16-1295 28031419

[B45] Breustedt D. A. Schönfeld D. L. Skerra A. (2006). Comparative ligand-binding analysis of ten human lipocalins. Biochim. Biophys. Acta 1764, 161–173. 10.1016/j.bbapap.2005.12.006 16461020

[B46] Bujalska I. J. Quinkler M. Tomlinson J. W. Montague C. T. Smith D. M. Stewart P. M. (2006). Expression profiling of 11beta-hydroxysteroid dehydrogenase type-1 and glucocorticoid-target genes in subcutaneous and omental human preadipocytes. J. Mol. Endocrinol. 37, 327–340. 10.1677/jme.1.02048 17032748

[B47] Camato R. Marcel Y. L. Milne R. W. Lussier-Cacan S. Weech P. K. (1989). Protein polymorphism of a human plasma apolipoprotein D antigenic epitope. J. Lipid Res. 30, 865–875. 10.1016/S0022-2275(20)38304-8 2477480

[B48] Caparosa E. M. Sedgewick A. J. Zenonos G. Zhao Y. Carlisle D. L. Stefaneanu L. . (2019). Regional molecular signature of the symptomatic atherosclerotic carotid plaque. Neurosurgery 85, E284–E293. 10.1093/neuros/nyy470 30335165 PMC12311974

[B49] Carnes M. U. Allingham R. R. Ashley-Koch A. Hauser M. A. (2018). Transcriptome analysis of adult and fetal trabecular meshwork, cornea, and ciliary body tissues by RNA sequencing. Exp. Eye Res. 167, 91–99. 10.1016/j.exer.2016.11.021 27914989

[B50] Carreño G. Del Casar J. M. Corte M. D. González L. O. Bongera M. Merino A. M. . (2007). Local recurrence after mastectomy for breast cancer: analysis of clinicopathological, biological and prognostic characteristics. Breast Cancer Res. Treat. 102, 61–73. 10.1007/s10549-006-9310-0 16850244

[B51] Cha M.-H. Kim I.-C. Lee B.-H. Yoon Y. (2006). Baicalein inhibits adipocyte differentiation by enhancing COX-2 expression. J. Med. Food 9, 145–153. 10.1089/jmf.2006.9.145 16822198

[B52] Chen H. Li Y. Du J. Cao Y. Li X. (2011). Increased JNK1 activity contributes to the upregulation of ApoD in the apocrine secretory gland cells from axillary osmidrosis. Mol. Cell. Biochem. 354, 311–316. 10.1007/s11010-011-0830-5 21526344

[B53] Chen H. Yang G. Li Y. Li X. Du J. (2013). Expression of apolipoprotein D and androgen receptor in axillary osmidrosis and its molecular mechanism. Int. J. Clin. Exp. Med. 6, 497–503. 23936587 PMC3731180

[B54] Chen J. Heerdt B. G. Augenlicht L. H. (1995). Presence and instability of repetitive elements in sequences the altered expression of which characterizes risk for colonic cancer. Cancer Res. 55, 174–180. 7805030

[B55] Chen Y. Jia L. Wei C. Wang F. Lv H. Jia J. (2008). Association between polymorphisms in the apolipoprotein D gene and sporadic Alzheimer's disease. Brain Res. 1233, 196–202. 10.1016/j.brainres.2008.07.018 18671953

[B56] Chen Y.-W. Gregory C. M. Scarborough M. T. Shi R. Walter G. A. Vandenborne K. (2007). Transcriptional pathways associated with skeletal muscle disuse atrophy in humans. Physiol. Genomics 31, 510–520. 10.1152/physiolgenomics.00115.2006 17804603

[B57] Cheow E. S. H. Cheng W. C. Lee C. N. de Kleijn D. Sorokin V. Sze S. K. (2016). Plasma-derived extracellular vesicles contain predictive biomarkers and potential therapeutic targets for Myocardial Ischemic (MI) injury. Mol. Cell Proteomics 15, 2628–2640. 10.1074/mcp.M115.055731 27234505 PMC4974341

[B58] Cheung M. C. Mendez A. J. Wolf A. C. Knopp R. H. (1993). Characterization of apolipoprotein A-I- and A-II-containing lipoproteins in a new case of high density lipoprotein deficiency resembling Tangier disease and their effects on intracellular cholesterol efflux. J. Clin. Invest. 91, 522–529. 10.1172/JCI116231 8432861 PMC287973

[B59] Chia W.-J. Dawe G. S. Ong W.-Y. (2011). Expression and localization of the iron-siderophore binding protein lipocalin 2 in the normal rat brain and after kainate-induced excitotoxicity. Neurochem. Int. 59, 591–599. 10.1016/j.neuint.2011.04.007 21683107

[B60] Clements J. A. Rohde P. Allen V. Hyland V. J. Samaratunga M. L. Tilley W. D. . (1999). Molecular detection of prostate cells in ejaculate and urethral washings in men with suspected prostate cancer. J. Urol. 161, 1337–1343. 10.1016/S0022-5347(01)61680-X 10081904

[B61] Cofer S. Ross S. R. (1996). The murine gene encoding apolipoprotein D exhibits a unique expression pattern as compared to other species. Gene 171, 261–263. 10.1016/0378-1119(96)00099-6 8666283

[B62] Colin C. Baeza N. Bartoli C. Fina F. Eudes N. Nanni I. . (2006). Identification of genes differentially expressed in glioblastoma versus pilocytic astrocytoma using Suppression Subtractive Hybridization. Oncogene 25, 2818–2826. 10.1038/sj.onc.1209305 16314830

[B63] Collins-Racie L. A. Yang Z. Arai M. Li N. Majumdar M. K. Nagpal S. . (2009). Global analysis of nuclear receptor expression and dysregulation in human osteoarthritic articular cartilage: reduced LXR signaling contributes to catabolic metabolism typical of osteoarthritis. Osteoarthr. Cartil. 17, 832–842. 10.1016/j.joca.2008.12.011 19217805

[B64] Contreras-Duarte S. Chen P. Andía M. Uribe S. Irarrázaval P. Kopp S.. (2018). Attenuation of atherogenic apo B-48-dependent hyperlipidemia and high density lipoprotein remodeling induced by vitamin C and E combination and their beneficial effect on lethal ischemic heart disease in mice. Biol. Res. 51:34. 10.1186/s40659-018-0183-6 30219096 PMC6138920

[B65] Csosz É. Emri G. Kalló G. Tsaprailis G. Tozsér J. (2015). Highly abundant defense proteins in human sweat as revealed by targeted proteomics and label-free quantification mass spectrometry. J. Eur. Acad. Dermatol. Venereol. 29, 2024–2031. 10.1111/jdv.13221 26307449 PMC4583350

[B66] Curry M. D. McConathy W. J. Alaupovic P. (1977). Quantitative determination of human apolipoprotein D by electroimmunoassay and radial immunodiffusion. Biochim. Biophys. Acta 491, 232–241. 10.1016/0005-2795(77)90059-9 191086

[B67] Cury S. S. de Moraes D. Freire P. P. de Oliveira G. Marques D. V. P. Fernandez G. J. . (2019). Tumor transcriptome reveals high expression of IL-8 in non-small cell lung cancer patients with low pectoralis muscle area and reduced survival. Cancers 11:1251. 10.3390/cancers11091251 31455042 PMC6769884

[B68] Dandoy-Dron F. Guillo F. Benboudjema L. Deslys J. P. Lasmézas C. Dormont D. . (1998). Gene expression in scrapie. Cloning of a new scrapie-responsive gene and the identification of increased levels of seven other mRNA transcripts. J. Biol. Chem. 273, 7691–7697. 10.1074/jbc.273.13.7691 9516475

[B69] Dassati S. Schweigreiter R. Buechner S. Waldner A. (2020). Celecoxib promotes survival and upregulates the expression of neuroprotective marker genes in two different *in vitro* models of Parkinson's disease. Neuropharmacology 194:108378. 10.1016/j.neuropharm.2020.108378 33160981

[B70] de Magalhães J. P. Curado J. Church G. M. (2009). Meta-analysis of age-related gene expression profiles identifies common signatures of aging. Bioinformatics 25, 875–881. 10.1093/bioinformatics/btp073 19189975 PMC2732303

[B71] Dean B. Digney A. Sundram S. Thomas E. Scarr E. (2008). Plasma apolipoprotein E is decreased in schizophrenia spectrum and bipolar disorder. Psychiatry Res. 158, 75–78. 10.1016/j.psychres.2007.05.008 18096247

[B72] Del Caño-Espinel M. del. (2014). Relación de la apolipoproteína D y sus homólogos en Drosophila con las membranas biológicas: Estudio de su función en diferentes procesos celulares y de su localización y efectos sobre las balsas lipídicas (Ph.D. thesis). Valladolid, Spain. 10.35376/10324/7752

[B73] Del Signore A. De Sanctis V. Di Mauro E. Negri R. Perrone-Capano C. Paggi P. (2006). Gene expression pathways induced by axotomy and decentralization of rat superior cervical ganglion neurons. Eur. J. Neurosci. 23, 65–74. 10.1111/j.1460-9568.2005.04520.x 16420416

[B74] del Valle E. Navarro A. Astudillo A. Tolivia J. (2003). Apolipoprotein D expression in human brain reactive astrocytes. J. Histochem. Cytochem. 51, 1285–1290. 10.1177/002215540305101005 14500696

[B75] Del Valle E. Navarro A. Martínez-Pinilla E. Torices S. Tolivia J. (2016). Apo J and Apo D: complementary or antagonistic roles in Alzheimer's disease? J. Alzheimers Dis. 53, 639–650. 10.3233/JAD-160032 27197790

[B76] Delattre C. Winstall E. Lessard C. Donovan M. Simonetti L. Minondo A.-M. . (2012). Proteomic analysis identifies new biomarkers for postmenopausal and dry skin. Exp. Dermatol. 21, 205–210. 10.1111/j.1600-0625.2011.01434.x 22379966

[B77] Deng P. Zeng J. Li J. Feng W. Chen J. Zeng Y. (2018). Screening of serum protein markers for avascular osteonecrosis of femoral head differentially expressed after treatment with yuanshi shengmai chenggu tablets. Biomed. Res. Int. 2018:5692735. 10.1155/2018/5692735 29750162 PMC5884301

[B78] Denis M. Landry Y. D. Zha X. (2008). ATP-binding cassette A1-mediated lipidation of apolipoprotein A-I occurs at the plasma membrane and not in the endocytic compartments. J. Biol. Chem. 283, 16178–16186. 10.1074/jbc.M709597200 18385134 PMC3259641

[B79] Desai P. P. Bunker C. H. Ukoli F. A. M. Kamboh M. I. (2002). Genetic variation in the apolipoprotein D gene among African blacks and its significance in lipid metabolism. Atherosclerosis 163, 329–338. 10.1016/S0021-9150(02)00012-6 12052480

[B80] Desai P. P. Hendrie H. C. Evans R. M. Murrell J. R. DeKosky S. T. Kamboh M. I. (2003). Genetic variation in apolipoprotein D affects the risk of Alzheimer disease in African-Americans. Am. J. Med. Genet. B Neuropsychiatr. Genet. 116B, 98–101. 10.1002/ajmg.b.10798 12497622

[B81] Desai P. P. Ikonomovic M. D. Abrahamson E. E. Hamilton R. L. Isanski B. A. Hope C. E. . (2005). Apolipoprotein D is a component of compact but not diffuse amyloid-beta plaques in Alzheimer's disease temporal cortex. Neurobiol. Dis. 20, 574–582. 10.1016/j.nbd.2005.04.012 15916898

[B82] Desmarais F. Bergeron K.-F. Lacaille M. Lemieux I. Bergeron J. Biron S. . (2018). High ApoD protein level in the round ligament fat depot of severely obese women is associated with an improved inflammatory profile. Endocrine 61, 248–257. 10.1007/s12020-018-1621-5 29869155

[B83] Desmarais F. Bergeron K.-F. Rassart E. Mounier C. (2019). Apolipoprotein D overexpression alters hepatic prostaglandin and omega fatty acid metabolism during the development of a non-inflammatory hepatic steatosis. Biochim. Biophys. Acta Mol. Cell. Biol. Lipids 1864, 522–531. 10.1016/j.bbalip.2019.01.001 30630053

[B84] Diez-Hermano S. Ganfornina M. D. Skerra A. Gutierrez G. Sanchez D. (2021). An evolutionary perspective of the Lipocalin protein family. Front. Physiol. 12:718983. 10.3389/fphys.2021.718983 34497539 PMC8420045

[B85] Diez-Hermano S. Mejias A. Sanchez D. Gutierrez G. Ganfornina M. D. (2020). Control of the neuroprotective Lipocalin Apolipoprotein D expression by alternative promoter regions and differentially expressed mRNA 5' UTR variants. PLoS ONE 15:e0234857. 10.1371/journal.pone.0234857 32559215 PMC7304576

[B86] Díez-Itza I. Vizoso F. Merino A. M. Sánchez L. M. Tolivia J. Fernández J. . (1994). Expression and prognostic significance of apolipoprotein D in breast cancer. Am. J. Pathol. 144, 310–320. 8311115 PMC1887137

[B87] Dilley W. G. Haagensen D. E. Cox C. E. Wells S. A. (1990). Immunologic and steroid binding properties of the GCDFP-24 protein isolated from human breast gross cystic disease fluid. Breast Cancer Res. Treat. 16, 253–260. 10.1007/BF01806333 2085676

[B88] Do Carmo S. Forest J.-C. Giguère Y. Masse A. Lafond J. Rassart E. (2009a). Modulation of Apolipoprotein D levels in human pregnancy and association with gestational weight gain. Reprod. Biol. Endocrinol. 7:92. 10.1186/1477-7827-7-92 19723339 PMC3224896

[B89] Do Carmo S. Fournier D. Mounier C. Rassart E. (2009b). Human apolipoprotein D overexpression in transgenic mice induces insulin resistance and alters lipid metabolism. Am. J. Physiol. Endocrinol. Metab. 296, E802–E811. 10.1152/ajpendo.90725.2008 19176353

[B90] Do Carmo S. Jacomy H. Talbot P. J. Rassart E. (2008). Neuroprotective effect of apolipoprotein D against human coronavirus OC43-induced encephalitis in mice. J. Neurosci. 28, 10330–10338. 10.1523/JNEUROSCI.2644-08.2008 18842892 PMC6671015

[B91] Do Carmo S. Levros L.-C. Rassart E. (2007). Modulation of apolipoprotein D expression and translocation under specific stress conditions. Biochim. Biophys. Acta 1773, 954–969. 10.1016/j.bbamcr.2007.03.007 17477983

[B92] Do Carmo S. Séguin D. Milne R. Rassart E. (2002). Modulation of apolipoprotein D and apolipoprotein E mRNA expression by growth arrest and identification of key elements in the promoter. J. Biol. Chem. 277, 5514–5523. 10.1074/jbc.M105057200 11711530

[B93] Doane A. S. Danso M. Lal P. Donaton M. Zhang L. Hudis C. . (2006). An estrogen receptor-negative breast cancer subset characterized by a hormonally regulated transcriptional program and response to androgen. Oncogene 25, 3994–4008. 10.1038/sj.onc.1209415 16491124

[B94] Drayna D. Fielding C. McLean J. Baer B. Castro G. Chen E. . (1986). Cloning and expression of human apolipoprotein D cDNA. J. Biol. Chem. 261, 16535–16539. 10.1016/S0021-9258(18)66599-8 3453108

[B95] Drayna D. Scott J. D. Lawn R. (1987). Multiple RFLPs at the human apolipoprotein D (APOD) locus. Nucleic Acids Res. 15:9617. 10.1093/nar/15.22.9617 2891117 PMC306509

[B96] Edlow A. G. Vora N. L. Hui L. Wick H. C. Cowan J. M. Bianchi D. W. (2014). Maternal obesity affects fetal neurodevelopmental and metabolic gene expression: a pilot study. PLoS ONE 9:e88661. 10.1371/journal.pone.0088661 24558408 PMC3928248

[B97] Eichinger A. Nasreen A. Kim H. J. Skerra A. (2007). Structural insight into the dual ligand specificity and mode of high density lipoprotein association of apolipoprotein D. J. Biol. Chem. 282, 31068–31075. 10.1074/jbc.M703552200 17699160

[B98] Eichner J. E. Kuller L. H. Ferrell R. E. Kamboh M. I. (1989). Phenotypic effects of apolipoprotein structural variation on lipid profiles. IV. Apolipoprotein polymorphisms in a small group of black women from the healthy women study. Genet. Epidemiol. 6, 681–689. 10.1002/gepi.1370060605 2606341

[B99] Eigeliene N. Elo T. Linhala M. Hurme S. Erkkola R. Härkönen P. (2012). Androgens inhibit the stimulatory action of 17β-estradiol on normal human breast tissue in explant cultures. J. Clin. Endocrinol. Metab. 97, E1116–E1127. 10.1210/jc.2011-3228 22535971

[B100] El-Darzi N. Mast N. Petrov A. M. Dao T. Astafev A. A. Saadane A. . (2020). Studies of ApoD-/- and ApoD-/-ApoE-/- mice uncover the APOD significance for retinal metabolism, function, and status of chorioretinal blood vessels. Cell. Mol. Life Sci. 78, 963–983. 10.1007/s00018-020-03546-3 32440710 PMC7679289

[B101] Escribano J. Ortego J. Coca-Prados M. (1995). Isolation and characterization of cell-specific cDNA clones from a subtractive library of the ocular ciliary body of a single normal human donor: transcription and synthesis of plasma proteins. J. Biochem. 118, 921–931. 10.1093/jb/118.5.921 8749308

[B102] Fielding P. E. Fielding C. J. (1980). A cholesteryl ester transfer complex in human plasma. Proc. Natl. Acad. Sci. U.S.A. 77, 3327–3330. 6774335 10.1073/pnas.77.6.3327 PMC349608

[B103] Flatscher-Bader T. Wilce P. A. (2006). Chronic smoking and alcoholism change expression of selective genes in the human prefrontal cortex. Alcohol. Clin. Exp. Res. 30, 908–915. 10.1111/j.1530-0277.2006.00106.x 16634861

[B104] Flores R. Jin X. Chang J. Zhang C. Cogan D. G. Schaefer E. J. . (2019). LCAT, ApoD, and ApoA1 expression and review of cholesterol deposition in the cornea. Biomolecules 9:785. 10.3390/biom9120785 31779197 PMC6995527

[B105] Fontaine J.-F. Mirebeau-Prunier D. Raharijaona M. Franc B. Triau S. Rodien P. . (2009). Increasing the number of thyroid lesions classes in microarray analysis improves the relevance of diagnostic markers. PLoS ONE 4:e7632. 10.1371/journal.pone.0007632 19893615 PMC2764086

[B106] Forster C. S. Haffey W. D. Bennett M. Greis K. D. Devarajan P. (2019). Identification of urinary CD44 and prosaposin as specific biomarkers of urinary tract infections in children with neurogenic bladders. Biomark Insights 14, 1–7. 10.1177/1177271919835570 30906192 PMC6421595

[B107] Francone O. L. Gurakar A. Fielding C. (1989). Distribution and functions of lecithin:cholesterol acyltransferase and cholesteryl ester transfer protein in plasma lipoproteins. Evidence for a functional unit containing these activities together with apolipoproteins A-I and D that catalyzes the esterification and transfer of cell-derived cholesterol. J. Biol. Chem. 264, 7066–7072. 10.1016/S0021-9258(18)83541-4 2496125

[B108] Franz G. Reindl M. Patel S. C. Beer R. Unterrichter I. Berger T. . (1999). Increased expression of apolipoprotein D following experimental traumatic brain injury. J. Neurochem. 73, 1615–1625. 10.1046/j.1471-4159.1999.0731615.x 10501208

[B109] Fujita T. Iwata T. Shiba H. Igarashi A. Hirata R. Takeda K. . (2007). Identification of marker genes distinguishing human periodontal ligament cells from human mesenchymal stem cells and human gingival fibroblasts. J. Periodont. Res. 42, 283–286. 10.1111/j.1600-0765.2006.00944.x 17451549

[B110] Galamb O. Sipos F. Solymosi N. Spisák S. Krenács T. Tóth K. . (2008). Diagnostic mRNA expression patterns of inflamed, benign, and malignant colorectal biopsy specimen and their correlation with peripheral blood results. Cancer Epidemiol. Biomarkers Prev. 17, 2835–2845. 10.1158/1055-9965.EPI-08-0231 18843029

[B111] Ganfornina M. D. Do Carmo S. Lora J. M. Torres-Schumann S. Vogel M. Allhorn M. . (2008). Apolipoprotein D is involved in the mechanisms regulating protection from oxidative stress. Aging Cell 7, 506–515. 10.1111/j.1474-9726.2008.00395.x 18419796 PMC2574913

[B112] Ganfornina M. D. Do Carmo S. Martínez E. Tolivia J. Navarro A. Rassart E. . (2010). ApoD, a glia-derived apolipoprotein, is required for peripheral nerve functional integrity and a timely response to injury. Glia 58, 1320–1334. 10.1002/glia.21010 20607718 PMC7165554

[B113] Ganfornina M. D. Gutiérrez G. Bastiani M.S. D. (2000). A phylogenetic analysis of the lipocalin protein family. Mol. Biol. Evol. 17, 114–126. 10.1093/oxfordjournals.molbev.a026224 10666711

[B114] Ganfornina M. D. Sánchez D. Bastiani M. J. (1995). Lazarillo, a new GPI-linked surface lipocalin, is restricted to a subset of neurons in the grasshopper embryo. Development 121, 123–134. 10.1242/dev.121.1.123 7867494

[B115] Ganfornina M. D. Sánchez D. Pagano A. Tonachini L. Descalzi-Cancedda F. Martínez S. (2005). Molecular characterization and developmental expression pattern of the chicken apolipoprotein D gene: implications for the evolution of vertebrate lipocalins. Dev. Dyn. 232, 191–199. 10.1002/dvdy.20193 15580625

[B116] Gao G.-Q. Song L.-S. Tong B. Li G.-P. (2016). Expression levels of GSTA2 and APOD genes might be associated with carotenoid coloration in golden pheasant (*Chrysolophus pictus*) plumage. Zool. Res. 37, 144–150. 10.13918/j.issn.2095-8137.2016.3.144 27265652 PMC4914577

[B117] García-Mateo N. Ganfornina M. D. Montero O. Gijón M. A. Murphy R. C. Sanchez D. (2014). Schwann cell-derived Apolipoprotein D controls the dynamics of post-injury myelin recognition and degradation. Front. Cell Neurosci 8:374. 10.3389/fncel.2014.00374 25426024 PMC4227524

[B118] García-Mateo N. Pascua-Maestro R. Pérez-Castellanos A. Lillo C. Sanchez D. Ganfornina M. D. (2018). Myelin extracellular leaflet compaction requires apolipoprotein D membrane management to optimize lysosomal-dependent recycling and glycocalyx removal. Glia 66, 670–687. 10.1002/glia.23274 29222871

[B119] Geng Y.-J. Li T. Wen L. Gao H. Yuan J. Liu W.-C. . (2020). Percutaneous interstitial nd:YAG laser therapy for axillary osmidrosis. Lasers Surg. Med. 52, 639–646. 10.1002/lsm.23187 31736126

[B120] Germeyer A. Capp E. Schlicksupp F. Jauckus J. von Rango U. von Wolff M. . (2013). Cell-type specific expression and regulation of apolipoprotein D and E in human endometrium. Eur. J. Obstet. Gynecol. Reprod. Biol. 170, 487–491. 10.1016/j.ejogrb.2013.06.043 23895740

[B121] Gillen C. Gleichmann M. Spreyer P. Müller H. W. (1995). Differentially expressed genes after peripheral nerve injury. J. Neurosci. Res. 42, 159–171. 10.1002/jnr.490420203 8568916

[B122] Glöckner F. Ohm T. G. (2003). Hippocampal apolipoprotein D level depends on Braak stage and APOE genotype. Neuroscience 122, 103–110. 10.1016/S0306-4522(03)00529-3 14596852

[B123] Goessling W. Zucker S. D. (2000). Role of apolipoprotein D in the transport of bilirubin in plasma. Am. J. Physiol. Gastrointest. Liver Physiol. 279, G356–365. 10.1152/ajpgi.2000.279.2.G356 10915645

[B124] Gonzalez L. O. Corte M. D. Junquera S. Bongera M. Rodriguez J. C. Vizoso F. J. (2007). Expression of androgen receptor and two androgen-induced proteins (apolipoprotein D and pepsinogen C) in ductal carcinoma *in situ* of the breast. Histopathology 50, 866–874. 10.1111/j.1365-2559.2007.02687.x 17543076

[B125] Gottsch J. D. Bowers A. L. Margulies E. H. Seitzman G. D. Kim S. W. Saha S. . (2003). Serial analysis of gene expression in the corneal endothelium of Fuchs' dystrophy. Invest. Ophthalmol. Vis. Sci. 44, 594–599. 10.1167/iovs.02-0300 12556388

[B126] Greenberg M. E. Li X.-M. Gugiu B. G. Gu X. Qin J. Salomon R. G. . (2008). The lipid whisker model of the structure of oxidized cell membranes. J. Biol. Chem. 283, 2385–2396. 10.1074/jbc.M707348200 18045864

[B127] Gutmann D. H. Hedrick N. M. Li J. Nagarajan R. Perry A. Watson M. A. (2002). Comparative gene expression profile analysis of neurofibromatosis 1-associated and sporadic pilocytic astrocytomas. Cancer Res. 62, 2085–2091. 11929829

[B128] Guven G. Vurgun E. Bilgic B. Hanagasi H. Gurvit H. Ozer E. . (2019). Association between selected cholesterol-related gene polymorphisms and Alzheimer's disease in a Turkish cohort. Mol. Biol. Rep. 46, 1701–1707. 10.1007/s11033-019-04619-8 30684189

[B129] Haagensen D. E. Jr. Mazoujian G. Dilley W. G. Pedersen C. E. Kister S. J. Wells S. A. Jr. (1979). Breast gross cystic disease fluid analysis. i. isolation and radioimmunoassay for a major component protein. JNCI 62, 239–247. 283260

[B130] Haffner S. M. Applebaum-Bowden D. Wahl P. W. Hoover J. J. Warnick G. R. Albers J. J. . (1985). Epidemiological correlates of high density lipoprotein subfractions, apolipoproteins A-I, A-II, and D, and lecithin cholesterol acyltransferase. Effects of smoking, alcohol, and adiposity. Arteriosclerosis 5, 169–177. 10.1161/01.ATV.5.2.169 3919701

[B131] Hall R. E. Aspinall J. O. Horsfall D. J. Birrell S. N. Bentel J. M. Sutherland R. L. . (1996). Expression of the androgen receptor and an androgen-responsive protein, apolipoprotein D, in human breast cancer. Br. J. Cancer 74, 1175–1180. 10.1038/bjc.1996.513 8883401 PMC2075941

[B132] Hall R. E. Horsfall D. J. Stahl J. Vivekanandan S. Ricciardelli C. Stapleton A. M. F. . (2004). Apolipoprotein-D: a novel cellular marker for HGPIN and prostate cancer. Prostate 58, 103–108. 10.1002/pros.10343 14716735

[B133] Hansen L. Gaster M. Oakeley E. J. Brusgaard K. Damsgaard Nielsen E.-M. Beck-Nielsen H. . (2004). Expression profiling of insulin action in human myotubes: induction of inflammatory and pro-angiogenic pathways in relationship with glycogen synthesis and type 2 diabetes. Biochem. Biophys. Res. Commun. 323, 685–695. 10.1016/j.bbrc.2004.08.146 15369805

[B134] Hansen T. Hemmingsen R. P. Wang A. G. Olsen L. Timm S. Søeby K. . (2006). Apolipoprotein D is associated with long-term outcome in patients with schizophrenia. Pharmacogenomics J. 6, 120–125. 10.1038/sj.tpj.6500350 16402085

[B135] Hardardóttir I. Sipe J. Moser A. H. Fielding C. J. Feingold K. R. Grünfeld C. (1997). LPS and cytokines regulate extra hepatic mRNA levels of apolipoproteins during the acute phase response in Syrian hamsters. Biochim. Biophys. Acta 1344, 210–220. 10.1016/S0005-2760(96)00143-9 9059511

[B136] Harding C. Osundeko O. Tetlow L. Faragher E. B. Howell A. Bundred N. J. (2000). Hormonally-regulated proteins in breast secretions are markers of target organ sensitivity. Br. J. Cancer 82, 354–360. 10.1054/bjoc.1999.0926 10646888 PMC2363294

[B137] Hargreaves D. F. Potten C. S. Harding C. Shaw L. E. Morton M. S. Roberts S. A. . (1999). Two-week dietary soy supplementation has an estrogenic effect on normal premenopausal breast. J. Clin. Endocrinol. Metab. 84, 4017–4024. 10.1210/jc.84.11.4017 10566643

[B138] Hatzirodos N. Hummitzsch K. Irving-Rodgers H. F. Rodgers R. J. (2015). Transcriptome comparisons identify new cell markers for theca interna and granulosa cells from small and large antral ovarian follicles. PLoS ONE 10:e0119800. 10.1371/journal.pone.0119800 25775029 PMC4361622

[B139] He X. Jittiwat J. Kim J.-H. Jenner A. M. Farooqui A. A. Patel S. C. . (2009). Apolipoprotein D modulates F2-isoprostane and 7-ketocholesterol formation and has a neuroprotective effect on organotypic hippocampal cultures after kainate-induced excitotoxic injury. Neurosci. Lett. 455, 183–186. 10.1016/j.neulet.2009.03.038 19429117 PMC7117013

[B140] Helisalmi S. Hiltunen M. Vepsäläinen S. Iivonen S. Corder E. H. Lehtovirta M. . (2004). Genetic variation in apolipoprotein D and Alzheimer's disease. J. Neurol. 251, 951–957. 10.1007/s00415-004-0470-8 15316799

[B141] Henri P. Rumeau D. (2021). Ectopic expression of human apolipoprotein D in Arabidopsis plants lacking chloroplastic lipocalin partially rescues sensitivity to drought and oxidative stress. Plant Physiol. Biochem. 158, 265–274. 10.1016/j.plaphy.2020.11.009 33262014

[B142] Herzig K.-H. Leppäluoto J. Jokelainen J. Meugnier E. Pesenti S. Selänne H. . (2017). Low level activity thresholds for changes in NMR biomarkers and genes in high risk subjects for Type 2 diabetes. Sci. Rep. 7:11267. 10.1038/s41598-017-09753-6 28924247 PMC5603534

[B143] Hildebrand M. S. de Silva M. G. Klockars T. Solares C. A. Hirose K. Smith J. D. . (2005). Expression of the carrier protein apolipoprotein D in the mouse inner ear. Hear. Res. 200, 102–114. 10.1016/j.heares.2004.08.018 15668042

[B144] Holmquist L. (1989). Separation of free and apolipoprotein D-associated human plasma lecithin: cholesterol acyltransferase. J. Biochem. Biophys. Methods 19, 93–103. 10.1016/0165-022X(89)90054-7 2809071

[B145] Holmquist L. (1990). Identification and quantification of apolipoprotein D in normal human urine. Electrophoresis 11, 93–94. 10.1002/elps.1150110119 2318194

[B146] Holmquist L. Fredrikson S. Vesterberg O. (1996). A zone immunoelectrophoresis assay method for quantification of apolipoprotein D in human cerebrospinal fluid. J. Biochem. Biophys. Methods 33, 1–8. 10.1016/0165-022X(95)00041-O 8905463

[B147] Holmquist L. Vesterberg O. (1991). Quantification of apolipoprotein D in human urine by zone immunoelectrophoresis assay: a methodological and clinical study. J. Biochem. Biophys. Methods 23, 315–327. 10.1016/0165-022X(91)90007-J 1770201

[B148] Holmquist L. Vesterberg O. Persson B. (1993). Apolipoprotein D and alpha 1-microglobulin in human urine: effect of cadmium exposure. Int. Arch. Occup. Environ. Health 64, 469–472. 10.1007/BF00381093 7683309

[B149] Holzfeind P. Merschak P. Dieplinger H. Redl B. (1995). The human lacrimal gland synthesizes apolipoprotein D mRNA in addition to tear prealbumin mRNA, both species encoding members of the lipocalin superfamily. Exp. Eye Res. 61, 495–500. 10.1016/S0014-4835(05)80145-9 8549691

[B150] Hornig N. C. Demiri J. Rodens P. Murga Penas E. M. Caliebe A. Eckstein A. K. . (2019). Reduced androgen receptor expression in genital skin fibroblasts from patients with 45,X/46,XY mosaicism. J. Clin. Endocrinol. Metab. 104, 4630–4638. 10.1210/jc.2019-00108 31180485

[B151] Hu C. Zhou Y. Liu C. Kang Y. (2018). A novel scoring system for gastric cancer risk assessment based on the expression of three CLIP4 DNA methylation-associated genes. Int. J. Oncol. 53, 633–643. 10.3892/ijo.2018.4433 29901187 PMC6017186

[B152] Hu C. Y. Ong W. Y. Sundaram R. K. Chan C. Patel S. C. (2001). Immunocytochemical localization of apolipoprotein D in oligodendrocyte precursor-like cells, perivascular cells, and pericytes in the human cerebral cortex. J. Neurocytol. 30, 209–218. 10.1023/A:1012797623620 11709627

[B153] Hueging K. Weller R. Doepke M. Vieyres G. Todt D. Wölk B. . (2015). Several human liver cell expressed apolipoproteins complement HCV virus production with varying efficacy conferring differential specific infectivity to released viruses. PLoS ONE 10:e0134529. 10.1371/journal.pone.0134529 26226615 PMC4520612

[B154] Hull-Thompson J. Muffat J. Sanchez D. Walker D. W. Benzer S. Ganfornina M. D. . (2009). Control of metabolic homeostasis by stress signaling is mediated by the lipocalin NLaz. PLoS Genet. 5:e1000460. 10.1371/journal.pgen.1000460 19390610 PMC2667264

[B155] Hummasti S. Laffitte B. A. Watson M. A. Galardi C. Chao L. C. Ramamurthy L. . (2004). Liver X receptors are regulators of adipocyte gene expression but not differentiation: identification of apoD as a direct target. J. Lipid Res. 45, 616–625. 10.1194/jlr.M300312-JLR200 14703507

[B156] Hunter S. Weiss S. Ou C.-Y. Jaye D. Young A. Wilcox J. . (2005b). Apolipoprotein D is down-regulated during malignant transformation of neurofibromas. Hum. Pathol. 36, 987–993. 10.1016/j.humpath.2005.06.018 16153462

[B157] Hunter S. Young A. Olson J. Brat D. J. Bowers G. Wilcox J. N. . (2002). Differential expression between pilocytic and anaplastic astrocytomas: identification of apolipoprotein D as a marker for low-grade, non-infiltrating primary CNS neoplasms. J. Neuropathol. Exp. Neurol. 61, 275–281. 10.1093/jnen/61.3.275 11895042

[B158] Hunter S. B. Varma V. Shehata B. Nolen J. D. L. Cohen C. Olson J. J. . (2005a). Apolipoprotein D expression in primary brain tumors: analysis by quantitative RT-PCR in formalin-fixed, paraffin-embedded tissue. J. Histochem. Cytochem. 53, 963–969. 10.1369/jhc.4A6530.2005 16055749

[B159] Igarashi M. Masunaga Y. Hasegawa Y. Kinjo K. Miyado M. Saitsu H. . (2020). Nonsense-associated altered splicing of MAP3K1 in two siblings with 46,XY disorders of sex development. Sci. Rep. 10:17375. 10.1038/s41598-020-74405-1 33060765 PMC7567082

[B160] Iqbal M. J. Higginbotham A. Chickris N. Bollaert M. Rockway S. Banz W. J. (2008). A combination of CLA-DAG oil modifies the diabetic phenotype in male Zucker diabetic fatty rats. Horm. Metab. Res. 40, 262–268. 10.1055/s-2008-1058063 18548385

[B161] Ishii M. Koike C. Igarashi A. Yamanaka K. Pan H. Higashi Y. . (2005). Molecular markers distinguish bone marrow mesenchymal stem cells from fibroblasts. Biochem. Biophys. Res. Commun. 332, 297–303. 10.1016/j.bbrc.2005.04.118 15896330

[B162] James R. W. Martin B. Pometta D. Grab B. Suenram A. (1986). Apoprotein D in a healthy, male population and in male myocardial infarction patients and their male, first-degree relatives. Atherosclerosis 60, 49–53. 10.1016/0021-9150(86)90086-9 3085685

[B163] Jankovic-Karasoulos T. Bianco-Miotto T. Butler M. S. Butler L. M. McNeil C. M. O'Toole S. A. . (2020). Elevated levels of tumour apolipoprotein D independently predict poor outcome in breast cancer patients. Histopathology 76, 976–987. 10.1111/his.14081 31994214

[B164] Jansen P. J. Lütjohann D. Thelen K. M. von Bergmann K. van Leuven F. Ramaekers F. C. S. . (2009). Absence of ApoE upregulates murine brain ApoD and ABCA1 levels, but does not affect brain sterol levels, while human ApoE3 and human ApoE4 upregulate brain cholesterol precursor levels. J. Alzheimers Dis. 18, 319–329. 10.3233/JAD-2009-1150 19584433

[B165] Jensen L. D. Hot B. Ramsköld D. Germano R. F. V. Yokota C. Giatrellis S. . (2019). Disruption of the extracellular matrix progressively impairs central nervous system vascular maturation downstream of β-catenin signaling. Arterioscler. Thromb. Vasc. Biol. 39, 1432–1447. 10.1161/ATVBAHA.119.312388 31242033 PMC6597191

[B166] Jeong J. Bae H. Lim W. Bazer F. W. Song G. (2015). Diethylstilbestrol regulates expression of avian apolipoprotein D during regression and recrudescence of the oviduct and epithelial-derived ovarian carcinogenesis. Domest. Anim. Endocrinol. 52, 82–89. 10.1016/j.domaniend.2015.03.005 25929245

[B167] Jiang D. Jin H. Zuo J. Kong Y. Zhang X. Dong Q. . (2020). Potential biomarkers screening to predict side effects of dexamethasone in different cancers. Mol. Genet. Genomic Med. 8:e1160. 10.1002/mgg3.1160 32048780 PMC7196465

[B168] Jiang R. Rong C. Ke R. Meng S. Yan X. Ke H. . (2019). Differential proteomic analysis of serum exosomes reveals alterations in progression of Parkinson disease. Medicine 98:e17478. 10.1097/MD.0000000000017478 31593110 PMC6799836

[B169] Jiménez-Palomares M. Cózar-Castellano I. Ganfornina M. D. Sánchez D. Perdomo G. (2011). Genetic deficiency of apolipoprotein D in the mouse is associated with nonfasting hypertriglyceridemia and hyperinsulinemia. Metab. Clin. Exp. 60, 1767–1774. 10.1016/j.metabol.2011.04.013 21632073

[B170] Jin D. El-Tanani M. Campbell F. C. (2006). Identification of apolipoprotein D as a novel inhibitor of osteopontin-induced neoplastic transformation. Int. J. Oncol. 29, 1591–1599. 10.3892/ijo.29.6.1591 17089001

[B171] Jinawath N. Vasoontara C. Jinawath A. Fang X. Zhao K. Yap K.-L. . (2010). Oncoproteomic analysis reveals co-upregulation of RELA and STAT5 in carboplatin resistant ovarian carcinoma. PLoS ONE 5:e11198. 10.1371/journal.pone.0011198 20585448 PMC2887843

[B172] Kalman J. McConathy W. Araoz C. Kasa P. Lacko A. G. (2000). Apolipoprotein D in the aging brain and in Alzheimer's dementia. Neurol. Res. 22, 330–336. 10.1080/01616412.2000.11740678 10874678

[B173] Kamboh M. I. Albers J. J. Majumder P. P. Ferrell R. E. (1989). Genetic studies of human apolipoproteins. IX. Apolipoprotein D polymorphism and its relation to serum lipoprotein lipid levels. Am. J. Hum. Genet. 45, 147–154. 2741945 PMC1683387

[B174] Kang M. K. Kameta A. Shin K.-H. Baluda M. A. Kim H.-R. Park N.-H. (2003a). Senescence-associated genes in normal human oral keratinocytes. Exp. Cell Res. 287, 272–281. 10.1016/s0014-4827(03)00061-2 12837283

[B175] Kang S. Seo S. Hill J. Kwon B. Lee H. Cho H. . (2003b). Changes in gene expression in latent HSV-1-infected rabbit trigeminal ganglia following epinephrine iontophoresis. Curr. Eye Res. 26, 225–229. 10.1076/ceyr.26.3.225.14894 12815551

[B176] Kao L. C. Tulac S. Lobo S. Imani B. Yang J. P. Germeyer A. . (2002). Global gene profiling in human endometrium during the window of implantation. Endocrinology 143, 2119–2138. 10.1210/endo.143.6.8885 12021176

[B177] Kendal A. R. Layton T. Al-Mossawi H. Appleton L. Dakin S. Brown R. . (2020). Multi-omic single cell analysis resolves novel stromal cell populations in healthy and diseased human tendon. Sci. Rep. 10:13939. 10.1038/s41598-020-70786-5 32883960 PMC7471282

[B178] Kfir S. Basavaraja R. Wigoda N. Ben-Dor S. Orr I. Meidan R. (2018). Genomic profiling of bovine corpus luteum maturation. PLoS ONE 13:e0194456. 10.1371/journal.pone.0194456 29590145 PMC5874041

[B179] Khan M. M. Parikh V. V. Mahadik S. P. (2003). Antipsychotic drugs differentially modulate apolipoprotein D in rat brain. J. Neurochem. 86, 1089–1100. 10.1046/j.1471-4159.2003.01866.x 12911617

[B180] Khoder-Agha F. Kietzmann T. (2021). The glyco-redox interplay: principles and consequences on the role of reactive oxygen species during protein glycosylation. Redox Biol. 42:101888. 10.1016/j.redox.2021.101888 33602616 PMC8113034

[B181] Khoonsari P. E. Ossipova E. Lengqvist J. Svensson C. I. Kosek E. Kadetoff D. . (2019). The human CSF pain proteome. J. Proteomics 190, 67–76. 10.1016/j.jprot.2018.05.012 29852297

[B182] Kielkopf C. S. Ghosh M. Anand G. S. Brown S. H. J. (2019). HDX-MS reveals orthosteric and allosteric changes in apolipoprotein-D structural dynamics upon binding of progesterone. Protein Sci. 28, 365–374. 10.1002/pro.3534 30353968 PMC6319766

[B183] Kielkopf C. S. Low J. K. K. Mok Y.-F. Bhatia S. Palasovski T. Oakley A. J. . (2018). Identification of a novel tetrameric structure for human apolipoprotein-D. J. Struct. Biol. 203, 205–218. 10.1016/j.jsb.2018.05.012 29885491

[B184] Kielkopf C. S. Whitten A. E. Garner B. Brown S. H. J. (2021). Small angle X-ray scattering analysis of ligand-bound forms of tetrameric apolipoprotein-D. Biosci. Rep. 41:BSR20201423. 10.1042/BSR20201423 33399852 PMC7786332

[B185] Kim D. S. Lee S. J. Park S. Y. Yoo H. J. Kim S. H. Kim K. J. . (2001). Differentially expressed genes in rat dorsal root ganglia following peripheral nerve injury. Neuroreport 12, 3401–3405. 10.1097/00001756-200110290-00050 11711894

[B186] Kim W. S. Wong J. Weickert C. S. Webster M. J. Bahn S. Garner B. (2009). Apolipoprotein-D expression is increased during development and maturation of the human prefrontal cortex. J. Neurochem. 109, 1053–1066. 10.1111/j.1471-4159.2009.06031.x 19519777

[B187] Klebaner D. Hamilton-Dutoit S. Ahern T. Crawford A. Jakobsen T. Cronin-Fenton D. P. . (2017). Apolipoprotein D expression does not predict breast cancer recurrence among tamoxifen-treated patients. PLoS ONE 12:e0171453. 10.1371/journal.pone.0171453 28301514 PMC5354364

[B188] Kliuchnikova A. A. Samokhina N. I. Ilina I. Y. Karpov D. S. Pyatnitskiy M. A. Kuznetsova K. G. . (2016). Human aqueous humor proteome in cataract, glaucoma, and pseudoexfoliation syndrome. Proteomics 16, 1938–1946. 10.1002/pmic.201500423 27193151

[B189] Koch S. Donarski N. Goetze K. Kreckel M. Stuerenburg H. J. Buhmann C. . (2001). Characterization of four lipoprotein classes in human cerebrospinal fluid. J. Lipid Res. 42, 1143–1151. 10.1016/S0022-2275(20)31605-9 11441143

[B190] Kopylov A. T. Papysheva O. Gribova I. Kotaysch G. Kharitonova L. Mayatskaya T. . (2020). Molecular pathophysiology of diabetes mellitus during pregnancy with antenatal complications. Sci. Rep. 10:19641. 10.1038/s41598-020-76689-9 33184417 PMC7665025

[B191] Kosacka J. Gericke M. Nowicki M. Kacza J. Borlak J. Spanel-Borowski K. (2009). Apolipoproteins D and E3 exert neurotrophic and synaptogenic effects in dorsal root ganglion cell cultures. Neuroscience 162, 282–291. 10.1016/j.neuroscience.2009.04.073 19414061

[B192] Kosacka J. Schröder T. Bechmann I. Klöting N. Nowicki M. Mittag A. . (2011). PACAP up-regulates the expression of apolipoprotein D in 3T3-L1 adipocytes. DRG/3T3-L1 co-cultures study. Neurosci. Res. 69, 8–16. 10.1016/j.neures.2010.09.009 20920539

[B193] Kosarussavadi S. Pennington Z. T. Covell J. Blaisdell A. P. Schlinger B. A. (2017). Across sex and age: learning and memory and patterns of avian hippocampal gene expression. Behav. Neurosci. 131, 483–491. 10.1037/bne0000222 29189019 PMC5721522

[B194] Kristensen V. N. Sørlie T. Geisler J. Yoshimura N. Linegjaerde O.-C. Glad I. . (2005). Effects of anastrozole on the intratumoral gene expression in locally advanced breast cancer. J. Steroid Biochem. Mol. Biol. 95, 105–111. 10.1016/j.jsbmb.2005.04.028 16023338

[B195] Kroksveen A. C. Aasebø E. Vethe H. Van Pesch V. Franciotta D. Teunissen C. E. . (2013). Discovery and initial verification of differentially abundant proteins between multiple sclerosis patients and controls using iTRAQ and SID-SRM. J. Proteomics 78, 312–325. 10.1016/j.jprot.2012.09.037 23059536

[B196] Kroksveen A. C. Guldbrandsen A. Vedeler C. Myhr K. M. Opsahl J. A. Berven F. S. (2012). Cerebrospinal fluid proteome comparison between multiple sclerosis patients and controls. Acta Neurol. Scand., Suppl. 126, 90–96. 10.1111/ane.12029 23278663

[B197] Kuiperij H. B. Hondius D. C. Kersten I. Versleijen A. A. M. Rozemuller A. J. M. Greenberg S. M. . (2019). Apolipoprotein D: a potential biomarker for cerebral amyloid angiopathy. Neuropathol. Appl. Neurobiol. 46, 431–440. 10.1111/nan.12595 31872472

[B198] Kumar U. (2012). Immunohistochemical distribution of somatostatin and somatostatin receptor subtypes (SSTR1–5) in hypothalamus of ApoD knockout mice brain. J. Mol. Neurosci. 48, 684–695. 10.1007/s12031-012-9792-7 22581439

[B199] Labrie M. Lalonde S. Najyb O. Thiery M. Daneault C. Des Rosiers C. . (2015). Apolipoprotein D transgenic mice develop hepatic steatosis through activation of PPARγ and fatty acid uptake. PLoS ONE 10:e0130230. 10.1371/journal.pone.0130230 26083030 PMC4470830

[B200] Lai C.-J. Cheng H.-C. Lin C.-Y. Huang S.-H. Chen T.-H. Chung C.-J. . (2017). Activation of liver X receptor suppresses angiogenesis via induction of ApoD. FASEB J. 31, 5568–5576. 10.1096/fj.201700374R 28842423

[B201] Lambert C. Cubedo J. Padró T. Sánchez-Hernández J. Antonijoan R. M. Perez A. . (2017). Phytosterols and omega 3 supplementation exert novel regulatory effects on metabolic and inflammatory pathways: a proteomic study. Nutrients 9:599. 10.3390/nu9060599 28608804 PMC5490578

[B202] Lambert J. Provost P. R. Marcel Y. L. Rassart E. (1993). Structure of the human apolipoprotein D gene promoter region. Biochim. Biophys. Acta 1172, 190–192. 10.1016/0167-4781(93)90292-L 7916629

[B203] Lamelas M. L. Vázquez J. Enguita M. I. Rodríguez J. C. González L. O. Merino A. M. . (2000). Apolipoprotein D expression in metastasic lymph nodes of breast cancer. Int. J. Surg. Investig. 2, 285–293. 12678530

[B204] Lane D. M. Boatman K. K. McConathy W. J. (1995). Serum lipids and apolipoproteins in women with breast masses. Breast Cancer Res. Treat. 34, 161–169. 10.1007/BF00665788 7647333

[B205] Lea O. A. (1988). Binding properties of progesterone-binding Cyst protein, PBCP. Steroids 52, 337–338. 10.1016/0039-128X(88)90135-3 3250014

[B206] Lee M. Y. Kim E. Y. Kim S. H. Cho K.-C. Ha K. Kim K. P. . (2016). Discovery of serum protein biomarkers in drug-free patients with major depressive disorder. Prog. Neuropsychopharmacol. Biol. Psychiatry 69, 60–68. 10.1016/j.pnpbp.2016.04.009 27105922

[B207] Leung W. C. Y. Lawrie A. Demaries S. Massaeli H. Burry A. Yablonsky S. . (2004). Apolipoprotein D and platelet-derived growth factor-BB synergism mediates vascular smooth muscle cell migration. Circ. Res. 95, 179–186. 10.1161/01.RES.0000135482.74178.14 15192024

[B208] Levros L.-C. Do Carmo S. Edouard E. Legault P. Charfi C. Rassart E. (2010). Characterization of nuclear factors modulating the apolipoprotein D promoter during growth arrest: implication of PARP-1, APEX-1 and ERK1/2 catalytic activities. Biochim. Biophys. Acta 1803, 1062–1071. 10.1016/j.bbamcr.2010.04.011 20493910 PMC7114184

[B209] Levros L.-C. Labrie M. Charfi C. Rassart E. (2013). Binding and repressive activities of apolipoprotein E3 and E4 isoforms on the human ApoD promoter. Mol. Neurobiol. 48, 669–680. 10.1007/s12035-013-8456-0 23715769 PMC7090986

[B210] Li H. Repa J. J. Valasek M. A. Beltroy E. P. Turley S. D. German D. C. . (2005). Molecular, anatomical, and biochemical events associated with neurodegeneration in mice with Niemann-Pick type C disease. J. Neuropathol. Exp. Neurol. 64, 323–333. 10.1093/jnen/64.4.323 15835268

[B211] Li H. Ruberu K. Karl T. Garner B. (2016). Cerebral apolipoprotein-D is hypoglycosylated compared to peripheral tissues and is variably expressed in mouse and human brain regions. PLoS ONE 11:e0148238. 10.1371/journal.pone.0148238 26829325 PMC4734669

[B212] Li H. Ruberu K. Muñoz S. S. Jenner A. M. Spiro A. Zhao H. . (2015). Apolipoprotein D modulates amyloid pathology in APP/PS1 Alzheimer's disease mice. Neurobiol. Aging 36, 1820–1833. 10.1016/j.neurobiolaging.2015.02.010 25784209

[B213] Li J. Liu C. Chen Y. Gao C. Wang M. Ma X. . (2019). Tumor characterization in breast cancer identifies immune-relevant gene signatures associated with prognosis. Front. Genet. 10:1119. 10.3389/fgene.2019.01119 31781173 PMC6861325

[B214] Li X. Miyajima M. Mineki R. Taka H. Murayama K. Arai H. (2006). Analysis of potential diagnostic biomarkers in cerebrospinal fluid of idiopathic normal pressure hydrocephalus by proteomics. Acta Neurochir. 148, 859–864; discussion: 864. 10.1007/s00701-006-0787-4 16755327

[B215] Lieuallen K. Pennacchio L. A. Park M. Myers R. M. Lennon G. G. (2001). Cystatin B-deficient mice have increased expression of apoptosis and glial activation genes. Hum. Mol. Genet. 10, 1867–1871. 10.1093/hmg/10.18.1867 11555622

[B216] Lim W. Bae H. Song G. (2016). Differential expression of apolipoprotein D in male reproductive system of rats by high-fat diet. Andrology 4, 1115–1122. 10.1111/andr.12250 27566528

[B217] Lin C. S. Ho H. C. Gholami S. Chen K. C. Jad A. Lue T. F. (2001). Gene expression profiling of an arteriogenic impotence model. Biochem. Biophys. Res. Commun. 285, 565–569. 10.1006/bbrc.2001.5191 11444882

[B218] Lin Y.-S. Chang T.-H. Shi C.-S. Wang Y.-Z. Ho W.-C. Huang H.-D. . (2019). Liver X receptor/retinoid X receptor pathway plays a regulatory role in pacing-induced cardiomyopathy. J. Am. Heart Assoc. 8:e009146. 10.1161/JAHA.118.009146 30612502 PMC6405706

[B219] Linn S. C. West R. B. Pollack J. R. Zhu S. Hernandez-Boussard T. Nielsen T. O. . (2003). Gene expression patterns and gene copy number changes in dermatofibrosarcoma protuberans. Am. J. Pathol. 163, 2383–2395. 10.1016/S0002-9440(10)63593-6 14633610 PMC1892373

[B220] Lisovsky M. Hoang M. P. Dresser K. A. Kapur P. Bhawan J. Mahalingam M. (2008). Apolipoprotein D in CD34-positive and CD34-negative cutaneous neoplasms: a useful marker in differentiating superficial acral fibromyxoma from dermatofibrosarcoma protuberans. Mod. Pathol. 21, 31–38. 10.1038/modpathol.3800971 17885669

[B221] Liu H. Anders F. Funke S. Mercieca K. Grus F. Prokosch V. (2020). Proteome alterations in aqueous humour of primary open angle glaucoma patients. Int. J. Ophthalmol. 13, 176–179. 10.18240/ijo.2020.01.24 31956586 PMC6942938

[B222] Liu Y. Qiu N. Ma M. (2013). Comparative proteomic analysis of hen egg white proteins during early phase of embryonic development by combinatorial peptide ligand library and matrix-assisted laser desorption ionization-time of flight. Poult. Sci. 92, 1897–1904. 10.3382/ps.2012-02986 23776278

[B223] Liu Z. Chang G. Q. Leibowitz S. F. (2001). Apolipoprotein D interacts with the long-form leptin receptor: a hypothalamic function in the control of energy homeostasis. FASEB J. 15, 1329–1331. 10.1096/fj.00-0530fje 11344130

[B224] Loerch P. M. Lu T. Dakin K. A. Vann J. M. Isaacs A. Geula C. . (2008). Evolution of the aging brain transcriptome and synaptic regulation. PLoS ONE 3:e3329. 10.1371/journal.pone.0003329 18830410 PMC2553198

[B225] López-Boado Y. S. Klaus M. Dawson M. I. López-Otín C. (1996). Retinoic acid-induced expression of apolipoprotein D and concomitant growth arrest in human breast cancer cells are mediated through a retinoic acid receptor RARalpha-dependent signaling pathway. J. Biol. Chem. 271, 32105–32111. 10.1074/jbc.271.50.32105 8943263

[B226] López-Boado Y. S. Puente X. S. Alvarez S. Tolivia J. Binderup L. López-Otín C. (1997). Growth inhibition of human breast cancer cells by 1,25-dihydroxyvitamin D3 is accompanied by induction of apolipoprotein D expression. Cancer Res. 57, 4091–4097. 9307298

[B227] López-Boado Y. S. Tolivia J. López-Otín C. (1994). Apolipoprotein D gene induction by retinoic acid is concomitant with growth arrest and cell differentiation in human breast cancer cells. J. Biol. Chem. 269, 26871–26878. 10.1016/S0021-9258(18)47100-1 7929425

[B228] Lopez-Nunez O. Surrey L. F. Alaggio R. Herradura A. McGough R. L. John I. (2021). Novel APOD-GLI1 rearrangement in a sarcoma of unknown lineage. Histopathology 78, 338–340. 10.1111/his.14235 32798311

[B229] Lövkvist H. Jönsson A.-C. Luthman H. Jood K. Jern C. Wieloch T. . (2014). Variations in apolipoprotein D and sigma non-opioid intracellular receptor 1 genes with relation to risk, severity and outcome of ischemic stroke. BMC Neurol. 14:191. 10.1186/s12883-014-0191-2 25261976 PMC4186220

[B230] Lynch C. C. Hikosaka A. Acuff H. B. Martin M. D. Kawai N. Singh R. K. . (2005). MMP-7 promotes prostate cancer-induced osteolysis via the solubilization of RANKL. Cancer Cell 7, 485–496. 10.1016/j.ccr.2005.04.013 15894268

[B231] Mahadevan V. Soloff L. A. (1983). A method for isolating human plasma lecithin:cholesterol acyltransferase without using anti-apolipoprotein D, and its characterization. Biochim. Biophys. Acta 752, 89–97. 10.1016/0005-2760(83)90236-9 6405796

[B232] Mahadik S. P. Khan M. M. Evans D. R. Parikh V. V. (2002). Elevated plasma level of apolipoprotein D in schizophrenia and its treatment and outcome. Schizophr. Res. 58, 55–62. 10.1016/S0920-9964(01)00378-4 12363390

[B233] Manjunatha S. Distelmaier K. Dasari S. Carter R. E. Kudva Y. C. Nair K. S. (2016). Functional and proteomic alterations of plasma high density lipoproteins in type 1 diabetes mellitus. Metab. Clin. Exp. 65, 1421–1431. 10.1016/j.metabol.2016.06.008 27506748

[B234] Manousaki T. Hull P. M. Kusche H. Machado-Schiaffino G. Franchini P. Harrod C. . (2013). Parsing parallel evolution: ecological divergence and differential gene expression in the adaptive radiations of thick-lipped Midas cichlid fishes from Nicaragua. Mol. Ecol. 22, 650–669. 10.1111/mec.12034 23057963

[B235] Martineau C. Najyb O. Signor C. Rassart É. Moreau R. (2016). Apolipoprotein D deficiency is associated to high bone turnover, low bone mass and impaired osteoblastic function in aged female mice. Metab. Clin. Exp. 65, 1247–1258. 10.1016/j.metabol.2016.05.007 27506732 PMC7094319

[B236] Martínez E. Navarro A. Ordóñez C. Del Valle E. Tolivia J. (2012). Amyloid-β25-35 induces apolipoprotein D Synthesis and growth arrest in HT22 hippocampal cells. J. Alzheimers Dis. 30, 233–244. 10.3233/JAD-2012-112102 22398376

[B237] Martínez E. Navarro A. Ordóñez C. Del Valle E. Tolivia J. (2013). Oxidative stress induces apolipoprotein D overexpression in hippocampus during aging and Alzheimer's disease. J. Alzheimers Dis. 36, 129–144. 10.3233/JAD-130215 23568103

[B238] Martínez-Pinilla E. Navarro A. Ordóñez C. del Valle E. Tolivia J. (2015). Apolipoprotein D subcellular distribution pattern in neuronal cells during oxidative stress. Acta Histochem. 117, 536–544. 10.1016/j.acthis.2015.04.003 25953740

[B239] Martínez-Pinilla E. Rubio-Sardón N. Peláez R. García-Álvarez E. del Valle E. Tolivia J. . (2021). Neuroprotective effect of apolipoprotein D in cuprizone-induced cell line models: a potential therapeutic approach for multiple sclerosis and demyelinating diseases. Int. J. Mol. Sci. 22:1260. 10.3390/ijms22031260 33514021 PMC7866080

[B240] Mattsson N. Insel P. Nosheny R. Trojanowski J. Q. Shaw L. M. Jack C. R. . (2014). Effects of cerebrospinal fluid proteins on brain atrophy rates in cognitively healthy older adults. Neurobiol. Aging 35, 614–622. 10.1016/j.neurobiolaging.2013.08.027 24094581 PMC3864623

[B241] Mazoujian G. Haagensen D. E. (1990). The immunopathology of gross cystic disease fluid proteins. Ann. N. Y. Acad. Sci. 586, 188–197. 10.1111/j.1749-6632.1990.tb17806.x 2357000

[B242] McConathy W. J. Alaupovic P. (1973). Isolation and partial characterization of apolipoprotein D: a new protein moiety of the human plasma lipoprotein system. FEBS Lett. 37, 178–182. 10.1016/0014-5793(73)80453-3 4128506

[B243] McConathy W. J. Alaupovic P. (1976). Studies on the isolation and partial characterization of apolipoprotein D and lipoprotein D of human plasma. Biochemistry 15, 515–520. 10.1021/bi00648a010 56198

[B244] McConathy W. J. Alaupovic P. (1986). Isolation and characterization of other apolipoproteins. Meth. Enzymol. 128, 297–310. 10.1016/0076-6879(86)28075-1 3724508

[B245] Mejias A. Diez-Hermano S. Ganfornina M. D. Gutierrez G. Sanchez D. (2019). Characterization of mammalian Lipocalin UTRs *in silico*: predictions for their role in post-transcriptional regulation. PLoS ONE 14:e0213206. 10.1371/journal.pone.0213206 30840684 PMC6402760

[B246] Miranda E. Vizoso F. Martín A. Quintela I. Corte M. D. Seguí M. E. . (2003). Apolipoprotein D expression in cutaneous malignant melanoma. J. Surg. Oncol. 83, 99–105. 10.1002/jso.10245 12772203

[B247] Mirza Z. Abdel-Dayem U. A. (2020). Uncovering potential roles of differentially expressed genes, upstream regulators, and canonical pathways in endometriosis using an *in silico* genomics approach. Diagnostics 10:416. 10.3390/diagnostics10060416 32575462 PMC7344784

[B248] Mohammed A. Janakiram N. B. Suen C. Stratton N. Lightfoot S. Singh A. . (2019). Targeting cholecystokinin-2 receptor for pancreatic cancer chemoprevention. Mol. Carcinog. 58, 1908–1918. 10.1002/mc.23084 31313401 PMC6721979

[B249] Molnár A. Gyurján I. Korpos E. Borsy A. Stéger V. Buzás Z. . (2007). Identification of differentially expressed genes in the developing antler of red deer *Cervus elaphus*. Mol. Genet. Genomics 277, 237–248. 10.1007/s00438-006-0193-x 17131158

[B250] Montpied P. de Bock F. Lerner-Natoli M. Bockaert J. Rondouin G. (1999). Hippocampal alterations of apolipoprotein E and D mRNA levels *in vivo* and *in vitro* following kainate excitotoxicity. Epilepsy Res. 35, 135–146. 10.1016/S0920-1211(99)00003-0 10372566

[B251] Morais Cabral J. H. Atkins G. L. Sánchez L. M. López-Boado Y. S. López-Otin C. Sawyer L. (1995). Arachidonic acid binds to apolipoprotein D: implications for the protein's function. FEBS Lett. 366, 53–56. 10.1016/0014-5793(95)00484-Q 7789516

[B252] Morton R. E. Zilversmit D. B. (1981). The separation of apolipoprotein D from cholesteryl ester transfer protein. Biochim. Biophys. Acta 663, 350–355. 10.1016/0005-2760(81)90220-4 6783110

[B253] Muffat J. Walker D. W. Benzer S. (2008). Human ApoD, an apolipoprotein up-regulated in neurodegenerative diseases, extends lifespan and increases stress resistance in Drosophila. Proc. Natl. Acad. Sci. U.S.A. 105, 7088–7093. 10.1073/pnas.0800896105 18458334 PMC2374552

[B254] Najyb O. Brissette L. Rassart E. (2015). Apolipoprotein D internalization is a basigin-dependent mechanism. J. Biol. Chem. 290, 16077–16087. 10.1074/jbc.M115.644302 25918162 PMC4481210

[B255] Najyb O. Do Carmo S. Alikashani A. Rassart E. (2017). Apolipoprotein D overexpression protects against kainate-induced neurotoxicity in mice. Mol. Neurobiol. 54, 3948–3963. 10.1007/s12035-016-9920-4 27271124 PMC7091089

[B256] Namdar-Aligoodarzi P. Mohammadparast S. Zaker-Kandjani B. Talebi Kakroodi S. Jafari Vesiehsari M. Ohadi M. (2015). Exceptionally long 5' UTR short tandem repeats specifically linked to primates. Gene 569, 88–94. 10.1016/j.gene.2015.05.053 26022613

[B257] Nasreen A. Vogt M. Kim H. J. Eichinger A. Skerra A. (2006). Solubility engineering and crystallization of human apolipoprotein D. Protein Sci. 15, 190–199. 10.1110/ps.051775606 16322568 PMC2242363

[B258] Navarro A. Alonso A. Garrido P. González C. González Del Rey C. Ordoñez C. . (2010a). Increase in placental apolipoprotein D as an adaptation to human gestational diabetes. Placenta 31, 25–31. 10.1016/j.placenta.2009.11.002 19944460 PMC7124627

[B259] Navarro A. Del Valle E. Astudillo A. González del Rey C. Tolivia J. (2003). Immunohistochemical study of distribution of apolipoproteins E and D in human cerebral beta amyloid deposits. Exp. Neurol. 184, 697–704. 10.1016/S0014-4886(03)00315-7 14769361

[B260] Navarro A. del Valle E. Juárez A. Martinez E. Ordóñez C. Astudillo A. . (2010b). Apolipoprotein D synthesis progressively increases in frontal cortex during human lifespan. Age 32, 85–96. 10.1007/s11357-009-9117-0 19936966 PMC2829646

[B261] Navarro A. Del Valle E. Tolivia J. (2004). Differential expression of apolipoprotein d in human astroglial and oligodendroglial cells. J. Histochem. Cytochem. 52, 1031–1036. 10.1369/jhc.3A6213.2004 15258178

[B262] Navarro A. Méndez E. Diaz C. del Valle E. Martínez-Pinilla E. Ordóñez C. . (2013). Lifelong expression of apolipoprotein D in the human brainstem: correlation with reduced age-related neurodegeneration. PLoS ONE 8:e77852. 10.1371/journal.pone.0077852 24167586 PMC3805570

[B263] Navarro A. Ordóñez C. Martínez E. Pérez C. Astudillo A. Tolivia J. (2008). Apolipoprotein D expression absence in degenerating neurons of human central nervous system. Histol. Histopathol. 23, 995–1001. 10.14670/HH-23.995 18498075

[B264] Navarro A. Rioseras B. Del Valle E. Martínez-Pinilla E. Astudillo A. Tolivia J. (2018). Expression Pattern of Myelin-Related Apolipoprotein D in Human Multiple Sclerosis Lesions. Front. Aging Neurosci. 10:254. 10.3389/fnagi.2018.00254 30186153 PMC6110904

[B265] Navarro A. Tolivia J. Astudillo A. del Valle E. (1998). Pattern of apolipoprotein D immunoreactivity in human brain. Neurosci. Lett. 254, 17–20. 10.1016/S0304-3940(98)00639-9 9780081

[B266] Nazarenko M. S. Markov A. V. Sleptsov A. A. Koroleva I. A. Sharysh D. V. Zarubin A. A. . (2018). Comparative analysis of gene expression in vascular cells of patients with advanced atherosclerosis. Biomed. Khim. 64, 416–422. 10.18097/PBMC20186405416 30378557

[B267] Ng M. W. Angerosa J. Konstantinov I. E. Cheung M. M. Pepe S. (2019). Remote ischaemic preconditioning modifies serum apolipoprotein D, met-enkephalin, adenosine, and nitric oxide in healthy young adults. Clin. Exp. Pharmacol. Physiol. 46, 995–1000. 10.1111/1440-1681.13150 31361911

[B268] Nowicki M. Kosacka J. Spanel-Borowski K. Borlak J. (2009). Deferoxamine-induced neurite outgrowth and synapse formation in postnatal rat dorsal root ganglion (DRG) cell cultures. Eur. J. Cell Biol. 88, 551–562. 10.1016/j.ejcb.2009.05.003 19581022

[B269] Oakley A. J. Bhatia S. Ecroyd H. Garner B. (2012). Molecular dynamics analysis of apolipoprotein-D-lipid hydroperoxide interactions: mechanism for selective oxidation of Met-93. PLoS ONE 7:e34057. 10.1371/journal.pone.0034057 22479522 PMC3316614

[B270] O'Donnell J. Stemmelin J. Nitta A. Brouillette J. Quirion R. (2003). Gene expression profiling following chronic NMDA receptor blockade-induced learning deficits in rats. Synapse 50, 171–180. 10.1002/syn.10258 14515334

[B271] Ogawa K. Utsunomiya T. Mimori K. Yamashita K. Okamoto M. Tanaka F. . (2005). Genomic screens for genes upregulated by demethylation in colorectal cancer: possible usefulness for clinical application. Int. J. Oncol. 27, 417–426. 10.3892/ijo.27.2.417 16010423

[B272] Oláh Z. Kálmán J. Tóth M. E. Zvara Á. Sántha M. Ivitz E. . (2015). Proteomic analysis of cerebrospinal fluid in Alzheimer's disease: wanted dead or alive. J. Alzheimers Dis. 44, 1303–1312. 10.3233/JAD-140141 25428253

[B273] Ong W.-Y. Hu C.-Y. Patel S. C. (2002). Apolipoprotein D in the Niemann-Pick type C disease mouse brain: an ultrastructural immunocytochemical analysis. J. Neurocytol. 31, 121–129. 10.1023/A:1023993405851 12815234

[B274] Ong W. Y. He Y. Suresh S. Patel S. C. (1997). Differential expression of apolipoprotein D and apolipoprotein E in the kainic acid-lesioned rat hippocampus. Neuroscience 79, 359–367. 10.1016/S0306-4522(96)00608-2 9200721

[B275] Ong W. Y. Lau C. P. Leong S. K. Kumar U. Suresh S. Patel S. C. (1999). Apolipoprotein D gene expression in the rat brain and light and electron microscopic immunocytochemistry of apolipoprotein D expression in the cerebellum of neonatal, immature and adult rats. Neuroscience 90, 913–922. 10.1016/S0306-4522(98)00507-7 10218791

[B276] Ordoñez C. Navarro A. Perez C. Astudillo A. Martínez E. Tolivia J. (2006). Apolipoprotein D expression in substantia nigra of Parkinson disease. Histol. Histopathol. 21, 361–366. 10.14670/HH-21.361 16437381

[B277] Ordóñez C. Navarro A. Pérez C. Martínez E. del Valle E. Tolivia J. (2012). Gender differences in apolipoprotein D expression during aging and in Alzheimer disease. Neurobiol. Aging 33, 433.e11–e20. 10.1016/j.neurobiolaging.2011.01.010 21429623

[B278] Osundeko O. Tetlow L. Bundred N. Gowland E. (1997). Radioimmunoassay for serum apolipoprotein D, an atypical apolipoprotein: validation and clinical application. Ann. Clin. Biochem. 34(Pt. 5), 537–542. 10.1177/000456329703400508 9293309

[B279] Pajaniappan M. Glober N. K. Kennard S. Liu H. Zhao N. Lilly B. (2011). Endothelial cells downregulate apolipoprotein D expression in mural cells through paracrine secretion and Notch signaling. Am. J. Physiol. Heart Circ. Physiol. 301, H784–H793. 10.1152/ajpheart.00116.2011 21705670 PMC3191092

[B280] Palmerini E. Gambarotti M. Staals E. L. Zanella L. Sieberova G. Longhi A. . (2012). Fibrosarcomatous changes and expression of CD34+ and apolipoprotein-D in dermatofibrosarcoma protuberans. Clin. Sarcoma Res. 2:4. 10.1186/2045-3329-2-4 22587823 PMC3351741

[B281] Pamir N. Hutchins P. Ronsein G. Vaisar T. Reardon C. A. Getz G. S. . (2016). Proteomic analysis of HDL from inbred mouse strains implicates APOE associated with HDL in reduced cholesterol efflux capacity via the ABCA1 pathway. J. Lipid Res. 57, 246–257. 10.1194/jlr.M063701 26673204 PMC4727420

[B282] Pascua-Maestro R. Corraliza-Gomez M. Fadrique-Rojo C. Ledesma M. D. Schuchman E. H. Sanchez D. . (2020). Apolipoprotein D-mediated preservation of lysosomal function promotes cell survival and delays motor impairment in Niemann-Pick type A disease. Neurobiol. Dis. 144:105046. 10.1016/j.nbd.2020.105046 32798728

[B283] Pascua-Maestro R. Diez-Hermano S. Lillo C. Ganfornina M. D. Sanchez D. (2017). Protecting cells by protecting their vulnerable lysosomes: identification of a new mechanism for preserving lysosomal functional integrity upon oxidative stress. PLoS Genet. 13:e1006603. 10.1371/journal.pgen.1006603 28182653 PMC5325589

[B284] Pascua-Maestro R. González E. Lillo C. Ganfornina M. D. Falcón-Pérez J. M. Sanchez D. (2018). Extracellular vesicles secreted by astroglial cells transport apolipoprotein D to neurons and mediate neuronal survival upon oxidative stress. Front. Cell Neurosci. 12:526. 10.3389/fncel.2018.00526 30687015 PMC6335244

[B285] Patel R. C. Lange D. McConathy W. J. Patel Y. C. Patel S. C. (1997). Probing the structure of the ligand binding cavity of lipocalins by fluorescence spectroscopy. Protein Eng. 10, 621–625. 10.1093/protein/10.6.621 9278274

[B286] Patel S. C. Asotra K. Patel Y. C. McConathy W. J. Patel R. C. Suresh S. (1995). Astrocytes synthesize and secrete the lipophilic ligand carrier apolipoprotein D. Neuroreport 6, 653–657. 10.1097/00001756-199503000-00017 7605920

[B287] Peitsch M. C. Boguski M. S. (1990). Is apolipoprotein D a mammalian bilin-binding protein? New Biol. 2, 197–206. 2083249

[B288] Perdomo G. Henry Dong H. (2009). Apolipoprotein D in lipid metabolism and its functional implication in atherosclerosis and aging. Aging 1, 17–27. 10.18632/aging.100004 19946382 PMC2784685

[B289] Perdomo G. Kim D. H. Zhang T. Qu S. Thomas E. A. Toledo F. G. S. . (2010). A role of apolipoprotein D in triglyceride metabolism. J. Lipid Res. 51, 1298–1311. 10.1194/jlr.M001206 20124557 PMC3035493

[B290] Pérez C. Navarro A. Martínez E. Ordóñez C. Del Valle E. Tolivia J. (2012). Age-related changes of apolipoprotein D expression in female rat central nervous system with chronic estradiol treatment. Age 34, 895–904. 10.1007/s11357-011-9286-5 21761133 PMC3682073

[B291] Perrotte M. Le Page A. Fournet M. Le Sayec M. Rassart É. Fulop T. . (2019). Blood-based redox-signature and their association to the cognitive scores in MCI and Alzheimer's disease patients. Free Radic. Biol. Med. 130, 499–511. 10.1016/j.freeradbiomed.2018.10.452 30445127

[B292] Phillips M. C. (2018). Is ABCA1 a lipid transfer protein? J. Lipid Res. 59, 749–763. 10.1194/jlr.R082313 29305383 PMC5928442

[B293] Piórkowska K. Zukowski K. Ropka-Molik K. Tyra M. Gurgul A. (2018). A comprehensive transcriptome analysis of skeletal muscles in two Polish pig breeds differing in fat and meat quality traits. Genet. Mol. Biol. 41, 125–136. 10.1590/1678-4685-gmb-2016-0101 29658965 PMC5901489

[B294] Pleva L. Kusnierova P. Plevova P. Zapletalova J. Karpisek M. Faldynova L. . (2015). Increased levels of MMP-3, MMP-9 and MPO represent predictors of in-stent restenosis, while increased levels of ADMA, LCAT, ApoE and ApoD predict bare metal stent patency. Biomed. Pap. Med. Fac. Univ. Palacky Olomouc Czech Repub. 159, 586–594. 10.5507/bp.2015.037 26365933

[B295] Ponnikorn S. Mongkolrob R. Klongthalay S. Roytrakul S. Srisanga K. Tungpradabkul S. . (2019). Comparative proteome-wide analysis of bone marrow microenvironment of β-thalassemia/hemoglobin E. Proteomes 7:8. 10.3390/proteomes7010008 30813444 PMC6473223

[B296] Provost P. R. Marcel Y. L. Milne R. W. Weech P. K. Rassart E. (1991a). Apolipoprotein D transcription occurs specifically in nonproliferating quiescent and senescent fibroblast cultures. FEBS Lett. 290, 139–141. 1915865 10.1016/0014-5793(91)81244-3

[B297] Provost P. R. Tremblay Y. el-Amine M. Bélanger A. (1995). Guinea pig apolipoprotein D RNA diversity, and developmental and gestational modulation of mRNA levels. Mol. Cell. Endocrinol. 109, 225–236. 10.1016/0303-7207(95)03506-3 7664986

[B298] Provost P. R. Villeneuve L. Weech P. K. Milne R. W. Marcel Y. L. Rassart E. (1991b). Localization of the major sites of rabbit apolipoprotein D gene transcription by *in situ* hybridization. J. Lipid Res. 32, 1959–1970. 1816324

[B299] Provost P. R. Weech P. K. Tremblay N. M. Marcel Y. L. Rassart E. (1990). Molecular characterization and differential mRNA tissue distribution of rabbit apolipoprotein D. J. Lipid Res. 31, 2057–2065. 10.1016/S0022-2275(20)42270-9 2086704

[B300] Przybycien-Szymanska M. M. Yang Y. Ashley W. W. (2016). Microparticle derived proteins as potential biomarkers for cerebral vasospasm post subarachnoid hemorrhage. A preliminary study. Clin. Neurol Neurosurg. 141, 48–55. 10.1016/j.clineuro.2015.12.012 26736019

[B301] Puntambekar S. S. Davis D. S. Hawel L. Crane J. Byus C. V. Carson M. J. (2011). LPS-induced CCL2 expression and macrophage influx into the murine central nervous system is polyamine-dependent. Brain Behav. Immun. 25, 629–639. 10.1016/j.bbi.2010.12.016 21237263 PMC3081407

[B302] Qin W. Pan J. Bauman W. A. Cardozo C. P. (2010). Differential alterations in gene expression profiles contribute to time-dependent effects of nandrolone to prevent denervation atrophy. BMC Genomics 11:596. 10.1186/1471-2164-11-596 20969782 PMC3091741

[B303] Qin Y. Chen Y. Yang J. Wu F. Zhao L. Yang F. . (2017). Serum glycopattern and Maackia amurensis lectin-II binding glycoproteins in autism spectrum disorder. Sci. Rep. 7:46041. 10.1038/srep46041 28485374 PMC5423032

[B304] Quaresima B. Crugliano T. Gaspari M. Faniello M. C. Cosimo P. Valanzano R. . (2008). A proteomics approach to identify changes in protein profiles in serum of Familial Adenomatous Polyposis patients. Cancer Lett. 272, 40–52. 10.1016/j.canlet.2008.06.021 18667268

[B305] Rajput P. S. Billova S. Patel S. C. Kharmate G. Somvanshi R. K. Kumar U. (2009). Expression of somatostatin and somatostatin receptor subtypes in Apolipoprotein D (ApoD) knockout mouse brain: an immunohistochemical analysis. J. Chem. Neuroanat. 38, 20–33. 10.1016/j.jchemneu.2009.05.004 19465111

[B306] Ravnsborg T. Andersen L. L. T. Trabjerg N. D. Rasmussen L. M. Jensen D. M. Overgaard M. (2016). First-trimester multimarker prediction of gestational diabetes mellitus using targeted mass spectrometry. Diabetologia 59, 970–979. 10.1007/s00125-016-3869-8 26818149

[B307] Reindl M. Knipping G. Wicher I. Dilitz E. Egg R. Deisenhammer F. . (2001). Increased intrathecal production of apolipoprotein D in multiple sclerosis. J. Neuroimmunol. 119, 327–332. 10.1016/S0165-5728(01)00378-2 11585636

[B308] Ricci F. Kern S. E. Hruban R. H. Iacobuzio-Donahue C. A. (2005). Stromal responses to carcinomas of the pancreas: juxtatumoral gene expression conforms to the infiltrating pattern and not the biologic subtype. Cancer Biol. Ther. 4, 302–307. 10.4161/cbt.4.3.1501 15876873

[B309] Rickhag M. Deierborg T. Patel S. Ruscher K. Wieloch T. (2008). Apolipoprotein D is elevated in oligodendrocytes in the peri-infarct region after experimental stroke: influence of enriched environment. J. Cereb. Blood Flow Metab. 28, 551–562. 10.1038/sj.jcbfm.9600552 17851453

[B310] Rickhag M. Wieloch T. Gid,ö G. Elmér E. Krogh M. Murray J. . (2006). Comprehensive regional and temporal gene expression profiling of the rat brain during the first 24 h after experimental stroke identifies dynamic ischemia-induced gene expression patterns, and reveals a biphasic activation of genes in surviving tissue. J. Neurochem. 96, 14–29. 10.1111/j.1471-4159.2005.03508.x 16300643

[B311] Rodríguez J. C. Díaz M. González L. O. Sánchez J. Sánchez M. T. Merino A. M. . (2000). Apolipoprotein D expression in benign and malignant prostate tissues. Int. J. Surg. Investig. 2, 319–326. 12678535

[B312] Rojo J. V. González L. O. Lamelas M. L. Merino A. Vizoso F. (2001). Apolipoprotein D expression in endometrial carcinomas. Acta Obstet. Gynecol. Scand. 80, 158–161. 10.1034/j.1600-0412.2001.080002158.x 11167212

[B313] Ruiz García M. (2013). Lazarillo and related lipocalins: ligands and functions (Ph.D. thesis). Universidad de Valladolid, Valladolid, Spain.

[B314] Ruiz M. Sanchez D. Correnti C. Strong R. K. Ganfornina M. D. (2013). Lipid-binding properties of human ApoD and Lazarillo-related lipocalins: functional implications for cell differentiation. FEBS J. 280, 3928–3943. 10.1111/febs.12394 23777559

[B315] Ruscher K. Erickson A. Kuric E. Inácio A. R. Wieloch T. (2010). Effects of chronic Clozapine administration on apolipoprotein D levels and on functional recovery following experimental stroke. Brain Res. 1321, 152–163. 10.1016/j.brainres.2010.01.024 20083089

[B316] Saadane A. Mast N. Trichonas G. Chakraborty D. Hammer S. Busik J. V. . (2019). Retinal vascular abnormalities and microglia activation in mice with deficiency in cytochrome P450 46A1-mediated cholesterol removal. Am. J. Pathol. 189, 405–425. 10.1016/j.ajpath.2018.10.013 30448403 PMC6360352

[B317] Saha S. Rangarajan P. N. (2003). Common host genes are activated in mouse brain by Japanese encephalitis and rabies viruses. J. Gen. Virol. 84, 1729–1735. 10.1099/vir.0.18826-0 12810866

[B318] Salami M. Bandegi A. R. Sameni H. R. Vafaei A. A. Pakdel A. (2019). hippocampal up-regulation of apolipoprotein D in a rat model of maternal hypo- and hyperthyroidism: implication of oxidative stress. Neurochem. Res. 44, 2190–2201. 10.1007/s11064-019-02859-5 31414343

[B319] Sałkowska A. Kara,ś K. Karwaciak I. Walczak-Drzewiecka A. Krawczyk M. Sobalska-Kwapis M. . (2020). Identification of novel molecular markers of human Th17 cells. Cells 9:1611. 10.3390/cells9071611 32635226 PMC7407666

[B320] Salvatore A. Cigliano L. Bucci E. M. Corpillo D. Velasco S. Carlucci A. . (2007). Haptoglobin binding to apolipoprotein A-I prevents damage from hydroxyl radicals on its stimulatory activity of the enzyme lecithin-cholesterol acyl-transferase. Biochemistry 46, 11158–11168. 10.1021/bi7006349 17824618

[B321] Sanchez D. Bajo-Grañeras R. Del Caño-Espinel M. Garcia-Centeno R. Garcia-Mateo N. Pascua-Maestro R. . (2015). Aging without apolipoprotein D: molecular and cellular modifications in the hippocampus and cortex. Exp. Gerontol. 67, 19–47. 10.1016/j.exger.2015.04.003 25868396

[B322] Sanchez D. Ganfornina M. D. Gutiérrez G. Gauthier-Jauneau A.-C. Risler J.-L. Salier J.-P. (2006). Lipocalin genes and their evolutionary history, in Molecular Biology Intelligence Unit: Lipocalins, 5–16. Available onnline at: https://uvadoc.uva.es/handle/10324/6219 (accessed June 29, 2021).

[B323] Sánchez D. Ganfornina M. D. Gutiérrez G. Marín A. (2003). Exon-intron structure and evolution of the lipocalin gene family. Mol. Biol. Evol. 20, 775–783. 10.1093/molbev/msg079 12679526

[B324] Sánchez D. Ganfornina M. D. Martínez S. (2002). Expression pattern of the lipocalin apolipoprotein D during mouse embryogenesis. Mech. Dev. 110, 225–229. 10.1016/S0925-4773(01)00578-0 11744388

[B325] Sánchez L. M. Díez-Itza I. Vizoso F. López-Otín C. (1992a). Cholesterol and apolipoprotein D in gross cystic disease of the breast. Clin. Chem. 38, 695–698. 1582022

[B326] Sánchez L. M. Vizoso F. Díez-Itza I. López-Otín C. (1992b). Identification of the major protein components in breast secretions from women with benign and malignant breast diseases. Cancer Res. 52, 95–100. 1727390

[B327] Sandim V. Pereira D. de A. Kalume D. E. Oliveira-Carvalho A. L. Ornellas A. A. . (2016). Proteomic analysis reveals differentially secreted proteins in the urine from patients with clear cell renal cell carcinoma. Urol. Oncol. 34, 5.e11–e25. 10.1016/j.urolonc.2015.07.016 26420021

[B328] Santana M. F. M. Lira A. L. A. Pinto R. S. Minanni C. A. Silva A. R. M. Sawada M. I. B. A. C. . (2020). Enrichment of apolipoprotein A-IV and apolipoprotein D in the HDL proteome is associated with HDL functions in diabetic kidney disease without dialysis. Lipids Health Dis. 19:205. 10.1186/s12944-020-01381-w 32921312 PMC7488728

[B329] Sarjeant J. M. Lawrie A. Kinnear C. Yablonsky S. Leung W. Massaeli H. . (2003). Apolipoprotein D inhibits platelet-derived growth factor-BB-induced vascular smooth muscle cell proliferated by preventing translocation of phosphorylated extracellular signal regulated kinase 1/2 to the nucleus. Arterioscler. Thromb. Vasc. Biol. 23, 2172–2177. 10.1161/01.ATV.0000100404.05459.39 14551159

[B330] Sasaki Y. Negishi H. Koyama R. Anbo N. Ohori K. Idogawa M. . (2009). p53 family members regulate the expression of the apolipoprotein D gene. J. Biol. Chem. 284, 872–883. 10.1074/jbc.M807185200 19001418

[B331] Satoh F. Umemura S. Osamura R. Y. (2000). Immunohistochemical analysis of GCDFP-15 and GCDFP-24 in mammary and non-mammary tissue. Breast Cancer 7, 49–55. 10.1007/BF02967188 11029771

[B332] Scalf C. S. Chariker J. H. Rouchka E. C. Ashley N. T. (2019). Transcriptomic analysis of immune response to bacterial lipopolysaccharide in zebra finch (Taeniopygia guttata). BMC Genomics 20:647. 10.1186/s12864-019-6016-3 31412766 PMC6693190

[B333] Schaeren-Wiemers N. Schaefer C. Valenzuela D. M. Yancopoulos G. D. Schwab M. E. (1995). Identification of new oligodendrocyte- and myelin-specific genes by a differential screening approach. J. Neurochem. 65, 10–22. 10.1046/j.1471-4159.1995.65010010.x 7790852

[B334] Schäfer N. F. Luhmann U. F. O. Feil S. Berger W. (2009). Differential gene expression in Ndph-knockout mice in retinal development. Invest. Ophthalmol. Vis. Sci. 50, 906–916. 10.1167/iovs.08-1731 18978344

[B335] Schindler P. A. Settineri C. A. Collet X. Fielding C. J. Burlingame A. L. (1995). Site-specific detection and structural characterization of the glycosylation of human plasma proteins lecithin:cholesterol acyltransferase and apolipoprotein D using HPLC/electrospray mass spectrometry and sequential glycosidase digestion. Protein Sci. 4, 791–803. 10.1002/pro.5560040419 7613477 PMC2143102

[B336] Schlotter F. de Freitas R. C. C. Rogers M. A. Blaser M. C. Wu P.-J. Higashi H. . (2020). ApoC-III is a novel inducer of calcification in human aortic valves. J. Biol. Chem. 296:100193. 10.1074/jbc.RA120.015700 33334888 PMC7948477

[B337] Schröder B. Elsässer H.-P. Schmidt B. Hasilik A. (2007). Characterisation of lipofuscin-like lysosomal inclusion bodies from human placenta. FEBS Lett. 581, 102–108. 10.1016/j.febslet.2006.12.005 17174955

[B338] Séguin D. Desforges M. Rassart E. (1995). Molecular characterization and differential mRNA tissue distribution of mouse apolipoprotein D. Brain Res. Mol. Brain Res. 30, 242–250. 10.1016/0169-328X(95)00008-G 7637575

[B339] Selim A. A. El-Ayat G. Wells C. A. (2001). Immunohistochemical localization of gross cystic disease fluid protein-15,−24 and−44 in ductal carcinoma *in situ* of the breast: relationship to the degree of differentiation. Histopathology 39, 198–202. 10.1046/j.1365-2559.2001.01178.x 11493337

[B340] Serra Díaz C. Vizoso F. Lamelas M. L. Rodríguez J. C. González L. O. Baltasar A. . (1999). Expression and clinical significance of apolipoprotein D in male breast cancer and gynaecomastia. Br. J. Surg. 86, 1190–1197. 10.1046/j.1365-2168.1999.01157.x 10504376

[B341] Serra C. Vizoso F. Lamelas M. L. Rodríguez J. C. González L. O. Merino A. M. . (2000). Comparative study of two androgen-induced markers (apolipoprotein D and pepsinogen C) in female and male breast carcinoma. Int. J. Surg. Investig. 2, 183–192. 12678518

[B342] Shibata N. Nagata T. Shinagawa S. Ohnuma T. Shimazaki H. Komatsu M. . (2013). Genetic association between APOA1 and APOD polymorphisms and Alzheimer's disease in a Japanese population. J. Neural Transm. 120, 1599–1603. 10.1007/s00702-013-1036-7 23690001

[B343] Simard J. Dauvois S. Haagensen D. E. Lévesque C. Mérand Y. Labrie F. (1990). Regulation of progesterone-binding breast cyst protein GCDFP-24 secretion by estrogens and androgens in human breast cancer cells: a new marker of steroid action in breast cancer. Endocrinology 126, 3223–3231. 10.1210/endo-126-6-3223 2351114

[B344] Simard J. de Launoit Y. Haagensen D. E. Labrie F. (1992). Additive stimulatory action of glucocorticoids and androgens on basal and estrogen-repressed apolipoprotein-D messenger ribonucleic acid levels and secretion in human breast cancer cells. Endocrinology 130, 1115–1121. 1537279 10.1210/endo.130.3.1537279

[B345] Simard J. Veilleux R. de Launoit Y. Haagensen D. E. Labrie F. (1991). Stimulation of apolipoprotein D secretion by steroids coincides with inhibition of cell proliferation in human LNCaP prostate cancer cells. Cancer Res. 51, 4336–4341. 1868457

[B346] Singh S. A. Andraski A. B. Pieper B. Goh W. Mendivil C. O. Sacks F. M. . (2016). Multiple apolipoprotein kinetics measured in human HDL by high-resolution/accurate mass parallel reaction monitoring. J. Lipid Res. 57, 714–728. 10.1194/jlr.D061432 26862155 PMC4808760

[B347] Skerra A. (2000). Lipocalins as a scaffold. Biochim. Biophys. Acta Prot. Struct. Mol. Enzymol. 1482, 337–350. 10.1016/S0167-4838(00)00145-X 11058774

[B348] Smith K. M. Lawn R. M. Wilcox J. N. (1990). Cellular localization of apolipoprotein D and lecithin:cholesterol acyltransferase mRNA in rhesus monkey tissues by *in situ* hybridization. J. Lipid Res. 31, 995–1004. 10.1016/S0022-2275(20)42739-7 2373967

[B349] Søiland H. Janssen E. A. M. Kørner H. Varhaug J. E. Skaland I. Gudlaugsson E. . (2009a). Apolipoprotein D predicts adverse outcome in women >or=70 years with operable breast cancer. Breast Cancer Res. Treat. 113, 519–528. 10.1007/s10549-008-9955-y 18330697

[B350] Søiland H. Skaland I. Varhaug J. E. Kørner H. Janssen E. A. M. Gudlaugsson E. . (2009b). Co-expression of estrogen receptor alpha and Apolipoprotein D in node positive operable breast cancer–possible relevance for survival and effects of adjuvant tamoxifen in postmenopausal patients. Acta Oncol 48, 514–521. 10.1080/02841860802620613 19107621

[B351] Song J. Y. Lee J. K. Lee N. W. Jung H. H. Kim S. H. Lee K. W. (2008). Microarray analysis of normal cervix, carcinoma *in situ*, and invasive cervical cancer: identification of candidate genes in pathogenesis of invasion in cervical cancer. Int. J. Gynecol. Cancer 18, 1051–1059. 10.1111/j.1525-1438.2007.01164.x 18217980

[B352] Song Y. J. C. Halliday G. M. Holton J. L. Lashley T. O'Sullivan S. S. McCann H. . (2009). Degeneration in different parkinsonian syndromes relates to astrocyte type and astrocyte protein expression. J. Neuropathol. Exp. Neurol. 68, 1073–1083. 10.1097/NEN.0b013e3181b66f1b 19918119

[B353] Søreide J. A. Kolnes J. Skarstein A. Aas T. Kvinnsland S. (1994). Progesterone binding cyst protein in hormone receptor positive breast cancer; a predictive factor for effect of adjuvant tamoxifen treatment. Anticancer Res. 14, 2105–2108. 7840507

[B354] Søreide J. A. Lea O. A. Anda O. Skarstein A. Varhaug J. E. Kvinnsland S. (1991a). Progesterone-binding cyst protein (PBCP) in operable breast cancer: correlations with prognostic factors and predictive value for effect of adjuvant tamoxifen treatment. Anticancer Res. 11, 601–605. 2064314

[B355] Søreide J. A. Lea O. A. Kvinnsland S. (1991b). Progesterone-binding cyst protein (PBCP = GCDFP-24) and steroid hormone receptors as markers of differentiation in breast cancer. Inverse relation of distribution in normal and malignant tissue of the same breast. Anticancer Res. 11, 1323–1326. 1888167

[B356] Soria J. Acera A. Merayo-LLoves J. Durán J. A. González N. Rodriguez S. . (2017). Tear proteome analysis in ocular surface diseases using label-free LC-MS/MS and multiplexed-microarray biomarker validation. Sci. Rep. 7:17478. 10.1038/s41598-017-17536-2 29234088 PMC5727318

[B357] Spreyer P. Schaal H. Kuhn G. Rothe T. Unterbeck A. Olek K. . (1990). Regeneration-associated high level expression of apolipoprotein D mRNA in endoneurial fibroblasts of peripheral nerve. EMBO J. 9, 2479–2484. 10.1002/j.1460-2075.1990.tb07426.x 1695148 PMC552276

[B358] Sreckovic I. Birner-Gruenberger R. Obrist B. Stojakovic T. Scharnagl H. Holzer M. . (2013). Distinct composition of human fetal HDL attenuates its anti-oxidative capacity. Biochim. Biophys. Acta 1831, 737–746. 10.1016/j.bbalip.2012.12.015 23321267

[B359] Starodubtseva N. L. Kononikhin A. S. Bugrova A. E. Chagovets V. Indeykina M. Krokhina K. N. . (2016). Investigation of urine proteome of preterm newborns with respiratory pathologies. J. Proteomics 149, 31–37. 10.1016/j.jprot.2016.06.012 27321582

[B360] Steyrer E. Kostner G. M. (1988). Activation of lecithin-cholesterol acyltransferase by apolipoprotein D: comparison of proteoliposomes containing apolipoprotein D, A-I or C-I. Biochim. Biophys. Acta 958, 484–491. 10.1016/0005-2760(88)90235-4 3124886

[B361] Sugimoto K. Simard J. Haagensen D. E. Labrie F. (1994). Inverse relationships between cell proliferation and basal or androgen-stimulated apolipoprotein D secretion in LNCaP human prostate cancer cells. J. Steroid Biochem. Mol. Biol. 51, 167–174. 10.1016/0960-0760(94)90090-6 7526888

[B362] Sulkava M. Raitoharju E. Levula M. Seppälä I. Lyytikäinen L.-P. Mennander A. . (2017). Differentially expressed genes and canonical pathway expression in human atherosclerotic plaques - Tampere Vascular Study. Sci. Rep. 7:41483. 10.1038/srep41483 28128285 PMC5270243

[B363] Sun D. Zhang H. Guo D. Sun A. Wang H. (2013). Shotgun proteomic analysis of plasma from dairy cattle suffering from footrot: characterization of potential disease-associated factors. PLoS ONE 8:e55973. 10.1371/journal.pone.0055973 23418487 PMC3572155

[B364] Sun H. Wang D. Liu D. Guo Z. Shao C. Sun W. . (2019). Differential urinary proteins to diagnose coronary heart disease based on iTRAQ quantitative proteomics. Anal. Bioanal. Chem. 411, 2273–2282. 10.1007/s00216-019-01668-7 30806752

[B365] Sun Q. Disher M. J. Rustad T. Telian S. A. Andrews P. C. (1998). AP30, a differential protein marker for perilymph and cerebrospinal fluid in middle ear fluid, has been purified and identified as human apolipoprotein D. Biochim. Biophys. Acta 1384, 405–413. 10.1016/S0167-4838(97)00198-2 9659402

[B366] Suresh S. Yan Z. Patel R. C. Patel Y. C. Patel S. C. (1998). Cellular cholesterol storage in the Niemann-Pick disease type C mouse is associated with increased expression and defective processing of apolipoprotein D. J. Neurochem. 70, 242–251. 10.1046/j.1471-4159.1998.70010242.x 9422368

[B367] Tanase-Nakao K. Mizuno K. Hayashi Y. Kojima Y. Hara M. Matsumoto K. . (2019). Dihydrotestosterone induces minor transcriptional alterations in genital skin fibroblasts of children with and without androgen insensitivity. Endocr. J. 66, 387–393. 10.1507/endocrj.EJ18-0494 30787207

[B368] Tao Z. Song W. Zhu C. Xu W. Liu H. Zhang S. . (2017). Comparative transcriptomic analysis of high and low egg-producing duck ovaries. Poult. Sci. 96, 4378–4388. 10.3382/ps/pex229 29053813

[B369] Tapia A. Vilos C. Marín J. C. Croxatto H. B. Devoto L. (2011). Bioinformatic detection of E47, E2F1 and SREBP1 transcription factors as potential regulators of genes associated to acquisition of endometrial receptivity. Reprod. Biol. Endocrinol. 9:14. 10.1186/1477-7827-9-14 21272326 PMC3040129

[B370] Terrisse L. Marcoux K. Do Carmo S. Brissette L. Milne R. Rassart E. (2001). Structure-function relationships of human apolipoprotein D an immunochemical analysis. Life Sci. 70, 629–638. 10.1016/S0024-3205(01)01439-4 11833713

[B371] Terrisse L. Poirier J. Bertrand P. Merched A. Visvikis S. Siest G. . (1998). Increased levels of apolipoprotein D in cerebrospinal fluid and hippocampus of Alzheimer's patients. J. Neurochem. 71, 1643–1650. 10.1046/j.1471-4159.1998.71041643.x 9751198

[B372] Terrisse L. Séguin D. Bertrand P. Poirier J. Milne R. Rassart E. (1999). Modulation of apolipoprotein D and apolipoprotein E expression in rat hippocampus after entorhinal cortex lesion. Brain Res. Mol. Brain Res. 70, 26–35. 10.1016/S0169-328X(99)00123-0 10381540

[B373] Tew S. R. Clegg P. D. Brew C. J. Redmond C. M. Hardingham T. E. (2007). SOX9 transduction of a human chondrocytic cell line identifies novel genes regulated in primary human chondrocytes and in osteoarthritis. Arthritis Res. Ther. 9:R107. 10.1186/ar2311 17935617 PMC2212576

[B374] Thalmann I. Kohut R. I. Ryu J. Comegys T. H. Senarita M. Thalmann R. (1994). Protein profile of human perilymph: in search of markers for the diagnosis of perilymph fistula and other inner ear disease. Otolaryngol. Head Neck Surg. 111, 273–280. 10.1177/01945998941113P117 8084635

[B375] Thomas E. A. Danielson P. E. Nelson P. A. Pribyl T. M. Hilbush B. S. Hasel K. W. . (2001a). Clozapine increases apolipoprotein D expression in rodent brain: towards a mechanism for neuroleptic pharmacotherapy. J. Neurochem. 76, 789–796. 10.1046/j.1471-4159.2001.00027.x 11158250

[B376] Thomas E. A. Dean B. Pavey G. Sutcliffe J. G. (2001b). Increased CNS levels of apolipoprotein D in schizophrenic and bipolar subjects: implications for the pathophysiology of psychiatric disorders. Proc. Natl. Acad. Sci. U.S.A. 98, 4066–4071. 10.1073/pnas.071056198 11274430 PMC31180

[B377] Thomas E. A. Dean B. Scarr E. Copolov D. Sutcliffe J. G. (2003a). Differences in neuroanatomical sites of apoD elevation discriminate between schizophrenia and bipolar disorder. Mol. Psychiatry 8, 167–175. 10.1038/sj.mp.4001223 12610649

[B378] Thomas E. A. George R. C. Danielson P. E. Nelson P. A. Warren A. J. Lo D. . (2003b). Antipsychotic drug treatment alters expression of mRNAs encoding lipid metabolism-related proteins. Mol. Psychiatry 8, 983–993, 950. 10.1038/sj.mp.4001425 14647396

[B379] Thomas E. A. George R. C. Sutcliffe J. G. (2003c). Apolipoprotein D modulates arachidonic acid signaling in cultured cells: implications for psychiatric disorders. Prostaglandins Leukot. Essent. Fatty Acids 69, 421–427. 10.1016/j.plefa.2003.08.014 14623496

[B380] Thomas E. A. Laws S. M. Sutcliffe J. G. Harper C. Dean B. McClean C. . (2003d). Apolipoprotein D levels are elevated in prefrontal cortex of subjects with Alzheimer's disease: no relation to apolipoprotein E expression or genotype. Biol. Psychiatry 54, 136–141. 10.1016/s0006-3223(02)01976-5 12873803

[B381] Thomas E. A. Sautkulis L. N. Criado J. R. Games D. Sutcliffe J. G. (2001c). Apolipoprotein D mRNA expression is elevated in PDAPP transgenic mice. J. Neurochem. 79, 1059–1064. 10.1046/j.1471-4159.2001.00654.x 11739619

[B382] Thomas E. A. Yao J. K. (2007). Clozapine specifically alters the arachidonic acid pathway in mice lacking apolipoprotein D. Schizophr. Res. 89, 147–153. 10.1016/j.schres.2006.08.011 17011169

[B383] Tomarev S. I. Wistow G. Raymond V. Dubois S. Malyukova I. (2003). Gene expression profile of the human trabecular meshwork: NEIBank sequence tag analysis. Invest. Ophthalmol. Vis. Sci. 44, 2588–2596. 10.1167/iovs.02-1099 12766061

[B384] Trieu V. N. Uckun F. M. (2000). Apolipoprotein E and apolipoprotein D expression in a murine model of singlet oxygen-induced cerebral stroke. Biochem. Biophys. Res. Commun. 268, 835–841. 10.1006/bbrc.2000.2205 10679292

[B385] Tristán-Noguero A. Borràs E. Molero-Luis M. Wassenberg T. Peters T. Verbeek M. M. . (2021). Novel protein biomarkers of monoamine metabolism defects correlate with disease severity. Mov. Disord. 36, 690–703. 10.1002/mds.28362 33152132

[B386] Tsukamoto K. Mani D. R. Shi J. Zhang S. Haagensen D. E. Otsuka F. . (2013). Identification of apolipoprotein D as a cardioprotective gene using a mouse model of lethal atherosclerotic coronary artery disease. Proc. Natl. Acad. Sci. U.S.A. 110, 17023–17028. 10.1073/pnas.1315986110 24082102 PMC3801016

[B387] Utermann G. Menzel H.-J. Adler G. Dieker P. Weber W. (1980). Substitution *in vitro* of Lecithin–cholesterol acyltransferase. Eur. J. Biochem. 107, 225–241. 10.1111/j.1432-1033.1980.tb04643.x 6772442

[B388] Utsunomiya T. Ogawa K. Yoshinaga K. Ohta M. Yamashita K. Mimori K. . (2005). Clinicopathologic and prognostic values of apolipoprotein D alterations in hepatocellular carcinoma. Int. J. Cancer 116, 105–109. 10.1002/ijc.20986 15756681

[B389] van den Boom J. Wolter M. Blaschke B. Knobbe C. B. Reifenberger G. (2006). Identification of novel genes associated with astrocytoma progression using suppression subtractive hybridization and real-time reverse transcription-polymerase chain reaction. Int. J. Cancer 119, 2330–2338. 10.1002/ijc.22108 16865689

[B390] Vardi A. Pri-Or A. Wigoda N. Grishchuk Y. Futerman A. H. (2021). Proteomics analysis of a human brain sample from a mucolipidosis type IV patient reveals pathophysiological pathways. Orphanet. J. Rare Dis. 16:39. 10.1186/s13023-021-01679-7 33478506 PMC7818904

[B391] Vázquez J. González L. Merino A. Vizoso F. (2000). Expression and clinical significance of apolipoprotein D in epithelial ovarian carcinomas. Gynecol. Oncol. 76, 340–347. 10.1006/gyno.1999.5678 10684708

[B392] Vieira A. V. Lindstedt K. Schneider W. J. Vieira P. M. (1995). Identification of a circulatory and oocytic avian apolipoprotein D. Mol. Reprod. Dev. 42, 443–446. 10.1002/mrd.1080420411 8607974

[B393] Vijayaraghavan S. Hitman G. A. Kopelman P. G. (1994). Apolipoprotein-D polymorphism: a genetic marker for obesity and hyperinsulinemia. J. Clin. Endocrinol. Metab. 79, 568–570. 7913935 10.1210/jcem.79.2.7913935

[B394] Vizoso F. Díez-Itza I. Sánchez L. M. Tuya A. F. Ruibal A. López-Otín C. (1994). Relationship between serum prolactin levels and protein composition of breast secretions in nonlactating women. J. Clin. Endocrinol. Metab. 79, 525–529. 8045972 10.1210/jcem.79.2.8045972

[B395] Vizoso F. Sánchez L. M. Díez-Itza I. Luz Lamelas M. López-Otín C. (1992). Factors affecting protein composition of breast secretions from nonlactating women. Breast Cancer Res. Treat. 23, 251–258. 10.1007/BF01833522 1463865

[B396] Vizoso F. J. Rodriguez M. Altadill A. González-Diéguez M. L. Linares A. González L. O. . (2007). Liver expression of steroid hormones and Apolipoprotein D receptors in hepatocellular carcinoma. World J. Gastroenterol. 13, 3221–3227. 10.3748/wjg.v13.i23.3221 17589901 PMC4436608

[B397] Vogt M. Skerra A. (2001). Bacterially produced apolipoprotein D binds progesterone and arachidonic acid, but not bilirubin or E-3M2H. J. Mol. Recognit. 14, 79–86. 10.1002/1099-1352(200101/02)14:1<79::AIDJMR521>3.0.CO;2-4 11180564

[B398] Wade N. M. Tollenaere A. Hall M. R. Degnan B. M. (2009). Evolution of a novel carotenoid-binding protein responsible for crustacean shell color. Mol. Biol. Evol. 26, 1851–1864. 10.1093/molbev/msp092 19414522

[B399] Waldner A. Dassati S. Redl B. Smania N. Gandolfi M. (2018). Apolipoprotein D concentration in human plasma during aging and in Parkinson's disease: a cross-sectional study. Parkinsons Dis 2018:3751516. 10.1155/2018/3751516 29780571 PMC5892211

[B400] Walsh N. Dale J. McGraw K. J. Pointer M. A. Mundy N. I. (2012). Candidate genes for carotenoid coloration in vertebrates and their expression profiles in the carotenoid-containing plumage and bill of a wild bird. Proc. Biol. Sci. 279, 58–66. 10.1098/rspb.2011.0765 21593031 PMC3223654

[B401] Wang J. Scholtens D. Holko M. Ivancic D. Lee O. Hu H. . (2013). Lipid metabolism genes in contralateral unaffected breast and estrogen receptor status of breast cancer. Cancer Prev. Res. 6, 321–330. 10.1158/1940-6207.CAPR-12-0304 23512947

[B402] Wang S.-C. M. Myers S. Dooms C. Capon R. Muscat G. E. O. (2010). An ERRbeta/gamma agonist modulates GRalpha expression, and glucocorticoid responsive gene expression in skeletal muscle cells. Mol. Cell. Endocrinol. 315, 146–152. 10.1016/j.mce.2009.07.012 19631715

[B403] Warden C. H. Diep A. Taylor B. A. Lusis A. J. (1992). Localization of the gene for apolipoprotein D on mouse chromosome 16. Genomics 12, 851–852. 10.1016/0888-7543(92)90325-M 1572665

[B404] Watanabe Y. Hirao Y. Kasuga K. Tokutake T. Kitamura K. Niida S. . (2020). Urinary apolipoprotein C3 is a potential biomarker for Alzheimer's disease. Dement. Geriatr. Cogn. Dis. Extra 10, 94–104. 10.1159/000509561 33082773 PMC7548924

[B405] Wei Y.-J. Huang Y.-X. Zhang X.-L. Li J. Huang J. Zhang H. . (2008). Apolipoprotein D as a novel marker in human end-stage heart failure: a preliminary study. Biomarkers 13, 535–548. 10.1080/13547500802030363 18979643

[B406] Weinberg R. B. (1994). Identification of functional domains in the plasma apolipoproteins by analysis of inter-species sequence variability. J. Lipid Res. 35, 2212–2222. 10.1016/S0022-2275(20)39927-2 7897319

[B407] West R. B. Harvell J. Linn S. C. Liu C. L. Prapong W. Hernandez-Boussard T. . (2004). Apo D in soft tissue tumors: a novel marker for dermatofibrosarcoma protuberans. Am. J. Surg. Pathol. 28, 1063–1069. 10.1097/01.pas.0000126857.86186.4c 15252314

[B408] Wiklund O. Fager G. olofsson S. O. Wilhelmsson C. Bondjers G. (1980). Serum apolipoprotein levels in relation to acute myocardial infarction and its risk factors–determination of apolipoprotein D. Atherosclerosis 37, 631–636. 10.1016/0021-9150(80)90070-2 7459006

[B409] Wu M. Li Q. Wang H. (2021). Identification of novel biomarkers associated with the prognosis and potential pathogenesis of breast cancer via integrated bioinformatics analysis. Technol. Cancer Res. Treat. 20, 1–16. 10.1177/1533033821992081 33550915 PMC7876582

[B410] Xu L. Jia F. Luo C. Yu Q. Dai R. Li X. (2018). Unravelling proteome changes of chicken egg whites under carbon dioxide modified atmosphere packaging. Food Chem. 239, 657–663. 10.1016/j.foodchem.2017.06.128 28873618

[B411] Yamashita K. Upadhyay S. Osada M. Hoque M. O. Xiao Y. Mori M. . (2002). Pharmacologic unmasking of epigenetically silenced tumor suppressor genes in esophageal squamous cell carcinoma. Cancer Cell 2, 485–495. 10.1016/S1535-6108(02)00215-5 12498717

[B412] Yan L. Park J. Y. Dillinger J.-G. De Lorenzo M. S. Yuan C. Lai L. . (2012). Common mechanisms for calorie restriction and adenylyl cyclase type 5 knockout models of longevity. Aging Cell 11, 1110–1120. 10.1111/acel.12013 23020244 PMC3646327

[B413] Yang C.-L. Kurczab T. Down G. Kealey T. Langlands K. (2005). Gene expression profiling of the ageing rat vibrissa follicle. Br. J. Dermatol. 153, 22–28. 10.1111/j.1365-2133.2005.06550.x 16029322

[B414] Yang C. Y. Gu Z. W. Blanco-Vaca F. Gaskell S. J. Yang M. Massey J. B. . (1994). Structure of human apolipoprotein D: locations of the intermolecular and intramolecular disulfide links. Biochemistry 33, 12451–12455. 10.1021/bi00207a011 7918467

[B415] Yang C. Y. Kim T. W. Weng S. A. Lee B. R. Yang M. L. Gotto A. M. (1990). Isolation and characterization of sulfhydryl and disulfide peptides of human apolipoprotein B-100. PNAS 87, 5523–5527. 10.1073/pnas.87.14.5523 2115173 PMC54357

[B416] Yao J. K. Thomas E. A. Reddy R. D. Keshavan M. S. (2005). Association of plasma apolipoproteins D with RBC membrane arachidonic acid levels in schizophrenia. Schizophr. Res. 72, 259–266. 10.1016/j.schres.2004.05.007 15560970

[B417] Yao Y. Vieira A. (2002). Comparative 17beta-estradiol response and lipoprotein interactions of an avian apolipoprotein. Gen. Comp. Endocrinol. 127, 89–93. 10.1016/S0016-6480(02)00032-1 12161206

[B418] Yin J. Spillman E. Cheng E. S. Short J. Chen Y. Lei J. . (2021). Brain-specific lipoprotein receptors interact with astrocyte derived apolipoprotein and mediate neuron-glia lipid shuttling. Nat. Commun. 12:2408. 10.1038/s41467-021-22751-7 33893307 PMC8065144

[B419] Yokoo H. Oishi T. Isoda K. Nakazato Y. Toyokuni S. (2007). Oxidative stress is related to the formation of Antoni B patterns and eosinophilic hyaline droplets in schwannomas. Neuropathology 27, 237–244. 10.1111/j.1440-1789.2007.00772.x 17645238

[B420] Yoshida K. Cleaveland E. S. Nagle J. W. French S. Yaswen L. Ohshima T. . (1996). Molecular cloning of the mouse apolipoprotein D gene and its upregulated expression in Niemann-Pick disease type C mouse model. DNA Cell Biol. 15, 873–882. 10.1089/dna.1996.15.873 8892759

[B421] Yu R.-H. Zhang X.-Y. Xu W. Li Z.-K. Zhu X.-D. (2020). Apolipoprotein D alleviates glucocorticoid-induced osteogenesis suppression in bone marrow mesenchymal stem cells via the PI3K/Akt pathway. J. Orthop. Surg. Res. 15:307. 10.1186/s13018-020-01824-1 32771037 PMC7414572

[B422] Zeng C. Spielman A. I. Vowels B. R. Leyden J. J. Biemann K. Preti G. (1996). A human axillary odorant is carried by apolipoprotein D. Proc. Natl. Acad. Sci. U.S.A. 93, 6626–6630. 10.1073/pnas.93.13.6626 8692868 PMC39076

[B423] Zenkel M. Pöschl E. von der Mark K. Hofmann-Rummelt C. Naumann G. O. H. Kruse F. E. . (2005). Differential gene expression in pseudoexfoliation syndrome. Invest. Ophthalmol. Vis. Sci. 46, 3742–3752. 10.1167/iovs.05-0249 16186358

[B424] Zhang J. He Q. Liu Q. Y. Guo W. Deng X. M. Zhang W. W. . (2007). Differential gene expression profile in pig adipose tissue treated with/without clenbuterol. BMC Genomics 8:433. 10.1186/1471-2164-8-433 18039366 PMC2231380

[B425] Zhang S. X. Bentel J. M. Ricciardelli C. Horsfall D. J. Haagensen D. E. Marshall V. R. . (1998). Immunolocalization of apolipoprotein D, androgen receptor and prostate specific antigen in early stage prostate cancers. J. Urol. 159, 548–554. 10.1016/S0022-5347(01)63981-8 9649289

[B426] Zhang X. Li D. Duan S. Duan Y. Chen Q. Li X. . (2006). Analysis of the association between Apolipoprotein D and schizophrenia. Neuropsychobiology 54, 40–44. 10.1159/000095740 16966838

[B427] Zhang Y. Chen K. Sloan S. A. Bennett M. L. Scholze A. R. O'Keeffe S. . (2014). An RNA-sequencing transcriptome and splicing database of glia, neurons, and vascular cells of the cerebral cortex. J. Neurosci. 34:11929. 10.1523/JNEUROSCI.1860-14.2014 25186741 PMC4152602

[B428] Zheng W. Mast N. Saadane A. Pikuleva I. A. (2015). Pathways of cholesterol homeostasis in mouse retina responsive to dietary and pharmacologic treatments. J. Lipid Res. 56, 81–97. 10.1194/jlr.M053439 25293590 PMC4274074

[B429] Zhou J. Ng S. Adesanya-Famuiya O. Anderson K. Bondy C. A. (2000). Testosterone inhibits estrogen-induced mammary epithelial proliferation and suppresses estrogen receptor expression. FASEB J. 14, 1725–1730. 10.1096/fj.99-0863com 10973921

[B430] Zhou X. Wang J. Lu Y. Chen C. Hu Y. Liu P. . (2020). Anti-depressive effects of Kai-Xin-San on lipid metabolism in depressed patients and CUMS rats using metabolomic analysis. J. Ethnopharmacol. 252:112615. 10.1016/j.jep.2020.112615 31991203

[B431] Zhu Z. Zhang H. Luo G. Xu N. Pan Z. (2015). Association between the ABCC11 gene polymorphism and the expression of apolipoprotein D by the apocrine glands in axillary osmidrosis. Mol. Med. Rep. 11, 4463–4467. 10.3892/mmr.2015.3274 25633187

[B432] Zou S. Zhang J. for Alzheimer's Disease Neuroimaging Initiative Chen W. (2019). Subtypes based on six apolipoproteins in non-demented elderly are associated with cognitive decline and subsequent tau accumulation in cerebrospinal fluid. J. Alzheimers Dis. 72, 413–423. 10.3233/JAD-190314 31594221

