# Supplementary figures and images for: The Lipocalin Apolipoprotein D Functional Portrait: A Systematic Review
Source: Front Physiol. 2021 Oct 7;12:738991. doi: 10.3389/fphys.2021.738991 (PMC8530192; doi:10.3389/fphys.2021.738991)

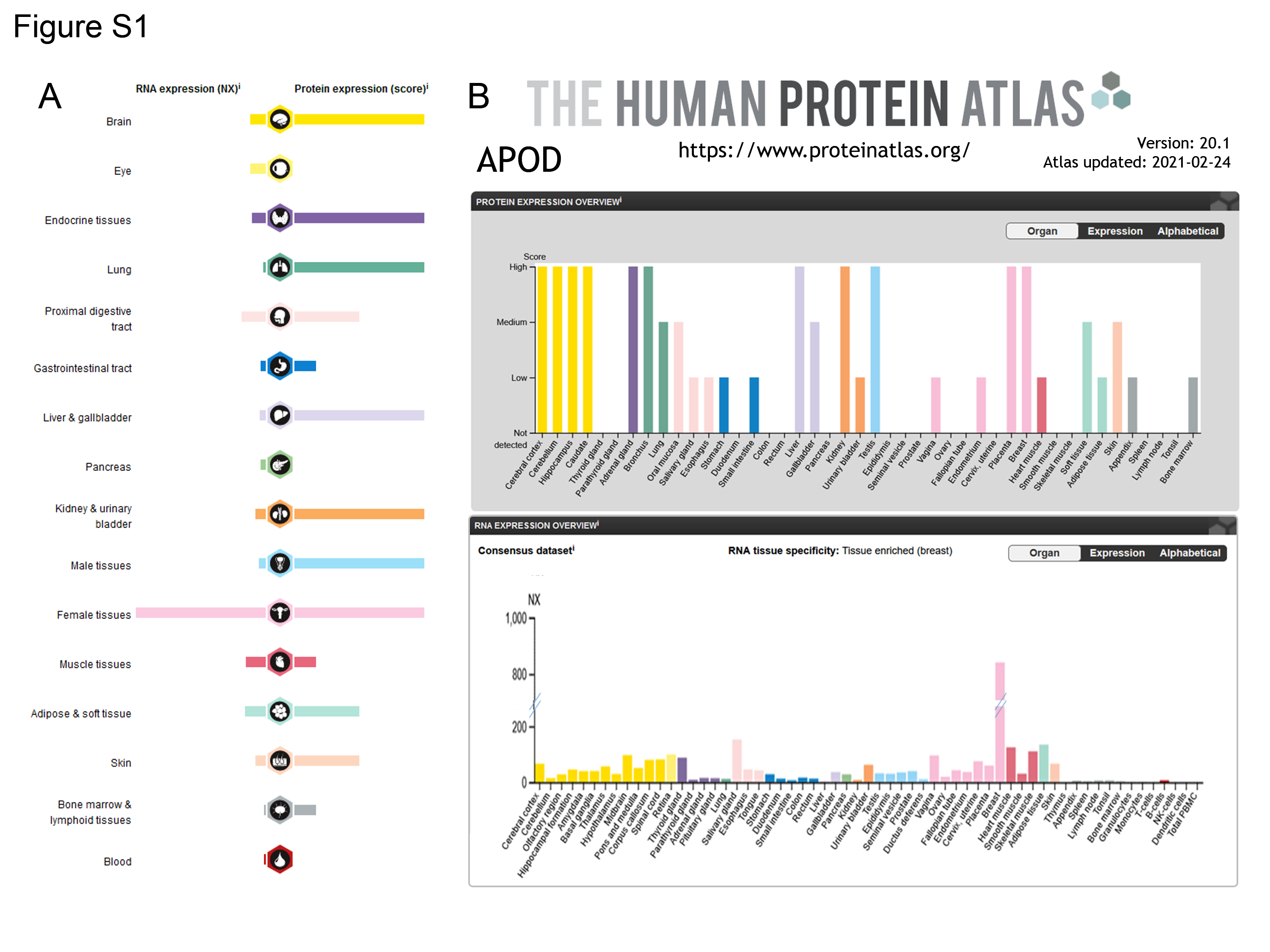

Supplement: Supplementary Figure 1 — ApoD mRNA and protein expression summary of the Human Protein Atlas. [file Image_1.TIF]

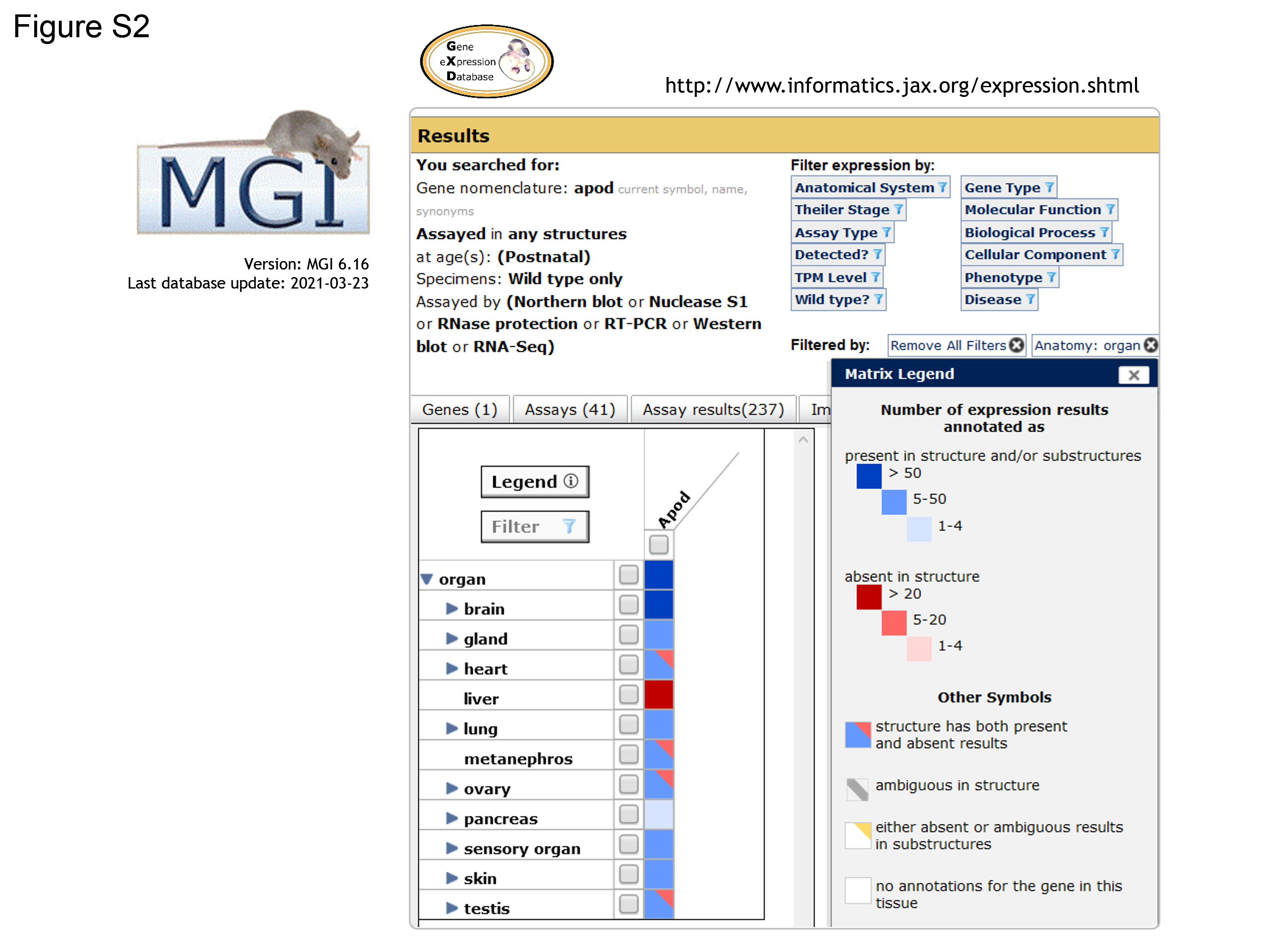

Supplement: Supplementary Figure 2 — ApoD mRNA and protein expression summary of the Mouse Gene Expression Database. [file Image_2.TIF]
